# Genome-wide analysis of the NAC transcription factor family and their expression during the development and ripening of the *Fragaria* × *ananassa* fruits

**DOI:** 10.1371/journal.pone.0196953

**Published:** 2018-05-03

**Authors:** Enriqueta Moyano, Félix J. Martínez-Rivas, Rosario Blanco-Portales, Francisco Javier Molina-Hidalgo, Pablo Ric-Varas, Antonio J. Matas-Arroyo, José Luis Caballero, Juan Muñoz-Blanco, Antonio Rodríguez-Franco

**Affiliations:** 1 Departamento de Bioquímica y Biología Molecular, Campus de Rabanales, Edificio Severo Ochoa, Universidad de Córdoba, Córdoba, Spain; 2 Instituto de Hortofruticultura Subtropical y Mediterránea “La Mayora” (IHSM-UMA-CSIC), Departamento de Biología Vegetal, Universidad de Málaga, Málaga, Spain; University of Naples Federico II, ITALY

## Abstract

NAC proteins are a family of transcription factors which have a variety of important regulatory roles in plants. They present a very well conserved group of NAC subdomains in the N-terminal region and a highly variable domain at the C-terminus. Currently, knowledge concerning NAC family in the strawberry plant remains very limited. In this work, we analyzed the NAC family of *Fragaria vesca*, and a total of 112 NAC proteins were identified after we curated the annotations from the version 4.0.a1 genome. They were placed into the ligation groups (pseudo-chromosomes) and described its physicochemical and genetic features. A microarray transcriptomic analysis showed six of them expressed during the development and ripening of the *Fragaria* x *ananassa* fruit. Their expression patterns were studied in fruit (receptacle and achenes) in different stages of development and in vegetative tissues. Also, the expression level under different hormonal treatments (auxins, ABA) and drought stress was investigated. In addition, they were clustered with other NAC transcription factor with known function related to growth and development, senescence, fruit ripening, stress response, and secondary cell wall and vascular development. Our results indicate that these six strawberry NAC proteins could play different important regulatory roles in the process of development and ripening of the fruit, providing the basis for further functional studies and the selection for NAC candidates suitable for biotechnological applications.

## Introduction

Strawberry (*Fragaria x ananassa*) is a non-climacteric model plant of economic importance due both its edibility and healthy values. It contains numerous compounds such as vitamin C, folic acid, anthocyanins, flavonoids, and phenolic acids with important biological activities useful in the prevention of diseases related to the oxidative stress [1]. Ripening is an essential process determining fruit quality and involves many physiological and biochemical modifications. In strawberry, the most noticeable changes include alterations of fruit size, texture, color, and aroma that are associated to the activity of many different genes already identified [2]. The regulation of strawberry fruit ripening is rather complex. It is known that auxins and ABA are the main hormones controlling this process, but other plant hormones as jasmonate, ethylene and gibberellins are also involved [2, 3]. There is also some evidence for a relevant sucrose-mediated ripening [4]. Many different transcription factors (TFs) are also playing key roles in these processes [2, 3]. Some of them, like the master regulator FaMYB10 [5], FaEOBII [6], FaDOF2 [7] and others [2, 8] have already been examined. However, it remains unknown the role played for the NAC (NAM, ATAF1/2 and CUC2) proteins, one of the largest families of plant-specific TFs present in a wide range of species [9,10].

The NAC family proteins play important regulatory roles in plants including flowering [11], root formation [12], senescence [10, 13], shoot branching [14], responses to diverse abiotic and biotic stress such as salinity, drought and chilling [9, 15] and interaction with fungal and bacterial pathogens [16, 17]. In addition, NAC TFs are involved in various aspects of fruit ripening. For example, the tomato NAC-NOR (non-ripening) is an important TF whose mutation lead to a non-ripening phenotype affected in many ripening-related aspects [18]. The tomato SlNAC1 and SlNAC4 proteins regulate carotenoid biosynthesis, ethylene emission, and the softening of the fruit [19, 20, 21]. In banana, MaNAC1/MaNAC2 regulate fruit ripening via physical interaction with ethylene signaling components [22]. A NAC TF termed BLOOD form a heterodimer with other NACs to induce the transcription of *PpMYB10*, leading to the accumulation of anthocyanin in peach fruits [23]. Recently, LcNAC1 has been proposed as a regulator of fruit senescence in litchi [24]. However, the number of studies relating to NAC TFs in fruit development and specifically in non-climacteric fruits is scarce.

Most of NAC proteins have a conserved DNA-binding NAC domain of approximately 150 amino acids found mainly at the N-terminus of the protein, and a highly variable transcriptional regulatory region in the C-terminal region [25, 26]. The NAC domain is usually divided into five subdomains (A–E). The C and D subdomains are positively charged and allow the TF to bind to the DNA. The A subdomain can promote functional dimerization, and the variable subdomains B and E may be involved in the many distinctive functions of the NAC proteins. The C-terminal transcription regulatory region can either activate or repress transcription, and in some cases, can also bind to other proteins [10, 25, 26]. Several specific and conserved motifs for certain subgroups of NAC subfamilies have been determined at the C-terminal region but diverge among the different NAC subfamilies. This variation has also been occasionally correlated with the distinct functions of NAC proteins [17, 26]. A transmembrane motif is sometimes present in the C-terminal region of many NACs proteins that allow the translocation of the protein to the nucleus after proteolytic cleavage and under different stress conditions [10, 26, 27, 28].

The availability of the strawberry whole genome sequence is a valuable resource to identify gene families and gain further insight into their functions. Gene-wide analyses are used as a foundation to dissect and deduce the functions of determined genes. A recent example is the study of the strawberry *WRKY* genes implied in pathogen resistance and stress tolerance [29]. In this study, we present a genome-wide analysis of the NAC TFs in the diploid *Fragaria vesca* that includes a strawberry database search using the genome version 4.0.a1, an annotation curation, a phylogeny study, a description of the gene structure, and an assignation to their ligation group (pseudo-chromosoma) locations. Additionally, we analyzed several strawberry NAC proteins that, according a microarray analysis [3], are implied in the development and ripening of the commercial *Fragaria x ananassa* fruit. A total of 112 *NAC* genes have been found in strawberry. Six of them were expressed throughout the fruit development and/or ripening and were characterized through quantitative real time PCR analyses in the receptacle, achenes and in various vegetative tissues. In addition, we investigated their expression level under different hormonal treatments and stress conditions (auxins, ABA, and drought). Finally, some biological functions to these fruit-related proteins were putatively assigned after performing alignments with Arabidopsis NAC sequences and with a set of 61 NAC proteins with known functions from different plant species. Our results show that the six ripening-related NAC TF studied are suitable candidate genes for running further functional genomics research to develop biotechnological approaches to improve strawberry fruit quality attributes and their postharvest shelf life.

## Materials and methods

### Identification and curation of putative *Fragaria* NAC genes

A list of putative *NAC*-containing genes from *Fragaria vesca* was obtained from the public version 4.0.a1 strawberry genomic database (http://bit.ly/Fragaria4genome). We searched within the annotations looking for the term “NAC” using the bash grep command to find all the NAC-related sequences. In addition, a BlastP was performed using several of the sequences containing the NAC domains within *F*. *vesca* genome against the same genome using the facilities found in the rosacea.org web. The information found in the plant transcription factor database (PlantTFDB) was initially discarded because it is still based on the outdated version v1.1 annotation of the *Fragaria* genome. These genes and their structures can be used for the study of the commercial octoploid *Fragaria* × *ananassa* plants, since both plants shares the same genome structure and higher than 98% identity at the nucleotide level in all genes analyzed to date [30]. This agrees with higher than 93% of paired-end lectures of *Fragaria x ananassa* mapped with Tophat to the *F*. *vesca* reference genome using standard settings (results not shown).

The initial list of NAC annotations needed to be curated since the current version 4.0.a1 of the assembled strawberry genome retains still some uncertainties. We removed those sequences that even being initially annotated as a NAC protein, lacked the whole or had an almost incomplete NAC multi-domain. This was assessed by analyzing the presence of the NAC subdomains using MEME (http://meme-suite.org/tools/meme), by scanning into the InterPro and the Pfam domains databases, and by using the information provided in the NCBI Conserved Domains Database (CDD). A summary of discarded sequences can be found in S1 and S2 Tables.

### Analysis of the *Fragaria* NAC proteins and chromosomal location

Gene sequences and their location in the strawberry chromosomes were obtained from the public genome database. Version 4.0.a1 was used, which was assembled through Bionano optical maps and SMART (Pacific Bioscience) sequencing. Amino acid lengths, molecular weights, isoelectric points, and other physicochemical features of the NAC proteins were obtained using the EMBOSS *pepstats* (http://www.ebi.ac.uk/Tools/emboss) and the ExPASy *protparam* programs (http://expasy.org). A genomic map including the location of the NAC genes was drawn using the Mapchart v2.30 software [31].

### Domain and motif finding in the *Fragaria* NAC family

An initial search for putative motifs and/or domains that were in common among the strawberry NAC proteins was done using the MEME program, version 4.11.0 [32]. This was set in normal mode, for any number of occurrences per sequence, and allowing up to 15 different motifs per sequence with a length ranged between 5 to 100 amino acids. Reliability of the MEME predicted trees was tested by the neighbor-joining method with 1000 bootstrap replicates using the IQ-tree web server [33]. Other motifs and domains present in the NAC family were investigated following different approaches. We run an InterProScan search (http://www.ebi.ac.uk/Tools/pfa/iprscan) [34] looking for already featured domains and motifs. This information was completed with another scan done with the Conserved Domain Architecture Retrieval Tools (CDART) database, which is useful because it finds protein similarities using sensitive protein domain profiles rather than strict rules of identity homology (https://www.ncbi.nlm.nih.gov/Structure/lexington/lexington.cgi) [35]. Since some NAC sequences contain relevant transmembrane helices, a prediction of these domains was performed with the TMHMM v2.0 server (http://www.cbs.dtu.dk/services/TMHMM/) [36].

### Assignation of putative functions to the *Fragaria* fruit related *NAC* genes

Current annotations of the *F*. *vesca* database do not contain any specific mention to the putative biological functions played by the NAC proteins, but do include cross references to orthologous *Arabidopsis thaliana* genes. Thus, we accessed the TAIR (https://www.arabidopsis.org/) database to gather information about the biological role played by these sequences. Only those functions that were directly inferred after the isolation of mutants and from direct interactions with other proteins (as revealed by double hybrid experiments and the like) were selected. We did not consider expression as this was pervasive in almost all the cases. We also obtain information about the putative biological function played for many other NAC proteins from other plants that were described into the gene annotations and/or published articles after running a BlastP using the NCBI non-redundant (nr) protein database. Thus, the information about the biological role played by NAC proteins was completed after running an extensive bibliography searching. S3 Table contains the NAC sequences considered with indication of the plant species, gen names, the Uniprot, GenBank and TAIR accessions, and the list of publications (doi and pubmed URLs) where the biological functions are described. The corresponding amino acid sequences in fasta format are in S4 Table.

Initially, as an approach taken by many other genome-wide analysis studies [17, 25], a multiple sequence alignment was run with the fruit related *Fragaria* NAC proteins along all Arabidopsis NAC sequences. This was performed with Clustal Omega that uses a guided tree and a hidden Markov model (HMM) to cluster similar sequences using Gonnet as the default transition matrix and a gap opening and extension penalties of 6 and 1 bits, respectively [37, 38]. We executed this program with the maximum value of 5 iterations, set into configuration. This program cluster sequences based on protein sequence similarity and not in phylogenetical relationships, and this could be crucial when attempting to assign and compare biological functions. The program provides the alignment tree (*.ph) that was represented using the FigTree v.1.4.3 program (http://tree.bio.ed.ac.uk/software/figtree/). Then, the gene functions obtained from the TAIR database and genbank annotations were manually assigned to these represented sequences. To determine whether the NAC subdomains and/or the domains present in the C-terminal region or both regions of the protein are interacting together to drive the biological function of the NAC proteins, we aligned three datasets by separate with the Arabidopsis sequences: i) the whole NAC protein gene sequences; ii) the amino terminal regions containing only the NAC multi-domains, and iii) the carboxyl terminal (TR) regions lacking the NAC multi-domains. The amino terminal region harboring the NAC domain was identified after running MEME to recognize the limits of the conserved domains and considering the information provided into the CDD database. Eventually, we run a Clustal Omega alignment with the entire NAC sequences from any plants whose NAC biological functions were clearly defined.

### Plant material

Strawberry plants (*Fragaria* × *ananassa* Duch. cv. Camarosa, an octoploid cultivar) were grown under field conditions in Huelva (SW of Spain). Plants used for hormone treatments were grown in plant chambers under controlled conditions at 25 °C with a 16/8h light/dark photoperiod. *Fragaria* × *ananassa* fruits were harvested at different developmental stages: small-sized green fruits (G1, 2–3 g), full-sized green fruits (G3, 4–7 g), white fruits (W, 5–8 g), full-ripe red fruits (R, 6–10 g) overripe fruits (OR, 6–10 g) and senescent fruits (SE, 6–10 g). Overripe fruits were harder and dark redder than red ones, meanwhile senescent fruits were softer. Vegetative tissues, such as runners, petals, flowers, and expanding leaves were harvested from the same plants. All collected tissues were immediately frozen in liquid nitrogen and stored at -80 °C until use.

### RNA isolation

Total RNA was isolated and purified from at least three independent pools of strawberry fruits at different growth and ripening stages and from vegetative tissues in accordance with [39]. Total RNA was treated with DNase I (RNase free; Invitrogen) according to the manufacturer’s instructions. RNA samples were considered free of DNA when no amplicons corresponding to the analyzed genes were obtained in a standard PCR reaction.

### Auxin treatment

Achenes of two sets of 50 full-sized green (G3) fruits were carefully removed from their receptacles with the help of a scalpel. One set of de-achened G3 fruits was wrapped with 1 mL of lanolin paste that contained 1mM of the synthetic auxin 1-Naphthaleneacetic acid (NAA) and 1% (w/v) of dimethyl sulphoxide. The other set of G3 de-achened fruits (control group) were treated with the same lanolin paste containing dimethyl sulphoxide but lacking NAA. Auxin treatments and sample collection were performed according to [5].

### Nordihydroguaiaretic acid treatment

For ABA-related experiments, green-white fruits were used. A 100 μM of an aqueous solution of 1-Nordihydroguaiaretic acid (NDGA) was injected into the receptacles with a syringe to fully block ABA biosynthesis [40]. NDGA is a strong inhibitor of 9 cis-epoxy-carotenoid dioxygenase, an enzyme required for the biosynthesis of ABA. The ABA content of these samples have been already measured and published [6].

### Water stress (drought) treatment

For the drought stress treatment, we followed established protocols [41]. Fruits from green to white stages were cut along with their pedicels. Half of them were subjected to drought conditions by maintaining the pedicels outdoors under controlled conditions. Pedicels of control fruits not subjected to water stress were immersed in sterile tubes containing Murashige and Skoog liquid medium with 20g/L of sucrose that was renewed every other day [5].

### Microarray analysis and real-time qPCR validation of data

RNA isolated from these experiments has been analyzed through a one-color Agilent microarray analysis using a chip containing more than 32000 different strawberry genes [3]. From these data, an initial selection of six *NAC* genes related to the development and ripening of the strawberry fruit was done using a threshold cutoff consisting in a fold change higher than 2 (relative to the green stage), an adjusted p-value <= 0.01, and an arbitrary expression intensity >= 700 units.

Validation of microarray expression data on these six *NAC* genes was done using a Bio-Rad iCycler Real Time PCR device using SYBR-Green [42]. Primers were designed to obtain an amplified band ranging between 90 and 120 nucleotides with the NCBI Pick Primers WEB service that included a BLAST alignment with the entire strawberry genome to ensure a selective and specific amplification for each of the genes. The amplification efficiency of each qRT-PCR and the melting curves of the final products were also analyzed to test for the existence of a single amplification peak corresponding to unique molecular specie. Primer sequences are shown in S5 Table. cDNA first strand synthesis was done using 2 μg of total RNA with the iScript kit (Bio-Rad) following the manufacturer’s instructions. Each reaction of the qRT-PCR was carried out by triplicate and their corresponding *C*_t_ values normalized using as reference a 26S–18S interspaced strawberry RNA gene [42, 43, 44, 45]. All these values were used to determine the relative changes of gene expression in the samples as compared with controls in accordance with [46].

## Results and discussion

### Identification, annotation, sequence curation, and chromosomal location of the strawberry NAC family

A total of 117 NAC-containing genes are defined in the annotations of the current published strawberry genome (V 4.0.a1). No new NAC genes were added after running discontinuous BlastP searches against the whole strawberry genome using several randomly chosen *Fragaria* NAC sequences as queries. Since the 4.0.a1 annotation of the *Fragaria vesca* genome still retain certain questionable annotations, we curated these sequences and discarded 5 of them because they lacked either the whole or had a substantially incomplete NAC multi-domain. These discarded sequences showed no parallelism in subdomains composition to other NAC sequences described in more mature genomic assemblies like that of Arabidopsis. S1 Table shows the sequences that were discarded and the reason of that, and S2 Table shows the Pfam pairwise alignment of those discarded gene sequences. Thus, the predicted *NAC* genes in the strawberry genome were reduced to a total of 112 *NAC* genes (S6 Table). This number of NAC genes in strawberry agrees with those described in other dicotyledonous plants like tomato (104 genes) [47], Arabidopsis (106 genes) [48], and soybean (101 genes) [49].

**Fig 1:**
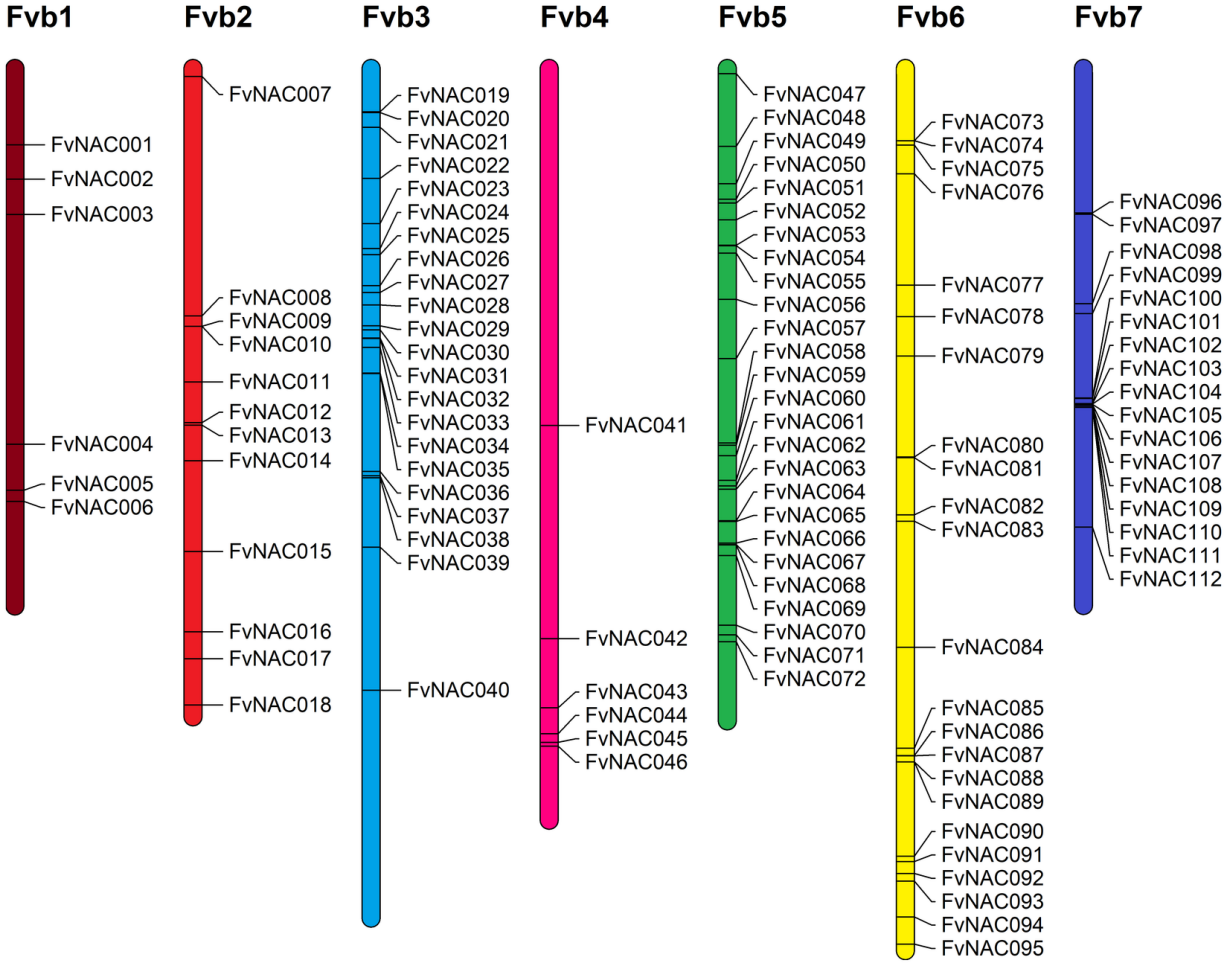
Ligation Group (FvH) distribution of the curated collection of the strawberry *NAC* genes. The putative *NAC* genes were renamed from *FvNAC001* to *FvNAC112*, based on their placement into the Ligation Groups (FvH) or pseudo-chromosomes. The lengths of the drawings are proportional to the actual pseudo-chromosome lengths. FvH1 has a length of 24.25Mbp nucleotides and this can be used as a reference. S7 Table shows the correspondence between the current gene names provided in the *Fragaria* genome database and these newly assigned and ordered names, and the actual length in bases of all pseudo-chromosomes and contigs of the current V4.0.a1 of the genome.

The position of the 112 *Fragaria NAC* genes were mapped onto the Ligation Groups (pseudo-chromosomes) FvH1 to FvH7. Then, they were renamed by their order into the chromosomes as *FvNACXXX*, where XXX is a number ranging between 001 and 112 (Fig 1). LG5 contained the highest number of *NAC* genes (26) whereas FvH1 and FvH4 only contained 6 sequences. The current version 4.0.a1 of the genome contains 22 unlocated contigs, but none of them contains a *NAC* gene.

### Domain and motif analysis of the strawberry NAC family

**Fig 2:**
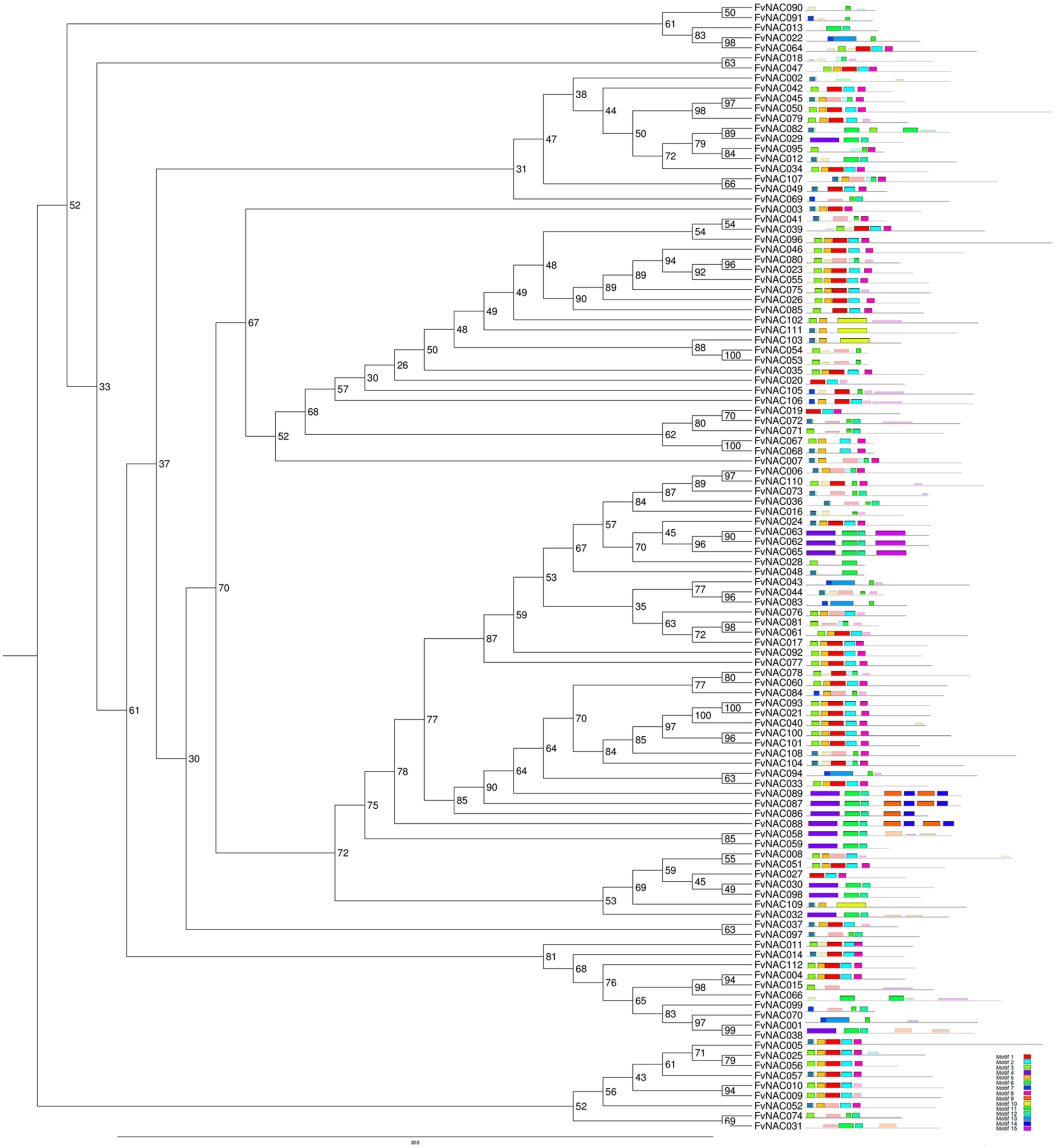
Phylogenetic relationship and conserved subdomain composition of the 112 NAC strawberry proteins using MEME. The phylogenetic tree was constructed with the protein sequences with the IQ-tree program using 1000 bootstrappings. Domain analysis was performed with the MEME web service as indicated in Material and methods. A colored box represents each individual subdomain. Grey lines represent sequences that do not share common domains. Detailed amino acid sequence information for each subdomain is provided in S8 Table.

MEME analysis of the 112 strawberry NAC proteins identified 15 subdomains that are in common among these sequences (Fig 2). A detailed amino acid composition of these subdomains is shown in S8 Table. As expected, the N-terminal region contained most of the NAC subdomains share a high degree of conservation. Two main big clades appear with a close evolutionary relationship and uniform subdomain compositions. The first group is mainly composed of subdomains 3, 5, 1, 3 and 6, respectively. The second group is composed of subdomains 4, 9/15, 8, 13, 14 and 7. Previously, 10 subdomains were described in the tea plant and in potato, 15 in cassava and 20 in tomato, all of them sharing a similar structure, where the conserved domains were found mainly in the N-terminal region of the proteins [47, 50, 51, 52].

A very low number of common domains among the 112 sequences were discovered in the C-terminal regions of the proteins using MEME. This is due to this program does not use the InterPro or Pfam databases to find already known and featured domains. In addition, a domain search analysis done with any of the strawberry NAC sequences using the CDART database clearly shows that this C-terminal region is highly variable and contains many different domains probably accounting for their many distinctive functions. Predicted physiochemical and genetic characteristics of each of the NAC proteins and genes are summarized in S7 Table. This was done using the coding sequence annotation included in the database without considering putative variants or isoforms. The predicted protein-coding genes have from 1 to 8 exons with an average of 3 exons, and the proteins ranged from 169 to 715 amino acids in length. The isoelectric point varied from 3.9 to 10.3, giving an indirect indication of the many different cell locations where they could be expressed. These values are like those found in the NAC population of another species.

Membrane associated transcription factors have become a main point in research, because of their role in regulating gene expression in plants [10, 25, 26]. The presence of putative transmembrane domains in the strawberry NAC genes was investigated using TMHMM server v. 2.0. Four proteins, FvNAC005, FvNAC008, FvNAC050 and FvNAC107, contained a α-helical transmembrane domain (designated as NTL) at the C-terminal of the protein (S9 Table). A number of NTLs have been identified in other plant species, including Arabidopsis (18 NTLs), maize (7 NTLs), grape (6 NTLs) and rice (5 NTLs) [10, 27]. NTLs are proposed to play important roles in plant growth and development and in responses to stress [10, 26, 27, 28]. However, none of the NTLs in strawberry are related to the development and ripening of the fruit, so they were not further characterized.

### Expression analysis of *NAC* genes during the development and ripening of strawberry fruits

According a microarray analysis [3] (GEO database, accession number GSE95300), a total of six *Fragaria x ananassa NAC* genes (*FaNAC006*, *FaNAC021*, *FaNAC022*, *FaNAC035*, *FaNAC042* and *FaNAC092*) are differentially expressed during the development and ripening of the strawberry fruit under the cutoff indicated in Material and methods. S10 Table shows the expression level of these *NAC* genes in *Fragaria x ananassa* and the statistical significance after running a Bayesian fit and a test of false discovery according to [53] using the R-package limma.

**Fig 3:**
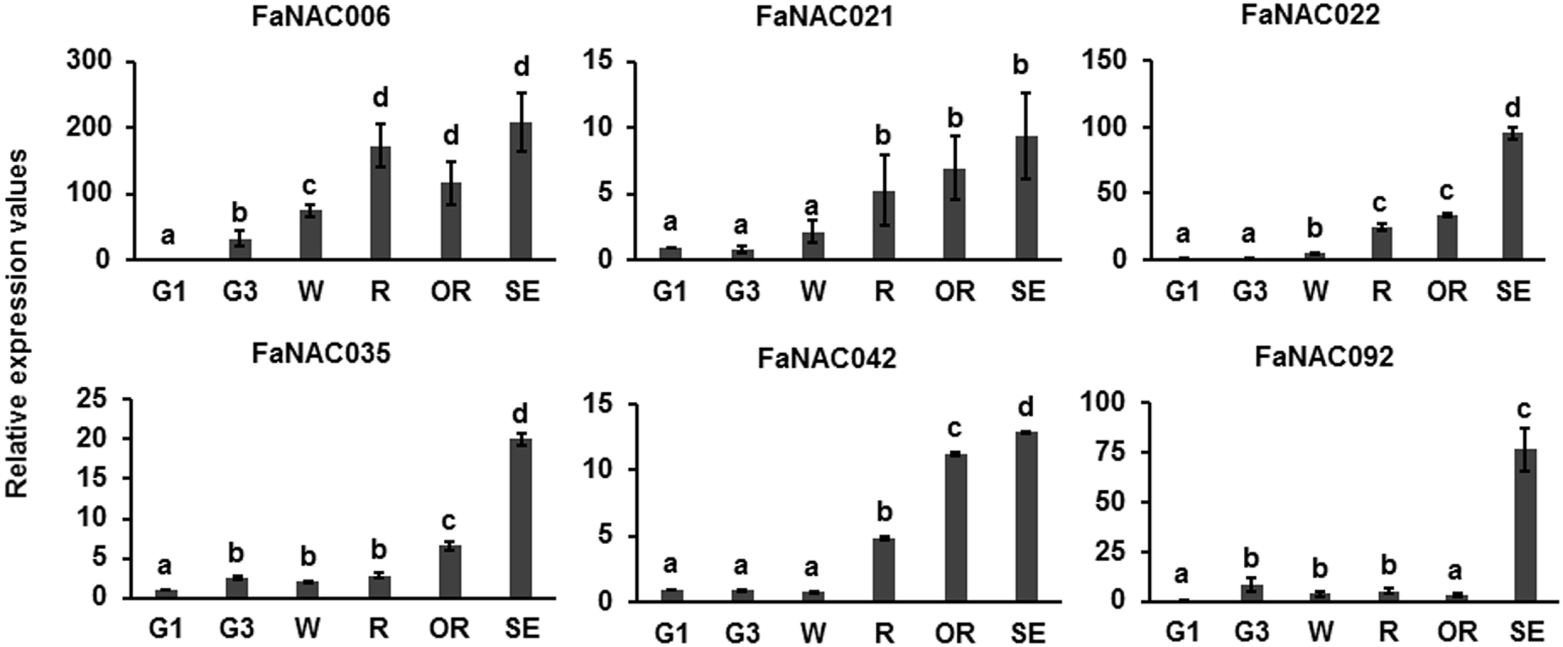
Developmental expression of the strawberry ripening-related *NAC* genes in fruit receptacles. qRT-PCR results were obtained using specific primers for *NAC* genes. Quantification is based on C_t_ values. Relative expression values were calculated in relation to G1 fruits receptacles C_t_ value in all cases, which was assigned an arbitrary value equal to unity. G1, green stage 1; G3, green stage 3; W, white stage; R, red stage; OR, overripe stage and SE, senescent stage. Data are a mean of three independent experiments. One-way ANOVA determined statistical significance. Letters indicate significant differences (p < 0.05, Scheffe post-hoc test).

The transcription profiles of these six genes were analyzed and validated at the different development and ripening stages of strawberry fruit receptacles by qRT-PCR. Except for *FaNAC92*, all the fruit-related *NAC* genes analyzed showed a similar expression pattern, with a relative low level of expression during the development stages (G1 to W), and a clear induction throughout the ripening and senescent stages (R to SE) (Fig 3). *FaNAC092* expression was detected mainly in the senescent stage, showing a substantial expression in this stage respect to the R and OR stages. The expression patterns analyzed by qRT-PCR coincided with those of the microarray analyses, thereby validating these results, and were like those of many strawberry ripening-related genes [5, 6, 7, 46, 48, 54, 55, 56], reinforcing that these 6 genes could be involved in these processes. *FaNAC092* expression pattern directly indicates that this gene could play a role in the senescence of the strawberry fruit. Many miRNAs are involved in post-harvest senescence of the strawberry fruit. One of them, miR164, targets three *NAC* genes: NAC domain transcriptional regulator family protein (gene00971.1-v1.0-hybrid that is FvH4_3g19410.1 in V4.0.a1), NAC domain containing protein 38 (gene26043.1-v1.0-hybrid that is FvH4_5g14670.1 in V4.0.a1) and NAC domain containing protein 87 (gene04424.1-v1.0-hybrid that is FvH4_3g43100.1 in V4.0.a1) [57]. Interestingly, we found that the gene corresponding to the NAC domain containing protein 87 is *FaNAC092*, which suggests that this gene would be involved in regulating fruit senescence independently that the fruit ripens on the plant or after harvesting.

**Fig 4:**
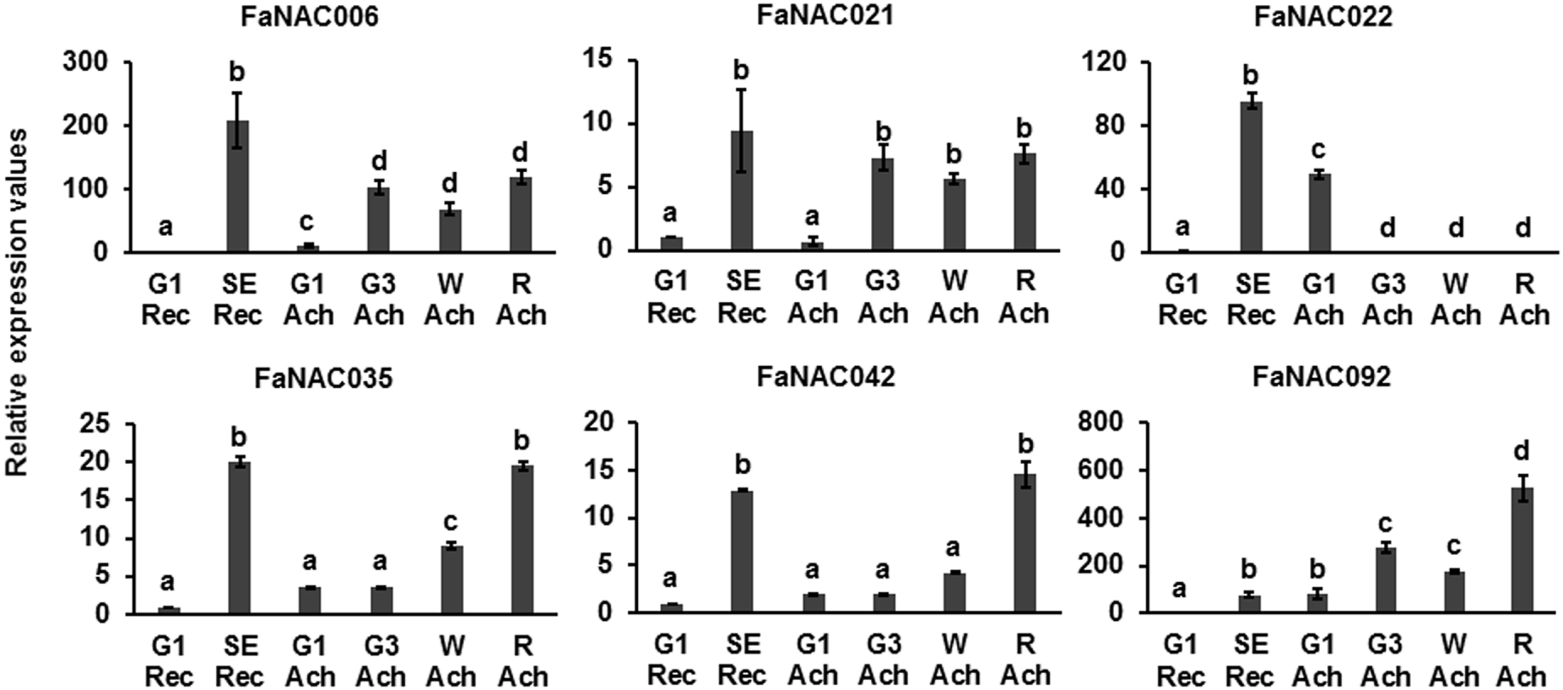
Developmental expression of the strawberry ripening-related *NAC* genes in fruit achenes determined by qRT-PCR. Relative expression values were calculated in relation to the fruit receptacle C_t_ at the G1 stage in all cases, which was assigned an arbitrary value equal to unity. G1 Rec: fruit receptacles at the G1 stage; SE Rec: fruit receptacles at the ripen stage; G1 Ach: achenes of G1 fruits; G3 Ach: achenes of G3 fruits; W Ach: achenes of white fruits; and R Ach: achenes of ripen strawberries. Data are a mean of three independent experiments. One-way ANOVA determined statistical significance. Letters indicate significant differences (p < 0.05, Scheffe post-hoc test).

We also analyzed gene expression in the achenes at various stages (Fig 4). Expression of *FaNAC035* and *FaNAC042* increased in achenes throughout all the stages with their highest levels at the ripen stage. A similar expression pattern was found for *FaNAC092*, whose expression was even higher in achenes when compared with those of senescent fruit receptacles. However, *FaNAC006* and *FaNAC021* were expressed in all developmental and ripening stages studied, and no distinctive pattern was observed. *FaNAC022* was expressed in achenes only at an early stage of development (G1 stage).

**Fig 5:**
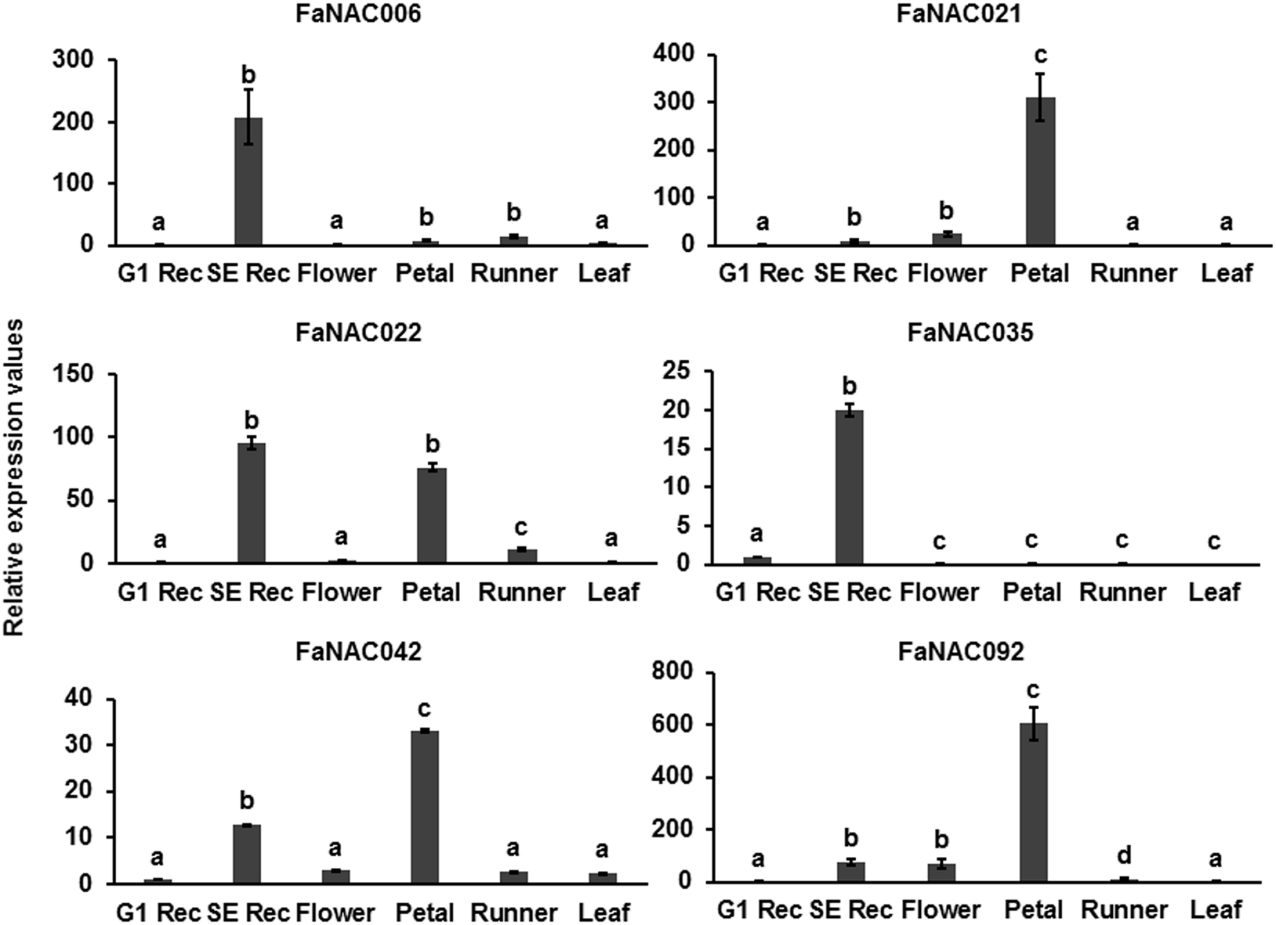
Tissue specificity expression of the strawberry ripening-related *NAC* genes determined by qRT-PCR. Relative expression values were calculated in relation to the fruit receptacle C_t_ at the G1 stage in all cases, which was assigned an arbitrary value equal to unity. G1 Rec: fruit receptacles at the G1 stage; SE Rec: fruit receptacles at the senescent stage; vegetative tissue used were flowers, petals, runners, and leaves. Data are a mean of three independent experiments. One-way ANOVA determined statistical significance. Letters indicate significant differences (p<0.05, Scheffe post-hoc test).

To determine whether these genes are fruit specific, expression of the *NAC* genes was analyzed in vegetative tissues such as flowers, petals, runners, and leaves (Fig 5). *FaNAC021*, *FaNAC022*, *FaNAC042* and *FaNAC092* showed elevated levels of expression mainly in petals. *FaNAC021*, *FaNAC042* and *FaNAC092* showed higher expression in petals than in receptacles at the senescent stage. *FaNAC035* was not expressed in any of the vegetative tissues analyzed. Thus, *FaNAC006* and *FaNAC035* are the only *NAC* genes specifically expressed in fruit, although a very low level of *FaNAC006* expression was also observed in runners. Thus, *FaNAC021*, *FaNAC022*, *FaNAC042* and *FaNAC092* genes could be involved in regulating other processes in the plant in addition to fruit ripening. In fact, many studies have shown that a single *NAC* gene can function as regulator of different processes [9, 17]. The tomato *SlNAC1* is a ripening-related gene expressed in other plant tissues and has a wide influence on fruit ripening. It controls lycopene and ethylene biosynthesis, and indirectly the tomato fruit softening by altering the synthesis of ABA [19]. They could be regulating the same process in different tissues. Two strawberry TFs, R2R3-MYB TF (FaEOBII) and FaDOF2 showed a similar pattern with a high expression in ripen receptacles and petals regulating the production of eugenol, a volatile phenylpropanoid that contributes to the flower and ripe fruit scents [6, 7].

### Hormonal regulation of strawberry ripening-related *NAC* genes

**Fig 6:**
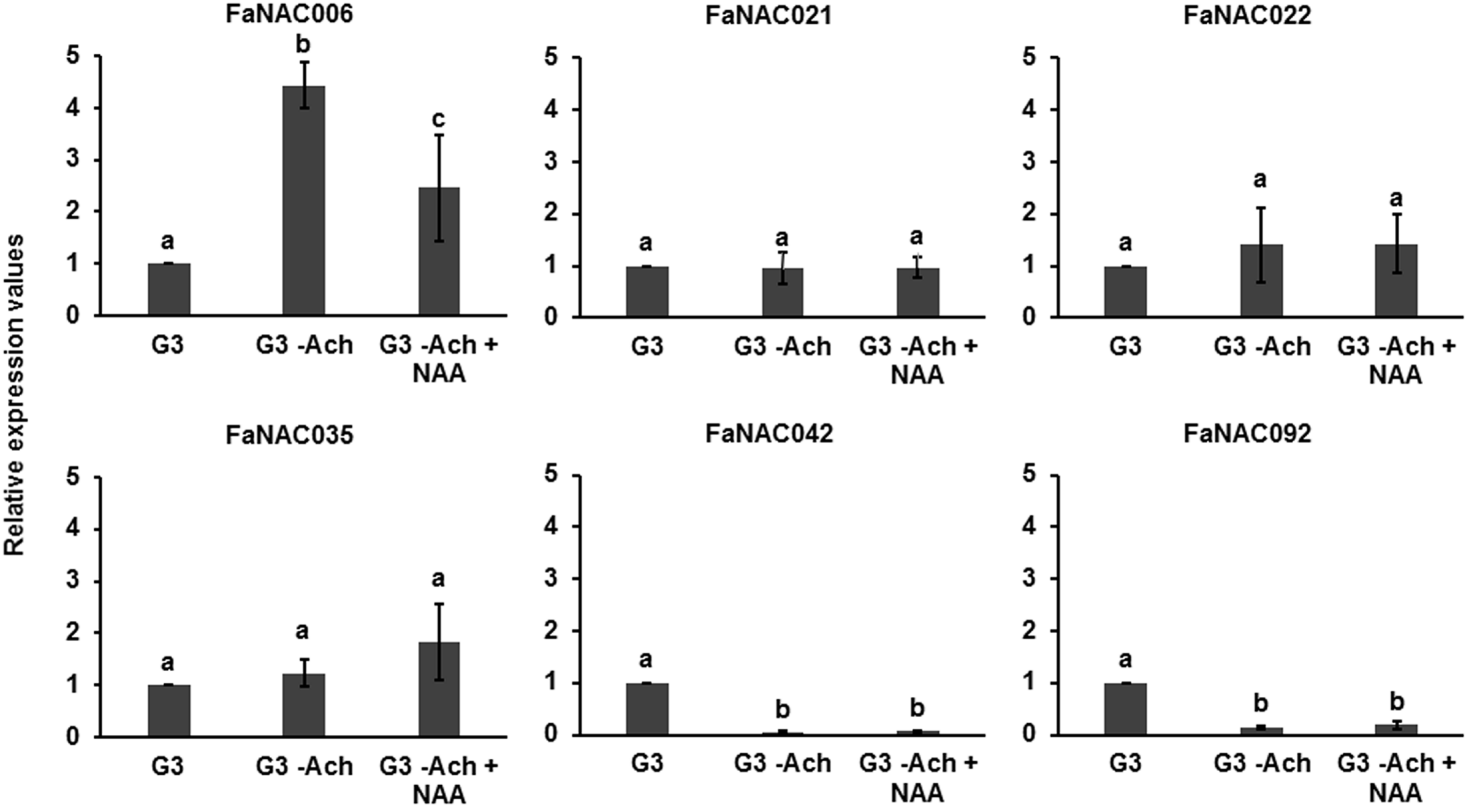
Auxin regulated expression of the strawberry ripening-related *NAC* genes determined by qRT-PCR. After auxin treatment, relative expression values were calculated in relation to the C_t_ of control fruits at the G3 stage, which was assigned an arbitrary value equal to the unity. G3: fruit at the G3 stage; G3 –Ach: G3 fruit receptacle without achenes for 5 days; G3 -Ach + NAA: G3 fruit receptacle without achenes plus NAA for 5 days (added at day zero). Data are a mean of three independent experiments. One-way ANOVA determined statistical significance. Letters indicate significant differences (p<0.05, Scheffe post-hoc test).

**Fig 7:**
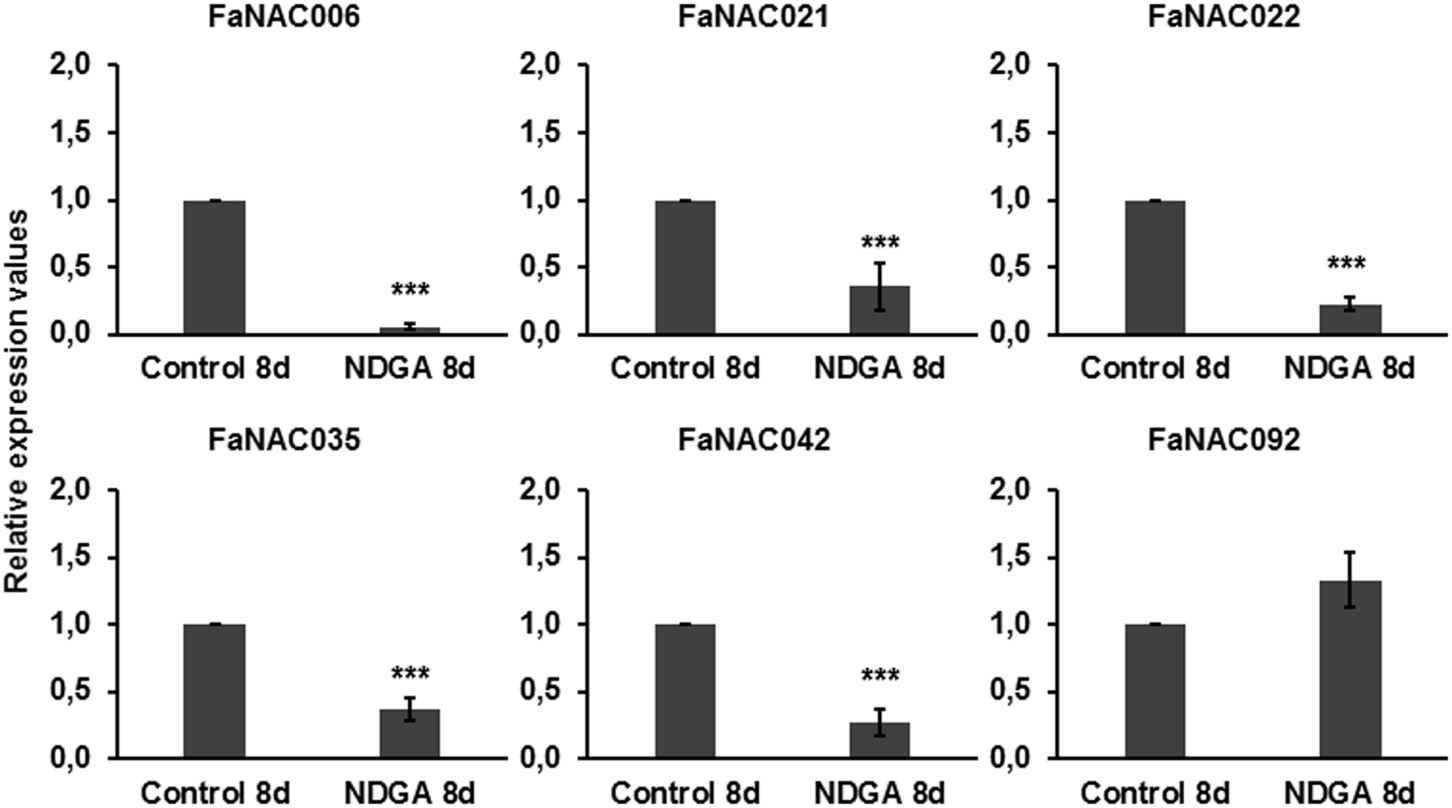
Effect of the application of the ABA biosynthesis inhibitor NDGA on the expression of the strawberry ripening-related *NAC* genes determined by qRT-PCR. After treatment, relative expression values were calculated in relation to untreated control C_t_, which was assigned an arbitrary value equal to the unity. Control: G-W fruit injected with H_2_O; NDGA: G-W fruits injected with NDGA (100 μM). Both samples were harvested 8 days after beginning of treatment. Mean values ± SD of three independent experiments are shown. Student’s t-test determined statistical significance with respect to reference sample. (***) p-value < 0.001.

In strawberry, ABA and auxins are the main hormones involved in the regulation of the fruit. Auxins, produced by immature achenes, stimulate receptacle growth and seem to prevent fruit ripening, whereas ABA is responsible of the ripening process [2, 3]. We analyzed the effect of both hormones on the expression of the fruit-related *NAC* genes (Figs 6 and 7). To investigate whether the expression of the selected *NAC* genes was under the control of auxins, we removed the achenes from strawberry G3 stage fruits and measured expression after 5 days (Fig 6). Only *FaNAC006* was induced in de-achened receptacles, and this increase was attenuated by the external application of the synthetic auxin 1-Naphthaleneacetic acid (NAA). This suggests that auxins can repress *FaNAC006*. However, the expression of *FaNAC021*, *FaNAC022* and *FaNAC035* did not change in de-achened receptacles, suggesting that auxins are not regulating their expression. Expression of *FaNAC042* and *FaNA092* diminished in de-achened receptacles in comparison to control indicating that auxins could be inducing them. A recent publication shows that there are some auxin inducible genes expressed in ripen fruits in strawberry [58]. An increase in the expression of both genes however, was not observed when we added the auxin NAA (Fig 6). This means that auxins are not actually regulating their expression, and that the induction of this gene could be more related to a stress caused by wounding. This possibility should be addressed in the future.

Two different approaches were taken to investigate the effect of ABA on the expression of the fruit related *NAC* genes. First, we examined the expression after injecting to the receptacles 1-Nordihydroguaiaretic acid (NDGA), an inhibitor of the biosynthesis of ABA [59]. Second, we subjected receptacles to drought as this treatment increase endogenous ABA biosynthesis and induce premature ripening [5, 60]. The actual ABA content levels in receptacles were determined in these experiments (S11 Table). Fig 7 shows that expression of *FaNAC006*, *FaNAC021*, *FaNAC022*, *FaNAC035* and *FaNAC042* genes substantially diminished after NDGA addition. This clearly indicates that ABA regulates their expression and gives support that these genes are involved in the ripening process. However, *FaNAC092* gene expression was not affected by this treatment.

**Fig 8:**
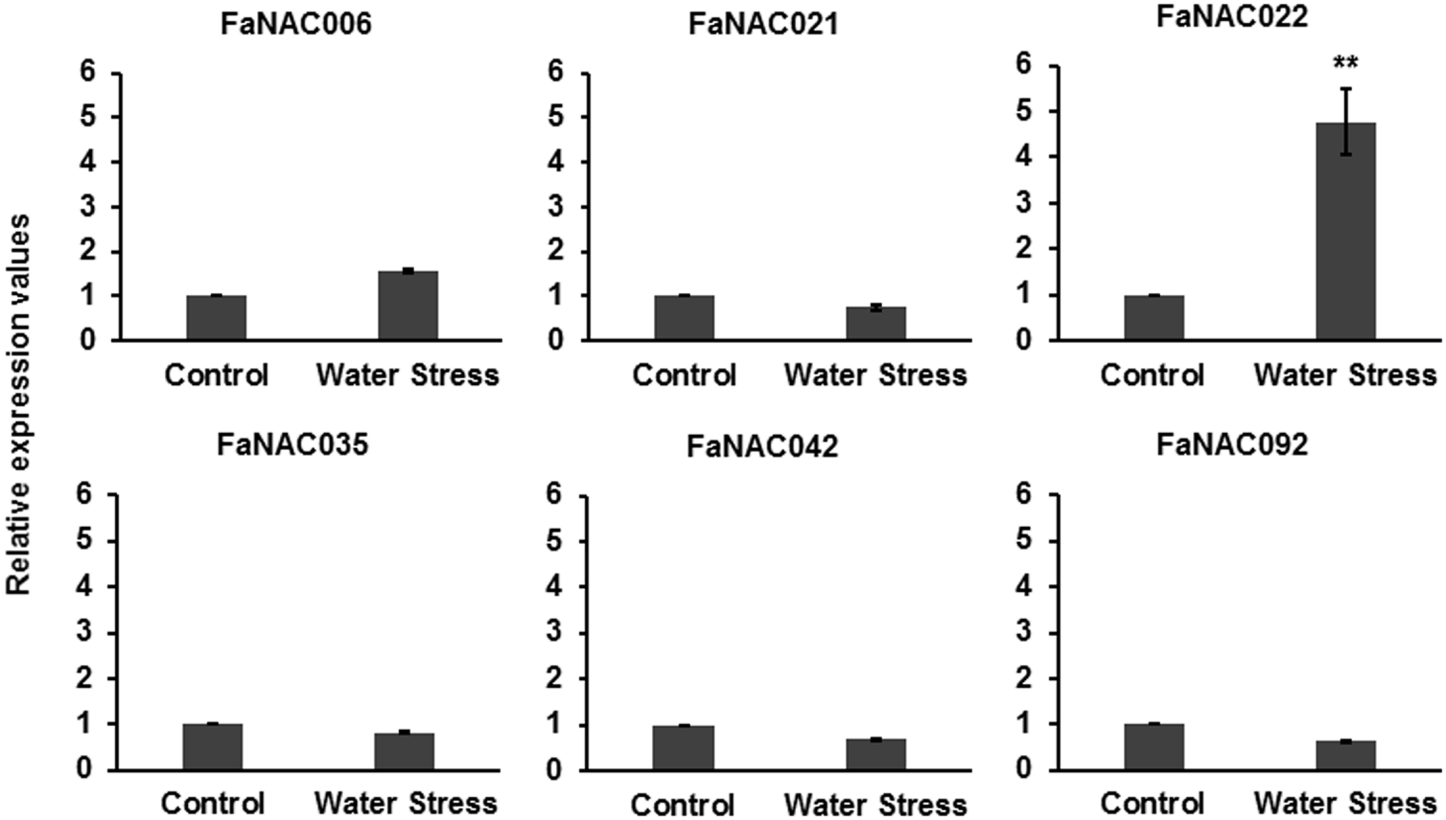
Effect of drought on the expression of the strawberry ripening-related *NAC* genes determined by qRT-PCR. After treatment, relative expression values were calculated in relation to untreated control C_t_, which was assigned an arbitrary value equal to the unity. Control: G-W fruits with their pedicels immersed in MS medium with sucrose; Water Stress: fruits with their pedicels kept in the air. Both samples were harvested 4 days after beginning of treatment. Mean values ± SD of three independent experiments are shown. Student’s t-test determined statistical significance with respect to reference sample. (**) p-value < 0.001.

In contrast, no noticeable changes were observed in the expression of these genes (except for *FaNAC022*) when the fruits were submitted to drought (Fig 8). So, it appears that drought-induced water stress did not activate the expression of *FaNAC006*, *FaNAC021*, *FaNAC035* and *FaNAC042*, even though it was accompanied by a noticeable increase in ABA content (S11 Table). This can be explained if we assume that the severity and the duration of this stress was not enough to induce the expression of these genes. In addition, it suggests that the expression mechanism of these *NAC* genes in response to ABA and dehydration are more complex than expected, and that some other factors are required. It is worth noting that the experiments of drought stress were done with fruits detached from the plant by maintaining the pedicels into MS medium with sucrose (control fruits) or exposed to the air (drought). Sucrose induces fruit ripening by regulating ABA levels through mechanisms remaining largely unknown [4]. Recent studies of a strawberry ABA-stress-ripening TF, *FaASR*, shown that it works downstream of a common transduction pathway for ABA and sucrose signals in fruit ripening, supporting the relationship of ABA and sucrose in the ripening mechanism of strawberry [61]. Also, it has been reported that sucrose-modulated *FaASR* transcriptional levels could be mediated via both an ABA-dependent and ABA-independent pathways [61]. Thus, sucrose can influence the expression of these strawberry *NAC* genes. Further studies need to be done to probe this.

### Assignation of putative biological functions to the ripening related strawberry NAC proteins

The *Fragaria* annotation included in the public database assigns Arabidopsis orthologous to each of the FvNAC proteins (S7 Table) but does mention neither any putative biological role nor the degree of identity and/or homology between these proteins. An alignment with orthologous Arabidopsis sequences is usually done to assign putative biological functions to new proteins. This approach has revealed functions to NAC proteins in several plants, such those implied in salt stress in *Cucumis melo* [62], drought responses in maize [63], abiotic stresses and stress-related phytohormone treatments in *Brachypodium distachyon* [64] and ethylene-responsive signaling in banana [22]. Other studies have assigned a wider range of functions to the NAC proteins using the same approach [52, 65, 66, 67, 68]. Thus, we run a Clustal Omega alignment with all NAC protein sequences from Arabidopsis along the six developmental and ripening related NAC TFs from strawberry to predict their functions. The proteins functions obtained from the Arabidopsis TAIR database were annotated at the resulting tree (S1 Fig). We found a small cluster of NAC proteins implied in the biosynthesis and/or development of vascular tissue and cell walls (namely VTCW cluster, blue sequences) (At2G46770, At4G36160, At2G18060, At1G12260, At5G62380 and At1G71930), a minor cluster implied in water balance (namely WB cluster, green sequences) (At4G27410.1, At4G27410.2, and At1G52880), and a minor cluster implied in root cap formation (namely RC cluster, orange sequences) (AT1G79580.1 AT1G79580.2, AT1G79580.2, AT4G10350 and AT1G33280). No clustering was observed for NAC proteins implied in other biological roles such as senescence, flowering, and meristematic development that were dispersedly represented throughout all the tree alignment.

FvNAC021 and FvNAC035 were closely located to the Arabidopsis WB cluster. This suggests that both proteins could be playing a role in water balance and/or stress. In fact, some porins implied in water balance are induced during the ripening [41]. However, the others strawberry FvNAC TFs were not clearly associated to any functional cluster, although FvNAC006 and FvNAC092 were near to the VTCW clade, suggesting a similar role for these proteins. In fact, a degradation of the cell walls that lead to the softening of the fruit takes place at the onset of the ripening of strawberries [2]. In addition, FvNAC022 and FvNAC042, although fell out of the VTCW cluster, are orthologous of AT4G28500.1 and AT5G13180.1 respectively, with similar functions to the VTCW cluster. AT4G28500.1 (SND2) regulates genes involved in secondary cell wall formation in Arabidopsis fiber [69] and AT5G13180.1 is a NAC TF (VNI2) repressing xylem cell formation [70]. Thus, FvNAC022and FvNAC042 could also be related to the ripening-associated textural changes that involved modifications to the cell-wall components [2].

Since it has been described that the NAC domain itself can drive for the biological function [9, 17, 26], we look for an alternative relationship after aligning only the N-terminal regions that contains this domain. The alignment with only the NAC domains showed the three (VTCW, RC and WB) clades (S2 Fig). This means that these functions could be driven in Arabidopsis by the NAC domain itself and not for the C-terminal portion of the NAC proteins. However, only the WB clade was present when we aligned only the C-terminal region harboring the TR domain of NAC proteins (S3 Fig). This suggests that in the case of the WB clade, there is a cooperation or interaction among the NAC subdomains and the C-terminal portion of the proteins. Nevertheless, most of the 6 FvNAC sequences were always dispersed over any of the phylogenetic trees. Therefore, a putative function could not be inferred with some degree of certainty by using only Arabidopsis sequences.

**Fig 9:**
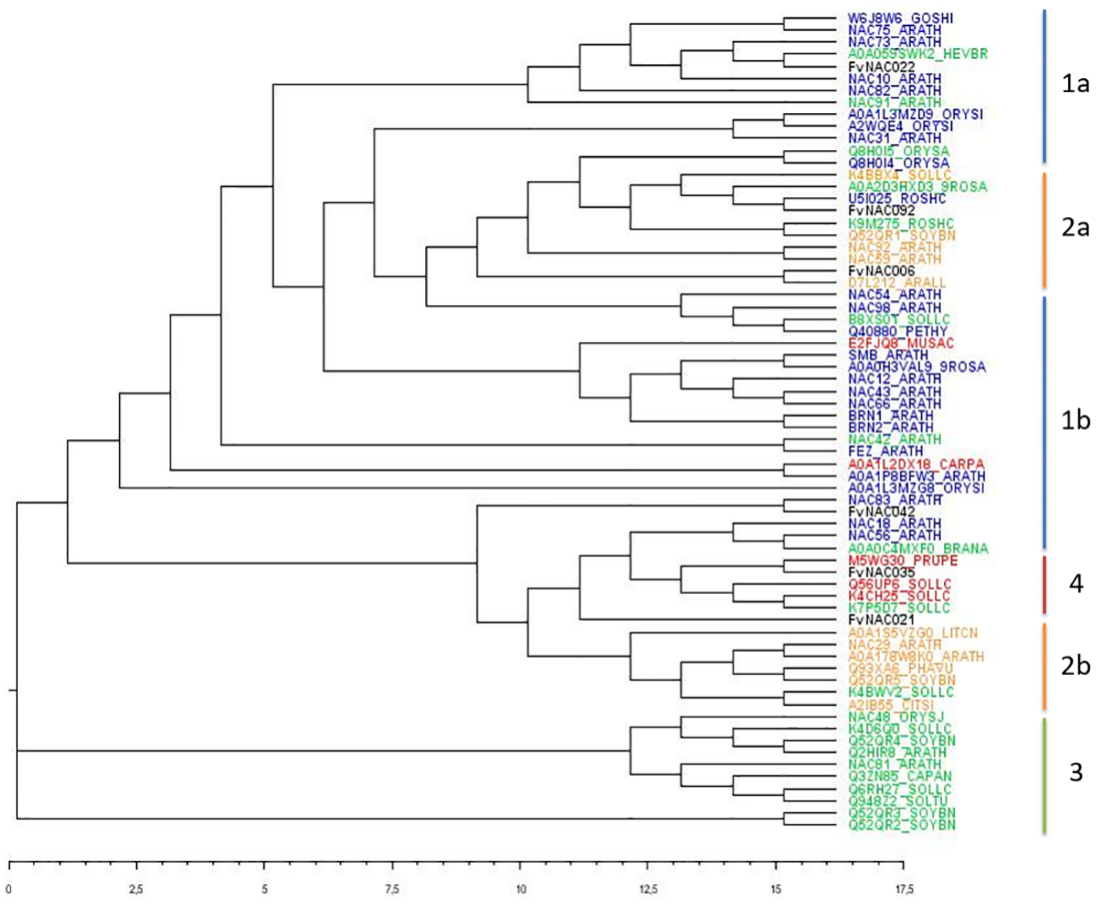
Clustal Omega cladogram with the alignment of the six strawberry FvNAC proteins and NAC proteins from different plant species with known functions. Each protein sequence is named by their UniProt code. A direct URL link to these sequences to gain easy access to the UniProt, Genbank and TAIR databases can be found in S3 Table. The full length of the proteins was used for the alignment. Colors indicate protein function: green, stress response; blue, plant growth and development and cell wall metabolism; orange, senescence; red, fruit ripening. The lower scale represents the relative genetic distance. Cluster 1a and 1b contains NAC proteins mainly involved in growth and development, and secondary cell wall development; cluster 2a and 2b contains NAC proteins mainly involved in senescence; cluster 3 contains NAC proteins mainly involved in stress response and cluster 4 includes NAC proteins mainly involved in fruit ripening.

Thus, the study was extended to include 61 NAC proteins of known function from different plant species (S3 Table). The alignment of the proteins showed that they are grouped into 4 clusters according to their functions (Fig 9). Cluster 1a and 1b contains mostly NAC proteins involved in growth and secondary cell wall development; cluster 2a and 2b contains mainly NAC proteins related to senescence; cluster 3 covers stress-responsive NAC proteins, and cluster 4 includes NAC proteins involved in fruit ripening but also in stress response and growth and development. The six strawberry FvNAC proteins are included in some of these clusters as shown below. FvNAC022 is clustered in group 1a involved in secondary cell wall development. One of the closer proteins was NAC73_ARATH (SND2, AT4G28500), as in the previous alignment done with Arabidopsis NAC proteins (S1 Fig). SND2 regulates genes involved in cellulose, mannan and xylan biosynthesis, cell wall modifications, and lignin polymerization. Also, it regulates the signal transduction pathway implied in the secondary cell wall development of Arabidopsis fibers [69]. FvNAC042 is included in cluster 1b which contains proteins related to growth and development and is near to NAC83_ARATH (VNI2) that negatively regulates xylem vessel formation [70]. VNI2 also interact with VND7 (VASCULAR-RELATED NAC-DOMAIN 7), a master regulator of xylem vessel differentiation [70]. Thus, FvNAC022 and FvNAC042 could have an important contribution in the regulation of the process that alters fruit texture during fruit ripening. Also, FvNAC022 was very close to A0A059SWK2_HEVBR (HbNAC1), which is involved in dehydration-induced laticifer differentiation in *Hevea brasiliensis*. The overexpression of *HbNAC1* enhances drought tolerance [71]. In accordance, only *FaNAC022* expression was seen when the strawberry fruits were submitted to drought (Fig 8). Thus, FvNAC022 could also be involved in stress response. Both FvNAC021 and Fv0NAC35 are included in the fruit ripening cluster (cluster 4) but FvNAC035 was more like the ripening related proteins. FaNAC035 was the only strawberry NAC gene specifically expressed in fruit (Fig 5). Fv0NAC35 was near to M5WG30_PRUPE (PpNAC1). PpNAC1 is a TF expressed in the late developmental stages of peach fruit and is involved in anthocyanin biosynthesis [23]. PpNAC1 forms a heterodimer with another NAC TF (BL), and activates the transcription of PpMYB10, leading to the anthocyanin pigmentation in tobacco [23]. Tomato NAC TFs with important roles in ethylene biosynthesis, reception and signaling of fruit ripening were found in cluster 4. Thus, FvNAC021 and Fv0NAC35 could be regulating important specific aspects of strawberry fruit ripening. FvNAC006 and FvNAC092 were included in cluster 1a with more heterogeneous functions, although most of the proteins were related to senescence. FvNAC006 is near to D7L212_ARALL (AtANAC046), which is a positive regulator of Arabidopsis leaf senescence controlling the expression of chlorophyll catabolic and senescence-associated genes [72]. Recently, it has reported that manipulation of the senescence-associated NAC gene *SlORE1S02* (UniProtKB—K4BBX4) improves tomato fruit yield [73]. Silenced transgenic plants showed delayed senescence and enhanced carbon assimilation that, in turn, increased the number of fruits. Moreover, fruits enhanced also their total soluble solid content and their nutraceutical composition [73]. K4BBX4_SOLLC is very close to FvNAc092 but, it was also near to U5I025_ROSHC (RhNAC100) that has been found to act as a negative regulator of cell expansion in rose petals [74]. FvNAC092 and RhNAC100 showed the highest identity in the alignment of all NAC proteins (90% and 94% of identity and homology, respectively, S10 Table). The expression studies of tissue specificity of the selected strawberry ripening-related *NAC* genes showed that *FaNAC092* was also highly expressed in petal in addition to those in receptacles at the senescent SE stage (Fig 5). This suggests that FvNAC092 could be involved in regulating cell expansion of strawberry petals, and in the fruit at the late stages of ripening. It has reported that cell expansion continued in receptacles for at least 28 d after anthesis [75]. The identity data for the six strawberry NAC proteins sequences when were compared to those of the closest related homologs in the cladogram are shown in S12 Table.

## Conclusions

A total of 112 *FaNAC* genes have been found in the current version of the annotated strawberry genome. The genomic features and the physiochemical properties of the corresponding proteins are shown. Six of these genes that are related to the development and ripening of the strawberry were selected after a microarray analysis that included all the genes present in the current *Fragaria* genome. Their expression patterns during this process and under different hormonal and stress conditions by qRT-PCR were studied. From these results and by means of the clustering of these proteins along Arabidopsis and many other plant NAC protein sequences with known functions, we can infer putative functions to the six development and ripening strawberry FaNAC proteins: FaNAC022and FaNAC042 could be involved in the regulation of the growth and development of the fruit, mainly related to the vascular tissue and in secondary cell wall development, and hence into fruit texture and firmness. FaNAC006 and FaNAC092 are likely to be regulating process related to fruit senescence, although both could also be involved in fruit development; and FaNAC021 and, especially FaNAC035 could be implied in specific process during the ripening of the fruit. Our study allows to determine these *FaNAC* genes as good candidates for further studies to establish their specific function in the development and ripening of the strawberry fruit and their potential utilization to improve strawberry quality by biotechnical approaches.

## Supporting information

S1 Table Fragaria NAC sequences discarded after a domain analysis done with MEME and Pfam program. Sequences eliminated from the *Fragaria* NAC protein collection. The name of the eliminated sequence is the name given to the gene found in the original strawberry genome public database (Fragaria vesca Genome v4.0.a1).
(DOCX)

S2 Table Analysis of discarded sequences for Pfam matches. Table shows the aligned sequences that were discarded because they were lacking and/or showed low identity in the NAC/NAM domain.
(DOCX)

S3 Table NAC proteins with known functions with indication of the plant species, gene names, the Uniprot, Genbank and Arabidopsis TAIR accessions, and the list of publications. (XLSX)

S4 Table Amino acid sequences in fasta format of NAC proteins of known functions. (DOCX)

S5 Table Primers used for the qRT-PCR quantitation of the six NAC genes related to the development and ripening of *Fragaria* x *ananassa*. (DOCX)

S6 Table Amino acid sequences in fasta format of strawberry NAC proteins. (DOCX)

S7 Table Physicochemical features and denomination of the Fragaria NAC family. (XLSX)

S8 Table Amino acid consensus sequences found in the motifs discovered by the MEME program. (DOCX)

S9 Table Putative membrane-bound domains present in strawberry NACs. (XLSX)

S10 Table Microarray data from transcriptomic comparison between red and green fruits for FvNAC genes. Magnitudes of relative induction to ripe receptacles (fold change), adjusted p.value (≤0.01) and the total signal intensity for each feature on microarrays platform in arbitrary units of intensity (a.u.i.) are given. Gene ID as reported in *Fragaria vesca* Genome Database (http://www.strawberrygenome.org/).
(XLSX)

S11 Table ABA content in fruit. Data is extracted from HPLC-MS quantitative analysis published elsewhere [5].
(XLSX)

S12 Table Identity data for the six strawberry NAC proteins sequences compared to those of the closest related homologs in the cladogram by using Needleman-Wunsch alignment (https://blast.ncbi.nlm.nih.gov/Blast.cgi). (XLSX)

S1 Fig Clustal Omega phylogenetic alignment of whole Arabidopsis NAC proteins along the ripening-related strawberry NAC proteins. Red: *Fragaria vesca* sequences; Blue: Arabidopsis genes related to the biosynthesis and/or development of vascular tissue and cell-walls; Green: Arabidopsis genes related to water balance and/or stress. Biological functions are defined at the beginning or the end of the protein name: S: senescence; D: defense; M: Meristem formation; Fv: Flavonoid biosynthesis; H: Response to hydrogen peroxide; R: Root cap; C: Cell division; Ce: Cell size; Pol: Pollen development; A: ABA response; F: Flowering; E: Epistasis; P: Proline; RF: Flowering repression; V: biosynthesis and/or development of vascular tissue and cell-walls; Dh: Fruit Dehiscence; An: Anthocyanin related; Wb: water balance and/or stress; L: Leaf formation; Em: Embryo development; G: DNA damage. In S4 and S7 Tables, we have included live URL links that provides the correspondence between Arabidopsis protein names and their corresponding UniProt accession.
(TIF)

S2 Fig Clustal Omega phylogenetic alignment of the N-terminal regions of the Arabidopsis NAC proteins along the ripening-related strawberry NAC proteins including the NAC subdomains. Red: *Fragaria vesca* sequences; Blue: Arabidopsis genes related to the biosynthesis and/or development of vascular tissue and cell-walls; Green: Arabidopsis genes related to water balance and/or stress. Biological functions are defined at the beginning or the end of the protein name: S: senescence; D: defense; M: Meristem formation; Fv: Flavonoid biosynthesis; H: Response to hydrogen peroxide; R: Root cap; C: Cell division; Ce: Cell size; Pol: Pollen development; A: ABA response; F: Flowering; E: Epistasis; P: Proline; RF: Flowering repression; V: biosynthesis and/or development of vascular tissue and cell-walls; Dh: Fruit Dehiscence; An: Anthocyanin related; W: water balance and/or stress; L: Leaf formation; Em: Embryo development; G: DNA damage. In S4 and S7 Tables, we have included live URL links that provides the correspondence between Arabidopsis protein names and their corresponding UniProt accession.
(TIF)

S3 Fig Clustal Omega phylogenetic alignment of the C-terminal regions of the Arabidopsis NAC proteins along the ripening-related strawberry NAC proteins excluding the NAC subdomains. Alignment of only the C-terminal portion of the NAC proteins containing the TR (transcriptional regulatory). Red: *Fragaria vesca* sequences; Blue: Arabidopsis genes related to the biosynthesis and/or development of vascular tissue and cell-walls; Green: Arabidopsis genes related to water balance and/or stress. Biological functions are defined at the beginning or the end of the protein name: S: senescence; D: defense; M: Meristem formation; Fv: Flavonoid biosynthesis; H: Response to hydrogen peroxide; R: Root cap; C: Cell division; Ce: Cell size; Pol: Pollen development; A: ABA response; F: Flowering; E: Epistasis; P: Proline; RF: Flowering repression; V: biosynthesis and/or development of vascular tissue and cell-walls; Dh: Fruit Dehiscence; An: Anthocyanin related; W: water balance and/or stress; L: Leaf formation; Em: Embryo development; G: DNA damage. In S4 and S7 Tables, we have included live URL links that provides the correspondence between Arabidopsis protein names and their corresponding UniProt accession.
(TIF)
