# Supplementary material for: Genome-wide analysis of the NAC transcription factor family and their expression during the development and ripening of the Fragaria × ananassa fruits
Source: PLoS One. 2018 May 3;13(5):e0196953. doi: 10.1371/journal.pone.0196953 (PMC5933797; doi:10.1371/journal.pone.0196953)
Supplement: S1 Table — Sequences eliminated from the Fragaria NAC protein collection. The name of the eliminated sequence is the name given to the gene found in the original strawberry genome public database (Fragaria vesca Genome v4.0.a1). (DOCX) [file pone.0196953.s001.docx]

| Discarded Sequence | Reason |
| --- | --- |
| FvH4_3g03550.1 | It has 47 amino acids only, substantially lower than the NAC/NAM domain itself. It lacks the TRR or C-terminal portion of the protein |
| FvH4_3g16030.1 | It has 45 amino acids only, substantially lower than the NAC/NAM domain itself. It lacks the TRR or C-terminal portion of the protein |
| FvH4_6g24230.1 | It has 71 amino acids only and low identity in the NAC/NAM domain. |
| FvH4_7g01360.1 | Lacking whole NAC/NAM domain |
| FvH4_7g01350.1 | Lacking whole NAC/NAM domain |

| **NAME** | **Pfam** | | **MEME** |
| --- | --- | --- | --- |
|  | E-value | lenght | E-value |
| FvH4_3g03550.1 | 1.1e-13 | 47aa | 9.77e-22 |
| FvH4_3g16030.1 | 4.7e-12 | 45aa | 6.74e-22 |
| FvH4_6g24230.1 | 2.5e-12 | 71aa | 1.00e-15 |
| FvH4_7g01360.1 | Lack whole domain | | 7.14e-7 |
| FvH4_7g01350.1 | Lack whole domain | | ND |

**S1 Table. *Fragaria* NAC sequences discarded after a domain analysis done with MEME and Pfam programs.** Sequences eliminated from the *Fragaria* NAC protein collection. The name of the eliminated sequence is the name given to the gene found in the original strawberry genome public database (*Fragaria vesca* Genome v4.0.a1).
