# Supplementary material for: Genome-wide analysis of the NAC transcription factor family and their expression during the development and ripening of the Fragaria × ananassa fruits
Source: PLoS One. 2018 May 3;13(5):e0196953. doi: 10.1371/journal.pone.0196953 (PMC5933797; doi:10.1371/journal.pone.0196953)
Supplement: S2 Table — Table shows the aligned sequences that were discarded because they were lacking and/or showed low identity in the NAC/NAM domain. (DOCX) [file pone.0196953.s002.docx]

**FvH4_3g03550.1**

**
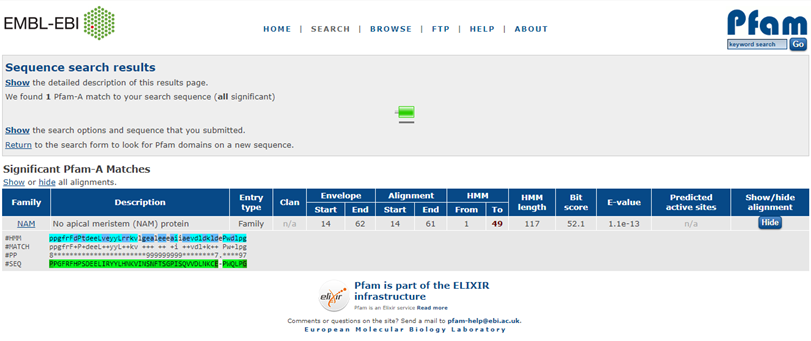
**

**FvH4_3g16030.1**

**
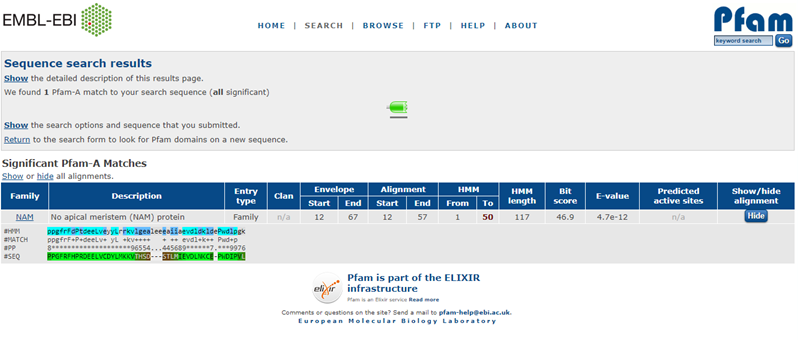
**

**FvH4_6g24230.1**

**
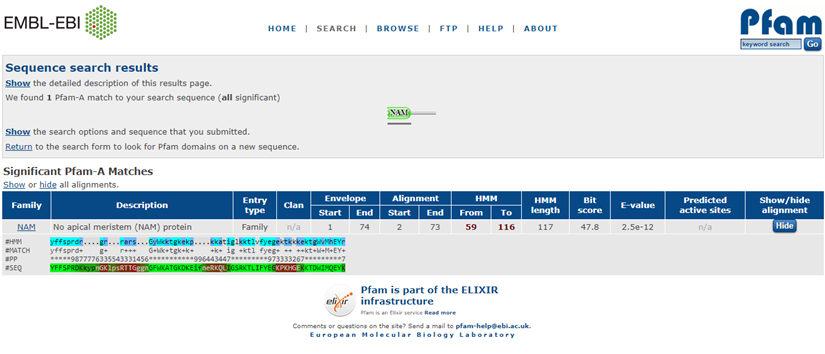
**

**FvH4_7g01360.1**

**
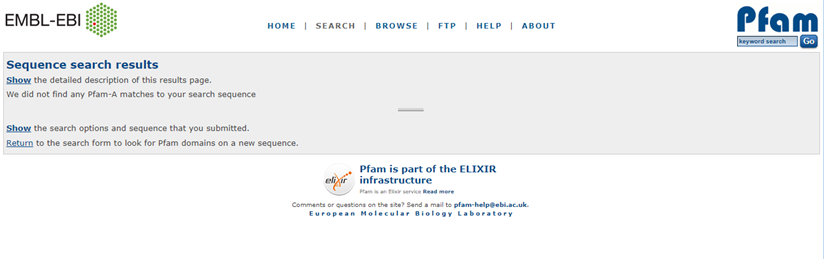
**

**FvH4_7g01350.1**

**
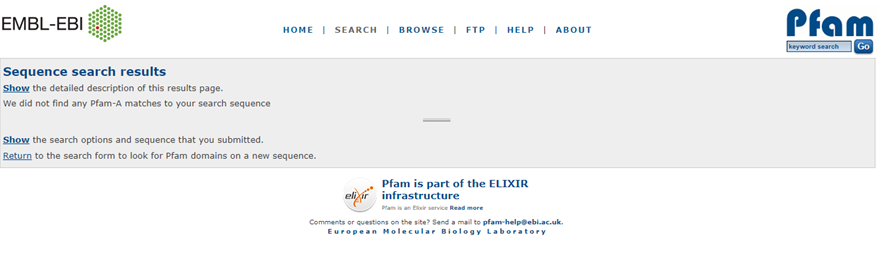
**

**S2 Table. Analysis of discarded sequences for Pfam matches.** Table shows the aligned sequences that were discarded because they were lacking and/or showed low identity in the NAC/NAM domain.
