# Supplementary material for: Genome-wide analysis of the NAC transcription factor family and their expression during the development and ripening of the Fragaria × ananassa fruits
Source: PLoS One. 2018 May 3;13(5):e0196953. doi: 10.1371/journal.pone.0196953 (PMC5933797; doi:10.1371/journal.pone.0196953)
Supplement: S4 Table — (DOCX) [file pone.0196953.s004.docx]

**S4 Table. Amino acid sequences in fasta format of NAC proteins of known functions.**

>Q8H0I5_ORYSA Growth & Develop OsNAC1 protein OS=Oryza sativa GN=OsNAC1 PE=2 SV=1

MDLPPGFRFHPTDEELITHYLLRKAADPAGFAARAVGEADLNKCEPWDLPSRATMGEKEW

YFFCVKDRKYPTGLRTNRATESGYWKATGKDREIFRGKALVGMKKTLVFYTGRAPRGGKT

GWVMHEYRIHGKHAAANSKQDQEWVLCRVFKKSLELAPAAAAAVGRRGAGAGTDVGPSSM

PMADDVVGLAPCALPPLMDVSGGGGGAGTTSLSATAGAAAAPPAAHVTCFSNALEGQFLD

TPYLLPAADPADHLAMSSASPFLEALQMQYVQDAAAAGGAGMVHELLMGGGWYCNKGERE

RLSGASQDTGLTSSEVNPGEISSSSRHNAWIITTRPSGPIEIFIHHH

>Q8H0I4_ORYSA Growth & Development OsNAC2 protein OS=Oryza sativa GN=OsNAC2 PE=2 SV=1

MDLPPGFRFHPTDEELITHYLAKKVADARFAALAVAEADLNKCEPWDLPSLAKMGEKEWY

FFCLKDRKYPTGLRTNRATESGYWKATGKDKDIFRRKALVGMKKTLVFYTGRAPKGEKSG

WVMHEYRLHGKLHAAALGFLHGKPASSKNEWVLCRVFKKSLVEVGAAGGKKAAVVTMEMA

RGGSTSSSVADEIAMSSVVLPPLMDMSGAGAGAVDPATTAHVTCFSNALEGQFFNPTAVH

GHGGGDSSPFMASFTQYGQLHHGVSLVQLLESCNGYGGLVDMAASGSQLQPAACGGERER

LSASQDTGLTSDVNPEISSSSGQKFDHEAALWGY

>NAC48_ORYSJ Stress NAC domain-containing protein 48 OS=Oryza sativa subsp. japonica GN=NAC48 PE=2 SV=1

MSGGQDLQLPPGFRFHPTDEELVMHYLCRRCAGLPIAVPIIAEIDLYKFDPWQLPRMALY

GEKEWYFFSPRDRKYPNGSRPNRAAGSGYWKATGADKPVGSPKPVAIKKALVFYAGKAPK

GEKTNWIMHEYRLADVDRSARKKNSLRLDDWVLCRIYNKKGGLEKPPAAAVAAAGMVSSG

GGVQRKPMVGVNAAVSSPPEQKPVVAGPAFPDLAAYYDRPSDSMPRLHADSSCSEQVLSP

EFACEVQSQPKISEWERTFATVGPINPAASILDPAGSGGLGGLGGGGSDPLLQDILMYWG

KPF

>Q6RH27_SOLLC Stress Organ identity Fruit pigmentation ripening NAC domain protein OS=Solanum lycopersicum GN=NAC1 PE=2 SV=1

MNKGANGNQQLELPAGFRFHPTDDELVQHYLCRKCAGQSIAVSIIAEIDLYKFDPWQLPE

KALYGEKEWYFFSPRDRKYPNGSRPNRAAGTGYWKATGADKPVGKPKTLGIKKALVFYAG

KAPRGIKTNWIMHEYRLANVDRSAGKNNNLRLDDWVLCRIYNKKGTLEKHYNVDNKETTS

FGEFDEEIKPKILPTQLAPMPPRPRSTPANDYFYFESSESMTRMHTTNSSSGSEHVLSPC

DKEVQSAPKWDEDHRNTLDFQLNYLDGLLNEPFETQMQQQICNFDQFNNFQDMFLYMQKP

Y

>B8XS01_SOLLC Goblet OS=Solanum lycopersicum GN=NAM PE=2 SV=1

MEIYHQMQFDCGDPHLPPGFRFHPTDEELITYYLLKKVLDCNFTARAIAEVDLNKCEPWE

LPGKAKMGEKEWYFFSLRDRKYPTGLRTNRATEAGYWKATGKDREIFSSKTCALVGMKKT

LVFYRGRAPKGEKSNWVMHEYRLDGKFAYHYISRSSKDEWVISRVFQKSTGSNGAATSTG

GGKKRLSSSINMYQEVSSPSSVSHLPPLLDSSPYSTTATSAAAIVIGDRDRDHSFKKEHV

PCFSTTATATITAQSLTFDPTSVFDISSNTLHALQPTPSFASILDSSPSNFTNYTRNSTF

PSLRSLHENLQLPLFSGGTSAMHGGFSNPMVNWTVPETQKVEQSELDCMWSY

>K4BWV2_SOLLC Uncharacterized protein OS=Solanum lycopersicum GN=101244582 PE=4 SV=1

MVGKISSDLPPGFRFHPTDEELIMYYLRYQATSRPCPVSIIPEIDVYKFDPWVLPEKAEF

GDNEWYFFTPRDRKYPNGVRPNRAAVSGYWKATGTDKAIYSANKYVGIKKALVFYKGKPP

KGVKTDWIMHEYRLSDSKSQTSKQSGSMRLDDWVLCRIYKKKNLGRTIEMMKVEEEELEA

QNVSTTNNEIEVVGGPQTMKLPRICSLSHLLELDYFGSIPQLLSDNLLYDDQGYTMNNVN

NTSNVDQVSSQQQNTNNITSNNCNIFFNYQQPLFVNPTFQSQ

>K4D6Q0_SOLLC Ripening NAC4 domain protein OS=Solanum lycopersicum GN=101267475 PE=2 SV=1

MVELQFPAGFRFHPTDEELVMHYLCRKCDAQPIAVPIIAEIDLYKYNPWDLPDLALYGEK

EWYFFSPRDRKYPNGSRPNRAAGSGYWKATGADKPIGRPKSMGIKKALVFYAGKAPKGEK

TNWIMHEYRLAHVDRSARNKNNSLRLDDWVLCRIYNKKGTIEKNQLNIRKMNGEMSPAVS

EGDVKPEIVPISVSTNPSSTSYHVYNDFTYFNSPDSLTKLHADSSCSEHVPSPEFTYEKE

VQSEPKPKPSEWEKTALDFPFNYTDATASELQSCYEMSPLQDIFMYLQKPF

>Q52QR4_SOYBN Stress NAC domain protein NAC2 OS=Glycine max GN=NAC2 PE=2 SV=1

MASELELPPGFRFHPTDEELVLHYLCRKCASQPIAVPIIAEIDLYKYDPWDLPGLATYGE

KEWYFFSPRDRKYPNGSRPNRAAGTGYWKATGADKPIGQPKPVGIKKALVFYAGKAPKGD

KSNWIMHEYRLADVDRSVRKKNTLRLDDWVLCRIYNKKGTIEKLQPSSDVAHSRNIESSE

IEDRKPEILKSGGGCLPPPAPVPAPPQATAKTDYMYFDPSDSIPKLHTDSSCSEQVVSPG

FASEVQSEPKWNEWEKSLEFPFNYVDATLNNSFMAQFQGNNQMLSPLQDMFMYWPNKSF

>Q3ZN85_CAPAN CaNAC1 Stress NAC domain protein 1 OS=Capsicum annuum PE=2 SV=1

MIKGIVGNQQLGLPAGFRFHPTDEELVQHYLCRKCAGQSISVSIIAEIDLYKFDPWQLPE

KALYGEKEWYFFSQEDRKYPNGSRPNRAAGTGYWKATGADKPVGKPKTLGIKKALVFYAG

KAPRGIKTNWIMHEYRLANVDRSAGKSNNLRLDDWVLCRIYNKKGHTLRSITMWASKRGE

SFGEFEDEIKPKIFPTQLAPAPGQWPPRPQSTPASDYFNFETSESMTTTRMHTTNSSSGS

EHVLSSCDKEVQSAPKWDDLGTAALDFQINYLDGLLNDPFEIQMQQQNCNIDQFNTFQDM

FLYMQKP

>Q948Z2_SOLTU StNAC Stress Putative NAC domain protein OS=Solanum tuberosum GN=nac PE=2 SV=1

MNKGATGNQQLELPAGFRFHPTDDELVQHYLCRKCAGQPIAVSIITEIDLYKFDPWQLPE

KALYGEKEWYFFSPRDRKYPNGSRPNRAAGTGYWKATGADKPVGKPKTLGIKKALVFYAG

KAPRGIKTNWIMHEYRLANVDRSAGKNNNLRLDDWVLCRIYNKKGTLEKHYNVDNKETAS

FGEFDEEIKPKILPTQLAQMPPRPRSTPTNDYFHFESSESMTRMHTTNSSSGSEHVLSPC

DKEVQSAPKWDEDHRNTLDFQLNYLDGLLNEPFETQMQQQSCNFDQFNNFQDMFFYMQKP

Y

>A2IB55_CITSI NAC domain protein OS=Citrus sinensis GN=NAC PE=2 SV=1

MEAQASTELPPGFRFHPTDEELIVHYLRNQATSRPCPVSIIPEVDIYKFDPWQLPEKAEF

GEKEWYFFSPRDRKYPNGTRPNRATVSGYWKATGTDKAIYGGSKYLGVKKALVFYKGRPP

KGIKTDWIMHEYRLNDPTRQPYKHNGSMKLDDWVLCRIYKKRQTGSRSVLDAKVEEDQSC

VDQLGKTGGYVEHANASDEQKLMVKFPRTCSLAHLVELEYFAPISQLLNDNTYNFNYDFQ

NGINNNAASDDQFEINFSQVTCTK

>Q40880_PETHY NAM protein OS=Petunia hybrida GN=NAM PE=2 SV=1

MENYQHFDCSDSNLPPGFRFHPTDEELITYYLLKKVLDSNFTGRAIAEVDLNKCEPWELP

EKAKMGEKEWYFFSLRDRKYPTGLRTNRATEAGYWKATGKDREIYSSKTSALVGMKKTLV

FYRGRAPKGEKSNWVMHEYRLDGKFAYHYISRSSKDEWVISRVFQKSCSTVGTTSNGGKK

RLNSSFNNMYQEVSSPSSVSLPPLLESSPYNNTATSAAASKKEHVSCFSTISTPSFDPSS

VFDISSNSNTLHSLPAPSFSAILDPSSTFSRNSVFPSLRSLQENLHLPLFSGGTSAMHGG

FSSPLANWPVPETQKVDHSELDCMWSY

>Q93XA6_PHAVU Senescence NAC domain protein NAC2 OS=Phaseolus vulgaris GN=PHAVU_002G170200g PE=2 SV=1

MDATTPSELPPGFRFHPTDEELIVYYLCNQATSKPCPASIIPEVDLYKFDPWELPDKTEF

GENEWYFFSPRDRKYPNGVRPNRATVSGYWKATGTDKAIYSGSKLVGVKKSLVFYKGRPP

KGDKTDWIMHEYRLAESKQPVNRKIGSMRLDDWVLCRIYKKKNTGKTLEHKETHPKVQMT

NLIAANNDEQKMMNLPRTWSLTYLLDMNYLGPILSDGSYCSTFDFQISNANIGIDPFVNS

QPVEMANNYVSDSGKY

>Q2HIR8_ARATH Stress At1g01720 OS=Arabidopsis thaliana PE=2 SV=1

MSELLQLPPGFRFHPTDEELVMHYLCRKCASQSIAVPIIAEIDLYKYDPWELPGLALYGE

KEWYFFSPRDRKYPNGSRPNRSAGSGYWKATGADKPIGLPKPVGIKKALVFYAGKAPKGE

KTNWIMHEYRLADVDRSVRKKKNSLRLDDWVLCRIYNKKGATERRGPPPPVVYGDEIMEE

KPKVTEMVMPPPPQQTSEFAYFDTSDSVPKLHTTDSSCSEQVVSPEFTSEVQSEPKWKDW

SAVSNDNNNTLDFGFNYIDATVDNAFGGGGSSNQMFPLQDMFMYMQKPY

>NAC81_ARATH Stress Protein ATAF2 OS=Arabidopsis thaliana GN=NAC081 PE=1 SV=1

MKSELNLPAGFRFHPTDEELVKFYLCRKCASEQISAPVIAEIDLYKFNPWELPEMSLYGE

KEWYFFSPRDRKYPNGSRPNRAAGTGYWKATGADKPIGKPKTLGIKKALVFYAGKAPKGI

KTNWIMHEYRLANVDRSASVNKKNNLRLDDWVLCRIYNKKGTMEKYFPADEKPRTTTMAE

QSSSPFDTSDSTYPTLQEDDSSSSGGHGHVVSPDVLEVQSEPKWGELEDALEAFDTSMFG

SSMELLQPDAFVPQFLYQSDYFTSFQDPPEQKPFLNWSFAPQG

>NAC54_ARATH Protein CUP-SHAPED COTYLEDON 1 OS=Arabidopsis thaliana GN=NAC054 PE=1 SV=1

MDVDVFNGWGRPRFEDESLMPPGFRFHPTDEELITYYLLKKVLDSNFSCAAISQVDLNKS

EPWELPEKAKMGEKEWYFFTLRDRKYPTGLRTNRATEAGYWKATGKDREIKSSKTKSLLG

MKKTLVFYKGRAPKGEKSCWVMHEYRLDGKFSYHYISSSAKDEWVLCKVCLKSGVVSRET

NLISSSSSSAVTGEFSSAGSAIAPIINTFATEHVSCFSNNSAAHTDASFHTFLPAPPPSL

PPRQPRHVGDGVAFGQFLDLGSSGQIDFDAAAAAFFPNLPSLPPTVLPPPPSFAMYGGGS

PAVSVWPFTL

>NAC98_ARATH Protein CUP-SHAPED COTYLEDON 2 OS=Arabidopsis thaliana GN=NAC098 PE=1 SV=1

MDIPYYHYDHGGDSQYLPPGFRFHPTDEELITHYLLRKVLDGCFSSRAIAEVDLNKCEPW

QLPGRAKMGEKEWYFFSLRDRKYPTGLRTNRATEAGYWKATGKDREIFSSKTCALVGMKK

TLVFYKGRAPKGEKSNWVMHEYRLEGKFSYHFISRSSKDEWVISRVFQKTTLASTGAVSE

GGGGGGATVSVSSGTGPSKKTKVPSTISRNYQEQPSSPSSVSLPPLLDPTTTLGYTDSSC

SYDSRSTNTTVTASAITEHVSCFSTVPTTTTALGLDVNSFSRLPPPLGFDFDPFPRFVSR

NVSTQSNFRSFQENFNQFPYFGSSSASTMTSAVNLPSFQGGGGVSGMNYWLPATAEENES

KVGVLHAGLDCIWNY

>NAC31_ARATH Protein CUP-SHAPED COTYLEDON 3 OS=Arabidopsis thaliana GN=NAC031 PE=1 SV=1

MMLAVEDVLSELAGEERNERGLPPGFRFHPTDEELITFYLASKIFHGGLSGIHISEVDLN

RCEPWELPEMAKMGEREWYFYSLRDRKYPTGLRTNRATTAGYWKATGKDKEVFSGGGGQL

VGMKKTLVFYKGRAPRGLKTKWVMHEYRLENDHSHRHTCKEEWVICRVFNKTGDRKNVGL

IHNQISYLHNHSLSTTHHHHHEALPLLIEPSNKTLTNFPSLLYDDPHQNYNNNNFLHGSS

GHNIDELKALINPVVSQLNGIIFPSGNNNNDEDDFDFNLGVKTEQSSNGNEIDVRDYLEN

PLFQEASYGLLGFSSSPGPLHMLLDSPCPLGFQL

>NAC56_ARATH NAC transcription factor 56 OS=Arabidopsis thaliana GN=NAC056 PE=2 SV=1

MESTDSSGGPPPPQPNLPPGFRFHPTDEELVVHYLKRKAASAPLPVAIIAEVDLYKFDPW

ELPAKASFGEQEWYFFSPRDRKYPNGARPNRAATSGYWKATGTDKPVLASDGNQKVGVKK

ALVFYSGKPPKGVKSDWIMHEYRLIENKPNNRPPGCDFGNKKNSLRLDDWVLCRIYKKNN

ASRHVDNDKDHDMIDYIFRKIPPSLSMAAASTGLHQHHHNVSRSMNFFPGKFSGGGYGIF

SDGGNTSIYDGGGMINNIGTDSVDHDNNADVVGLNHASSSGPMMMANLKRTLPVPYWPVA

DEEQDASPSKRFHGVGGGGGDCSNMSSSMMEETPPLMQQQGGVLGDGLFRTTSYQLPGLN

WYSS

>NAC43_ARATH Cell wall NAC domain-containing protein 43 OS=Arabidopsis thaliana GN=NAC043 PE=2 SV=2

MMSKSMSISVNGQSQVPPGFRFHPTEEELLQYYLRKKVNSIEIDLDVIRDVDLNKLEPWD

IQEMCKIGTTPQNDWYFFSHKDKKYPTGTRTNRATAAGFWKATGRDKIIYSNGRRIGMRK

TLVFYKGRAPHGQKSDWIMHEYRLDDNIISPEDVTVHEVVSIIGEASQDEGWVVCRIFKK

KNLHKTLNSPVGGASLSGGGDTPKTTSSQIFNEDTLDQFLELMGRSCKEELNLDPFMKLP

NLESPNSQAINNCHVSSPDTNHNIHVSNVVDTSFVTSWAALDRLVASQLNGPTSYSITAV

NESHVGHDHLALPSVRSPYPSLNRSASYHAGLTQEYTPEMELWNTTTSSLSSSPGPFCHV

SNGSG

>NAC66_ARATH Cell wall NAC domain-containing protein 66 OS=Arabidopsis thaliana GN=NAC066 PE=2 SV=1

MNISVNGQSQVPPGFRFHPTEEELLKYYLRKKISNIKIDLDVIPDIDLNKLEPWDIQEMC

KIGTTPQNDWYFYSHKDKKYPTGTRTNRATTVGFWKATGRDKTIYTNGDRIGMRKTLVFY

KGRAPHGQKSDWIMHEYRLDESVLISSCGDHDVNVETCDVIGSDEGWVVCRVFKKNNLCK

NMISSSPASSVKTPSFNEETIEQLLEVMGQSCKGEIVLDPFLKLPNLECHNNTTITSYQW

LIDDQVNNCHVSKVMDPSFITSWAALDRLVASQLNGPNSYSIPAVNETSQSPYHGLNRSG

CNTGLTPDYYIPEIDLWNEADFARTTCHLLNGSG

>NAC12_ARATH Cell wall NAC domain-containing protein 12 OS=Arabidopsis thaliana GN=NAC012 PE=2 SV=1

MADNKVNLSINGQSKVPPGFRFHPTEEELLHYYLRKKVNSQKIDLDVIREVDLNKLEPWD

IQEECRIGSTPQNDWYFFSHKDKKYPTGTRTNRATVAGFWKATGRDKIICSCVRRIGLRK

TLVFYKGRAPHGQKSDWIMHEYRLDDTPMSNGYADVVTEDPMSYNEEGWVVCRVFRKKNY

QKIDDCPKITLSSLPDDTEEEKGPTFHNTQNVTGLDHVLLYMDRTGSNICMPESQTTTQH

QDDVLFMQLPSLETPKSESPVDQSFLTPSKLDFSPVQEKITERPVCSNWASLDRLVAWQL

NNGHHNPCHRKSFDEEEENGDTMMQRWDLHWNNDDNVDLWSSFTESSSSLDPLLHLSV

>NAC29_ARATH Growth & development / embryogenesis NAC transcription factor 29 OS=Arabidopsis thaliana GN=NAC029 PE=2 SV=1

MEVTSQSTLPPGFRFHPTDEELIVYYLRNQTMSKPCPVSIIPEVDIYKFDPWQLPEKTEF

GENEWYFFSPRERKYPNGVRPNRAAVSGYWKATGTDKAIHSGSSNVGVKKALVFYKGRPP

KGIKTDWIMHEYRLHDSRKASTKRNGSMRLDEWVLCRIYKKRGASKLLNEQEGFMDEVLM

EDETKVVVNEAERRTEEEIMMMTSMKLPRTCSLAHLLEMDYMGPVSHIDNFSQFDHLHQP

DSESSWFGDLQFNQDEILNHHRQAMFKF

>NAC91_ARATH NAC domain-containing protein 91 OS=Arabidopsis thaliana GN=NAC091 PE=1 SV=1

MKEDMEVLSLASLPVGFRFSPTDEELVRYYLRLKINGHDNDVRVIREIDICKWEPWDLPD

FSVVKTTDSEWLFFCPLDRKYPSGSRMNRATVAGYWKATGKDRKIKSGKTKIIGVKRTLV

FYTGRAPKGTRTCWIMHEYRATEKDLDGTKSGQNPFVVCKLFKKQDIVNGAAEPEESKSC

EVEPAVSSPTVVDEVEMSEVSPVFPKTEETNPCDVAESSLVIPSECRSGYSVPEVTTTGL

DDIDWLSFMEFDSPKLFSPLHSQVQSELGSSFNGLQSESSELFKNHNEDYIQTQYGTNDA

DEYMSKFLDSFLDIPYEPEQIPYEPQNLSSCNKINDESKRGIKIRARRAQAPGCAEQFVM

QGDASRRLRLQVNLNSHKSETDSTQLQFIKKEVKDTTTETMTKGCGNFTRSKSRTSFIFK

KIAAMGCSYRGLFRVGVVAVVCVMSVCSLVA

>NAC18_ARATH NAC domain-containing protein 18 OS=Arabidopsis thaliana GN=NAC018 PE=2 SV=1

MESTDSSGGPPPPQPNLPPGFRFHPTDEELVIHYLKRKADSVPLPVAIIADVDLYKFDPW

ELPAKASFGEQEWYFFSPRDRKYPNGARPNRAATSGYWKATGTDKPVISTGGGGSKKVGV

KKALVFYSGKPPKGVKSDWIMHEYRLTDNKPTHICDFGNKKNSLRLDDWVLCRIYKKNNS

TASRHHHHLHHIHLDNDHHRHDMMIDDDRFRHVPPGLHFPAIFSDNNDPTAIYDGGGGGY

GGGSYSMNHCFASGSKQEQLFPPVMMMTSLNQDSGIGSSSSPSKRFNGGGVGDCSTSMAA

TPLMQNQGGIYQLPGLNWYS

>NAC82_ARATH Cell wall metabolism NAC domain-containing protein 82 OS=Arabidopsis thaliana GN=NAC082 PE=1 SV=1

MGKTQLAPGFRFHPTDVELVRYYLKRKILGKKLLVDAIAEVDIYKFEPPDLPDMSFIRSG

DLKWHFFCPREKKYASGVRANRATECGYWKTTGKERPVLCNSEVVGKIKTLVYHFGKSPR

GERTDWVMHEYRLDDKVLTQMNVPQDTYVVCVLFKKDGPGPRNGAQYGAPFKEEDWSDEE

VRTDVPSTSNPTNLLEPSKETTLALTAPDDSNKDCFGGMISESCVSDFLPATTNTTSELP

HPSDAATTPMSTAPLAETVQTPNNDDLYSMLDLFDDDEEFLGFNNNEVRYDPGVSAPVCL

EEEGIFNGLPELSSMPRTASYDLVENSELYLELQDLTAPLNPQTGLQDLTAPFNPQTGLQ

DLTAPFNHQTGLQDHTAPFNPQTGLQDHTAPFNHQTGLQDLTAPFNPQTGLQDLTAPFNP

QTGLHDLTSPFNPQTGLQDLTAPLNPQTGNRNDPRSSSFLYNQGHFDFSGGNDDDPYGFS

ASMRHRPKM

>NAC83_ARATH Cell wall NAC domain-containing protein 83 OS=Arabidopsis thaliana GN=NAC083 PE=1 SV=1

MDNVKLVKNGVLRLPPGFRFHPTDEELVVQYLKRKVCSSPLPASIIPEFDVCRADPWDLP

GNLEKERYFFSTREAKYPNGNRSNRATGSGYWKATGIDKRVVTSRGNQIVGLKKTLVFYK

GKPPHGSRTDWIMHEYRLSSSPPSSMGPTQNWVLCRIFLKKRAGNKNDDDDGDSRNLRHN

NNNNSSDQIEIITTDQTDDKTKPIFFDFMRKERTTDLNLLPSSPSSDHASSGVTTEIFSS

SDEETSSCNSFR

>FEZ_ARATH Growth & Development Protein FEZ OS=Arabidopsis thaliana GN=FEZ PE=2 SV=1

MGDRNNDGDQKMEDVLLPGFRFHPTDEELVSFYLKRKVQHNPLSIELIRQLDIYKYDPWD

LPKFAMTGEKEWYFYCPRDRKYRNSSRPNRVTGAGFWKATGTDRPIYSSEGNKCIGLKKS

LVFYKGRAAKGVKTDWMMHEFRLPSLSEPSPPSKRFFDSPVSPNDSWAICRIFKKTNTTT

LRALSHSFVSSLPPETSTDTMSNQKQSNTYHFSSDKILKPSSHFQFHHENMNTPKTSNST

TPSVPTISPFSYLDFTSYDKPTNVFNPVSCLDQQYLTNLFLATQETQPQFPRLPSSNEIP

SFLLNTSSDSTFLGEFTSHIDLSAVLAQEQCPPLVSLPQEYQETGFEGNGIMKNMRGSNE

DHLGDHCDTLRFDDFTSTINENHRHHQDLKQNMTLLESYYSSLSSINSDLPACFSSTT

>BRN1_ARATH Protein BEARSKIN1 OS=Arabidopsis thaliana GN=BRN1 PE=2 SV=1

MSSSNGGVPPGFRFHPTDEELLHYYLKKKISYEKFEMEVIKEVDLNKIEPWDLQDRCKIG

STPQNEWYFFSHKDRKYPTGSRTNRATHSGFWKATGRDKCIRNSYKKIGMRKTLVFYKGR

APHGQKTDWIMHEYRIEDTEDDPCEDGWVVCRVFKKKNLFKVGNDVGSNISNNRLEARSF

IRRESPYQGISMFELNKPEEISVHQYPQPPMFQPHHKPLSIGYDYSLALLPRESEYQQAC

QPSGVEVGTCKAVSEWGIVNCNMVSHEDSSRAMRFEDDGNNTSSTVQPPSNLLSLRGENG

FLGLF

>BRN2_ARATH Protein BEARSKIN2 OS=Arabidopsis thaliana GN=BRN2 PE=2 SV=1

MGSSSNGGVPPGFRFHPTDEELLHYYLKKKISYQKFEMEVIREVDLNKLEPWDLQERCKI

GSTPQNEWYFFSHKDRKYPTGSRTNRATHAGFWKATGRDKCIRNSYKKIGMRKTLVFYKG

RAPHGQKTDWIMHEYRLEDADDPQANPSEDGWVVCRVFMKKNLFKVVNEGSSSINSLDQH

NHDASNNNHALQARSFMHRDSPYQLVRNHGAMTFELNKPDLALHQYPPIFHKPPSLGFDY

SSGLARDSESAASEGLQYQQACEPGLDVGTCETVASHNHQQGLGEWAMMDRLVTCHMGNE

DSSRGITYEDGNNNSSSVVQPVPATNQLTLRSEMDFWGYSK

>SMB_ARATH Protein SOMBRERO OS=Arabidopsis thaliana GN=SMB PE=1 SV=1

MEIGSSSTVAGGGQLSVPPGFRFHPTEEELLYYYLKKKVSYEPIDLDVIREVDLNKLEPW

ELKEKCRIGSGPQNEWYFFSHKDKKYPTGTRTNRATAAGFWKATGRDKSIHLNSSKKIGL

RKTLVFYTGRAPHGQKTEWIMHEYRLDDSENEIQEDGWVVCRVFKKKNHFRGFHQEQEQD

HHHHHQYISTNNDHDHHHHIDSNSNNHSPLILHPLDHHHHHHHIGRQIHMPLHEFANTLS

HGSMHLPQLFSPDSAAAAAAAAASAQPFVSPINTTDIECSQNLLRLTSNNNYGGDWSFLD

KLLTTGNMNQQQQQQVQNHQAKCFGDLSNNDNNDQADHLGNNNGGSSSSPVNQRFPFHYL

GNDANLLKFPK

>NAC92_ARATH NAC domain-containing protein 92 OS=Arabidopsis thaliana GN=NAC92 PE=1 SV=1

MDYEASRIVEMVEDEEHIDLPPGFRFHPTDEELITHYLKPKVFNTFFSATAIGEVDLNKI

EPWDLPWKAKMGEKEWYFFCVRDRKYPTGLRTNRATEAGYWKATGKDKEIFKGKSLVGMK

KTLVFYKGRAPKGVKTNWVMHEYRLEGKYCIENLPQTAKNEWVICRVFQKRADGTKVPMS

MLDPHINRMEPAGLPSLMDCSQRDSFTGSSSHVTCFSDQETEDKRLVHESKDGFGSLFYS

DPLFLQDNYSLMKLLLDGQETQFSGKPFDGRDSSGTEELDCVWNF

>NAC59_ARATH NAC domain-containing protein 59 OS=Arabidopsis thaliana GN=NAC59 PE=1 SV=1

MDYKVSRSGEIVEGEVEDSEKIDLPPGFRFHPTDEELITHYLRPKVVNSFFSAIAIGEVD

LNKVEPWDLPWKAKLGEKEWYFFCVRDRKYPTGLRTNRATKAGYWKATGKDKEIFKGKSL

VGMKKTLVFYKGRAPKGVKTNWVMHEYRLEGKFAIDNLSKTAKNECVISRVFHTRTDGTK

EHMSVGLPPLMDSSPYLKSRGQDSLAGTTLGGLLSHVTYFSDQTTDDKSLVADFKTTMFG

SGSTNFLPNIGSLLDFDPLFLQNNSSVLKMLLDNEETQFKKNLHNSGSSESELTASSWQG

HNSYGSTGPVNLDCVWKF

>NAC42_ARATH Stress Response Transcription factor JUNGBRUNNEN 1 OS=Arabidopsis thaliana GN=JUB1 PE=1 SV=1

MSGEGNLGKDHEEENEAPLPGFRFHPTDEELLGYYLRRKVENKTIKLELIKQIDIYKYDP

WDLPRVSSVGEKEWYFFCMRGRKYRNSVRPNRVTGSGFWKATGIDKPVYSNLDCVGLKKS

LVYYLGSAGKGTKTDWMMHEFRLPSTTKTDSPAQQAEVWTLCRIFKRVTSQRNPTILPPN

RKPVITLTDTCSKTSSLDSDHTSHRTVDSMSHEPPLPQPQNPYWNQHIVGFNQPTYTGND

NNLLMSFWNGNGGDFIGDSASWDELRSVIDGNTKP

>NAC10_ARATH Secondary cell wall fibres OS=Arabidopsis thaliana GN=NAC010 PE=2 SV=1

MSWCDGSDDNYDLNLERVSNTDHPSVQLKDQSQSCVTSRPDSKISAETPITTCPSCGHKL

HHHQDDQVGSIKDLPSLPAGVKFDPSDKEILMHLEAKVSSDKRKLHPLIDEFIPTLEGEN

GICYTHPEKLPGVSKDGQVRHFFHRPSKAYTTGTRKRRKVSTDEEGHETRWHKTGKTRPV

LSQSGETGFKKILVLYTNYGRQKKPEKTNWVMHQYHLGSSEDEKDGEPVLSKVFYQTQPR

QCGSMEPKPKNLVNLNRFSYENIQAGFGYEHGGKSEETTQVIRELVVREGDGSCSFLSFT

CDASKGKESFMKNQ

>NAC73_ARATH Secondary wall, wall fibres, cellulose, lignin 73 OS=Arabidopsis thaliana GN=NAC073 PE=2 SV=1

MTWCNDRSDVQTVERIIPSPGAAESPVASLPVSCHKTCPSCGHNFKFHEQAGIHDLPGLP

AGVKFDPTDQEVLEHLEGKVRDDAKKLHPLIDEFIRTIDGENGICYTHPEKLPGVNKDGT

VRHFFHRPSKAYTTGTRKRRKVHTDSDVGGETRWHKTGKTRPVLAGGRVRGYKKILVLYT

NYGKQKKPEKTNWVMHQYHLGTSEEEKEGELVVSKVFYQTQPRQCGGSVAAAATAKDRPY

LHGLGGGGGRHLHYHLHHNNGNGKSNGSGGTAGAGEYYHNIPAIISFNQTGIQNHLVHDS

QPFIP

>W6J8W6_GOSHI Senescence OS=Gossypium hirsutum PE=2 SV=1

MNKSNLGSVSSSDLIDAKLEEHQLCGSKHCPGCGHKLEGKPDWLGLPAGVKFDPTDQELI

EHLEAKVEAKDMKSHPLIDEFIPTIEGEDGICYTHPEKLPGVTTDGLSRHFFHRPSKAYT

TGTRKRRKIQTECDLQGGETRWHKTGKTRPVMVNGKQKGCKKILVLYTNFGKNRKPEKTN

WVMHQYHLGQHEEEKEGELVVSKIFYQTQPRQCNWSDRTATAVEVSNANDPNSRRDSGSG

SCSSKEVIPLRDEVTGAGVAAALSSYAAGAMDIQQLKSDHFSFTPFRKSFDEESSITNPQ

EAEWLKYSSFWPDPDNQDHHG

>NAC75_ARATH Cells wall Xylem formation OS=Arabidopsis thaliana GN=NAC075 PE=4 SV=1

MNKSNPAGSVTGSDIIDAKIEEHQLCGSKKCPSCGHKLEGKPQDWVGLPAGVKFDPTDQE

LIEHLEAKVLAKDFKSHPLIDEFIPTIEGEDGICYTHPEKLPGVTRDGLSRHFFHRPSKA

YTTGTRKRRKIQTECDNNLQGSSSSGETRWHKTGKTRPVMVNGKQKGCKKILVLYTNFGK

NRKPEKTNWVMHQYHLGTHEEEKEGELVVSKIFYQTQPRQCNWSSSTSSLNAIGGGGGEA

SSGGGGGEYHMRRDSGTTSGGSCSSSREIINVNPPNRSDEIGGVGGGVMAVAAAAAAVAA

GLPSYAMDQLSFVPFMKSFDEVARRETPQTGHATCEDVMAEQHRHRHQPSSSTSHHMAHD

HHHHHHQQQQQRHHAFNISQPTHPISTIISPSTSLHHASINILDDNPYHVHRILLPNENY

QTQQQLRQEGEEEHNDGKMGGRSASGLEELIMGCTSSTTHHDVKDGSSSMGNQQEAEWLK

YSTFWPAPDSSDNQDHHG

>U5I025_ROSHC Cell expansion, ethylene

MENVSAFINEDEQMELPPGFRFHPTDEELISHYLSPKVLDSGFTARAIGEVDLNKCEPWDLPWRAKMGEK

EWYFFCVRDRKYPTGLRTNRATEAGYWKATGKDKEIYKAKALVGMKKTLVFYKGRAPKGEKTNWVMHEYRLEGKYSAYNLPKTAKNEWVICRIFQKCSGGKKTHISGLVRESPFGNEFRPSFLPPLMDSPPYNNSDTRTTTVGETSHVSCFSDAMEDQKTQDDIIDSFNNNNNISSSSNNNNNSHLLASSSSFPFKPSVQNSYFSDQIAPNTGNMQYPDSGFMQDQSIMRLLTEPQAPSLRRSSSNYLGGQDVPSYSAAPIEFDSIWNY

>K4CH25_SOLLC ethylene biosynthesis, signaling, ripening NM_001279348.2

MESTDSSTGSHHQPQLPPGFRFHPTDEELVVHYLKKRVASVPLPVSIIAEVDLYKFDPWE

LPAKATFGEQEWYFFSPRDRKYPNGARPNRAATSGYWKATGTDKPVLTAGGTQKVGVKKA

LVFYGGKPPKGVKTNWIMHEYRLADNKTNNKPPGCDLANKKSLRLDDWVLCRIYKKNNTQ

RPIDHERDDLNIDMMMGSSSIHPSCIPNSMSMPNIFGQPKIPQLKSSNFGTTLIHDQNDQ

NLYEGGSQYSSKRPLANLYWNDQDGGASNDNSQSTKRFLTENMEDGLNMNARADEQNGSI

VSLLSQQQVLGSLSEGVFRQPYSGMNWYS

>Q56UP6_SOLLC nor gene no playing a role in ripening

MESTDSSTGTRHQPQLPPGFRFHPTDEELIVHYLKKRVAGAPIPVDIIGEIDLYKFDPWE

LPGKAIFGEQEWFFFSPRDRKYPNGARPNRAATSGYWKATGTDKPVFTSGGTQKVGVKKA

LVFYGGKPPKGVKTNWIMHEYRVVENKTNNKPLGCDNIVANKKGSLRLDDWVLCRIYKKN

NTQRSIDDLHDMLGSIPQNVPNSILQGIKPSNYGTILLENESNMYDGIMNNTNDIINNNN

RSIPQISSKRTMHGGLYWNNDEATTTTTTIDRNHSPNTKRFLVENNEDDGLNMNNISRIT

NHEQSSSIANFLSQFPQNPSIQQQQQQQEEVLGSLNDGVVFRQPYNQVTGMNWYS

>A0A0H3VAL9_9ROSA lignin biosynthesis

MESCVPPGFRFHPTEEELVGYYLKRKINSLHIDLDVIVDIDLYKIEPWDIQARCRLGYSE

QNEWYFFSHKDKKYPTGTRTNRATAAGFWKATGRDKAVLSRDNIIGMRKTLVFYKGRAPN

GNKTDWIMHEYRLQTSEHAPPQEEGWVVCRAFKKPSPSHRQGFEAWKHAYVRDHPSSSNF

SSEIPMLINPNQGFHQSFGSEEDHFVSNNIFLGNQLMELPQLESPTISTSFVTKENVLRS

NNISTDDYEAERSNNNSSNQHVDWRNLDNLLEVQLIDRASFSHPSLSSAPQNYEQETQNN

HLLGCFQDL

>D7L212_ARALL leaf senescence

MVEEGGVVVNQGGDQEVVDLPPGFRFHPTDEEIITHYLKEKVFNIRFTAAAIGQADLNKN

EPWDLPKIAKMGEKEFYFFCQRDRKYPTGMRTNRATVSGYWKATGKDKEIFRGKGCLVGM

KKTLVFYRGRAPKGEKTNWVMHEYRLDGKYSYHNLPKTARDEWVVCRVFHKSDPSTTITP

TSQLSRIDSLDNIDHLLDFSSLPPLIDPGFLGQSSPSFSGVRQQHDLKPVLHHPTTAPVD

NTFLSTQTFNFPYHSVHNSGSDFGYGAGSGNNDKGMIKLEHSLVSVSQETGLSSDVNTTA

TPEISSYPIMMNPAANAAVMDGSKSACDDLDDLIFWEDLYTS

>A0A1L3MZD9_ORYSI seed size

MGEQQQQVERQPDLPPGFRFHPTDEEIITFYLAPKVVDSRGFCVAAIGEVDLNKCEPWDL

PGKAKMNGEKEWYFYCQKDRKYPTGMRTNRATEAGYWKATGKDKEIFRNHHMLIGMKKTL

VFYKGRAPKGDKTNWVMHEYRLADASPPPPPSSAEPPRQDDWAVCRIFHKSSGIKKPVPV

APHQVPAAANYQQQQQMAMASAGIIQVPMQMQMPSMSDQLQMLDDFSTTASLSLMAPPPP

PPAGFPLQINSGAHPQQFVGNPSMYYHQQQQMDMAGGGFVVSEPSSLVVSPQDAADQNNN

AADISSMACTMDAAIWKY

>A0A1L3MZG8_ORYSI seed size

MAMTPQLAFSRMPPGFRFQPTDEQLVVDYLQRRTAAQPCVTPDITDIDVYNVDPWQLPAM

AMYGSDHDRYFFTMAAREAQARRTTPSGFWKPTGTKKTIFVVAGGHEVPTAVKRRFVFYL

GHHQPSGSNNNNKTSWIMHEYRLMNSPRAAVPSSSSVNRLPTDDLTEEMVLCRISNKDLP

KPPFIHNGLLQFSSVGLNGDGYNYLILDHLEPPAMEYPNVDIGNVDDAAAADDDPGDLDE

EIDDSMQRNHGG

>A2WQE4_ORYSI Seed size

MGEQQQQVERQPDLPPGFRFHPTDEEIITFYLAPKVVDSRGFCVAAIGEVDLNKCEPWDL

PGKAKMNGEKEWYFYCQKDRKYPTGMRTNRATEAGYWKATGKDKEIFRNHHMLIGMKKTL

VFYKGRAPKGDKTNWVMHEYRLADASPPQPPPPPSSAEPPRQDDWAVCRIFHKSSGIKKP

VQVPMQMPMQMQMPVAHQVPAANYQQQMAMASASIIQVPMQMQMPSMSDQLQMLDDFSTG

SLMAPPPPPPSYSTLPGFPLQINGGAQQFVGNPSMYYQQQQQQQQQQMDMAAGGFVVSEP

SSLVVSPQDAADQNNAADISSVACNMDATIWKY

>A0A1L2DX18_CARPA carotenoides biosintesis

MGDGSMNLPPGFQFCPTDEELITHFLYRKASRLPCHPNIPHLHFYRSDPSHLQGKAVVSG

NRYYFFTQVGSKEINGGKTENGYWKAVDEEEEEEEEGEAIFSRGNKLLIGTKKHLVFFLG

QPPSAIQTNWFMEQYHLPTSNITSSERKKWVLCRVHDGGEGNSQGFSHSSSGDNDEDGIE

LSCLDEMFFSLHDDLEDVTSPN

>A0A178W8K0_ARATH Leaf senescence

MEVTSQSTLPPGFRFHPTDEELIVYYLRNQTMSKPCPVSIIPEVDIYKFDPWQLPEKTEF

GENEWYFFSPRERKYPNGVRPNRAAVSGYWKATGTDKAIHSGSSNVGVKKALVFYKGRPP

KGIKTDWIMHEYRLHDSRKASTKRNGSMRLDEWVLCRIYKKRGASKLLNEQEGFMDEVLM

EDETKVVVNEAERRTEEEIMMMTSMKLPRTCSLAHLLEMDYMGPVSHIDNFSQFDHLHQP

DSESSWFGDLQFNQDEILNHHRQAMFKF

>A0A059SWK2_HEVBR Stress response / Osmotic NAC transcription factor OS=Hevea brasiliensis GN=NAC1 PE=2 SV=1

MTWCNNNSVDERAIQLVTASSKENTVIDTKIDQIRTITCPSCGHDIQLQDQAGGIVHDLP

GLPAGVKFDPTDQEILEHLEAKILSDMRKLHPLIDEFIPTIEGENGICYTHPEKLPGVSN

DGQVRHFFHRPSKAYTTGTRKRRKVHTDEDGSETRWHKTGKTRPVFSGGTVKGFKKILVL

YTNYGRQRKPEKTNWVMHQYHLGQNEEEKEGELVVSKVFYQTQPRQCGSSIKDSLDQNLR

NRIDHENAPLAKNPALVDYYNNPPFFSYDHGSHNRESPSQLIPNMVIQGDGSSFFRLAAD

TSKGKLQRR

>A0A2D3HXD3_9ROSA Lignificaction NAC protein OS=Eriobotrya japonica GN=NAC3 PE=2 SV=1

MENTSGFIMEDEQMELPPGFRFHPTDEELISHYLSPKVLDNFFCARAIGEVDLNKCEPWD

LPWKAKMGEKEWYFFCVRDRKYPTGLRTNRATEAGYWKATGKDKEIYKAKTLAGMKKTLV

FYKGRAPKGVKTNWVMHEYRLEGESSAHNLPKTAKNEWVICRIFQKSSGGKKTHISGLLR

PSPFGNELRSSLLPPLMDSPAYNSDARTTATACETSHVSCFSDPAEDQKTEDDIMDSFNN

GNNHHHHHNNIHFASSSRSNPSAHLNSFYSNQMTPNIGLLQHQNSVLMQDQSLLTMLVEN

QAPYLRRSAKIEFSPDTGLSIDASSAVSNREMVQDDPSYSSAPIELDGLWNY

>A0A1P8BFW3_ARATH xylem tracheary elements

MNLPPGFRFFPTDEELVVHFLHRKASLLPCHPDVIPDLDLYHYDPWDLPGKALGEGRQWY

FYSRKTQERVTSNGYWGSMGMDEPIYTSSTHKKVGIKKYLTFYLGDSQTNWIMQEYSLPD

SSSSSSRSSKRSSRASSSSHKPVSTLFCFISLKLSALT

>K9M275_ROSHC Stress response / Osmotic

MMQEMEKFAVQAGTFREDDQIDLPPGFRFHPTDEELISHYLHKKVIDSNFGCKAIGDVDL

NKSEPWDLPYKAKMGEKEWYFFCVRDRKYPTGLRTNRATEAGYWKATGKDKEIYKGKSLV

GMKKTLVFYGGRAPKGEKSNWVMHEYRLEGKFSVHNLPKSARNEWVICRVFEKSAGGKKI

HITGLVNSGPHGTEMGSSGLPPLLDSAPYNGAKTRPASESNYVPCFSNHESIDSQRNQGI

VDYINSNPLFSVSSNPLNNNNAFPRVTYQNSLCSAPVSSANFQFPSSVLMQDHLRALLEN

NVSFMRQNFKTERDMVSVSQETGLTTDVNPEISSVMPNPEMGRRPFDDQDNFWM

>K4BBX4_SOLLC Growth & development / root meristem

MEIVCGFGGREEEMELPPGFRFHPTDEELITHYLAPKVLDSGFCAIAIGEVDLNKVEPWD

LPWKAKMGEKEWYFFCMRDKKYPTGQRTNRATEAGYWKATGKDKEIFKSKTLVGMKKTLV

FYKGRAPRGEKTNWVMHEYRLDGKYSIHNLPKTAKNECVICRIFKKNSGGKKIAISNLTR

SDSITDNSRSSNLPPLMDLSPYNKITTTARSSGETCNSHVTCFSDSMEDQKPQTYDHHHL

LSSSPVSVDFPQNVPNDSMTYMGNFQYGDSGLMQDNSIMRLLIDNNYGSESKQSLRGYED

HDINISSTGPVDIDCLWNY

>Q52QR3_SOYBN GmNAC3 Stress response Osmotic ABA Jasmonate

MGVPERDPLAQLSLPPGFRFYPTDEELLVQYLCRKVAGHHFSLPIIAEVDLYKFDPWVLP

GKAVFGEKEWYFFSPRDRKYPNGSRPNRVAGSGYWKATGTDKIITTEGRKVGIKKALVFY

IGKAPKGSKTNWIMHEYRLLDSSRKHNLGTAKLDDWVLCRIYKKNSSSQKVEANFLAMEC

SNGSSPSSSSHVDDMLGSLPEINDRCFTLPRVNSLRTMHQQDEKFGSPNMGSGFFSDWVN

STDLDSISEFESGCQTQRMVNYDCNDFFVPSLPPLGHVDYMVDAPLEEEVQSGVRTRRVD

GPGHFQPNPDTRLLPGSGDPFGFGFIMGQQVEFGFRD

>Q52QR5_SOYBN GmNAC1 Senescence / death

MENRTSSVLPPGFRFHPTDEELIVYYLCNQASSRPCPASIIPEVDIYKFDPWELPDKTDF

GEKEWYFFSPRERKYPNGVRPNRATVSGYWKATGTDKAIYSGSKHVGVKKALVFYKGKPP

KGLKTDWIMHEYRLIGSRRQANRQVGSMRLDDWVLCRIYKKKNIGKSMEAKEDYPIAQIN

LTPANNNSEQELVKFPRTSSLTHLLEMDYLGPISHILPDASYNSTFDFQINTANGGIDPF

VKPQLVEIPYATDSGKYQVKQNSTINPTIFVNQVYDQRG

>Q52QR2_SOYBN GmNAC4 Stress respose / Osmotic

MGVPEEDPLSQLSLPPGFRFYPTDEELLVQYLCRKVAGHHFSLPIIAEIDLYKFDPWVLP

SKAIFGEKEWYFFSPRDRKYPNGSRPNRVAGSGYWKATGTDKIITTEGRKVGIKKALVFY

IGKAPKGTKTNWIMHEYRLLDSSRKNTGTKLDDWVLCRIYKKNSSAQKAVQNGVVPSNEH

TQYSNGSSSSSSSQLDDVLESLPAIDERCFPMPRVNTLQQQQHEEKVNVQNLGEGGLLDW

TNPSVLNSVVDFVSGNNNHNQLVQDQTQGMVNYNACNDLYVPALCHVGTSVPQKMEEEVQ

SGVRNQRVQNNSWFLQNDFTQGFQNSVDTSGFKYPVQPVGFGFRN

>Q52QR1_SOYBN GmNAC5 Senescence / death

MENVSVLLCNKEKDQMDLPPGFRFHPTDEELISHYLYRKVTDTNFSARAIGEVDLNRSEP

WDLPWKAKMGEKEWYFFCVRDRKYPTGLRTNRATESGYWKATGKDKEIFRGKSLVGMKKT

LVFYKGRAPKGEKTDWVMHEYRLDGKFSFHNLPKTAKNEWVICRVFQKSSGVKRTHISGM

MMLDSYGNEMVYSSSALPPLTDSSPSIGNNTKALSVTDSAYVPCFSNPIDVPRGIFDSLN

NINISINSNTLYGVSSNHSFYNTQGVQLQAPPTLPLPSSSNHYLRAFLENQGNGSNMSNN

GFEPEREMVSVSQKTSLSTDVKAEISSLGKRHFENQNNPIASAAAVAPMDLATLWNY

>M5WG30_PRUPE Anthocyanin biosynthesis OS=Prunus persica GN=NAC1 PE=2 SV=1

MESTDSSTASQQQQQQQPQPPPQPNLPPGFRFHPTDEELVVHYLKKKVTSAPLPVAIIAE

IELYKFDPWELPAKATFGEQEWYFFSPRDRKYPNGARPNRAATSGYWKATGTDKPVLTSG

GTQKVGVKKALVFYGGKPPKGIKTNWIMHEYRLADSKTSNKPPGCDLGNKKNSLRLDDWV

LCRIYKKNNSHRPMDLEREDSMEDMMGPLMPPSISHVGHHQNMNLHLPKSNTNYGPPFIE

NDQIIFDGIMSSTDGSASLSNGTSQLPLKRSIVPSLYWNDQEDDQTAGASSSKRVVQLHQ

LDSGTNNSVAANNNSTSIANLLSQLPQTPPLHQHAMLGSLGDGLFRTPYQLPGMNWFSES

NLG

>A0A0C4MXF0_BRANA Stress ROS ac OS=Brassica napus GN=NAC56-1.1 PE=2 SV=1

MESTDSSGGPPPPQPNLPPGFRFHPTDEELVVHYLKRKAASAPLPVSIIADVDLYKFDPW

ELPAKASFGEQEWYFFSPRDRKYPNGARPNRAATSGYWKATGTDKPVLASDGNQKVGVKK

ALVFYSGKPPKGVKSDWIMHEYRLIDNKPNNRPPGCDYGNKKNSLRLDDWVLCRIYKKNN

AGRHVDNDKDHDMIDYIFRKIPSGLNHSVSRSMNFFPGRFSGGYGMFSDGDPGFYDGSGM

MCSNGADAVNLNVCNGNLIADVVGNGLNPVSSSGPMMMMANLKRALPVPYWPVADEEQEV

SPSKRFHGVGGGGDCSNMSSSMVEEPPPFMQQQGGVLGDGLFRTTSYQLPGLNWYSS

>K7P5D7_SOLLC Stress response /osmotic NAC3 OS=Solanum lycopersicum PE=2 SV=1

MESTDSSTGSHHQPQLPPGFRFHPTDEELVVHYLKKRVASVPLPVSIIAEVDLYKFDPWE

LPAKATFGEQEWYFFSPRDRKYPNGARPNRAATSGYWKATGTDKPVLTAGGTQKVGVKKA

LVFYGGKPPKGVKTNWIMHEYRLADNKTNNKPPGCDLANKKSLRLDDWVLCRIYKKNNTQ

RPIDHERDDLNIDMMMGSSSIHPSCIPNSMSMPNIFGQPKIPQLKSSNFGTTLIHDQNDQ

NLYEGGSQYSSKRPLANLYWNDQDGGASNDNSQSTKRFLTENMEDGLNMNARADEQNGSI

VSLLSQQQVLGSLSEGVFRQPYSGMNWYF

>E2FJQ8_MUSAC Ripening / ethylene signaling / Resistance NAC protein OS=Musa acuminata AAA Group PE=2 SV=1

MGLRDIESTLPPGFRFYPSDEELVCHYLYQKVTDGFRTSEGTMVEVDLHTCEPWELPDVA

KLSANEWYFFSFRDRKYATGSRTNRATKSGYWKATGKDRTICDPRTQAMVGMRKTLVFYR

GRAPNGVKSGWVMHEFRLETPHSPSKDWVLCRVFHKRKGDSEHDEAGSSSLVYQPTMPDA

CHDQQLGSSLSAALLQQDDTSPNPFLMNMALLQCNLLDLPEEMMGSAPMAGMSSGCEDEF

GCLSELGLEHSIGEEGMVRWEG

>A0A1S5VZG0_LITCN Senescence NAC transcription factor 13 OS=Litchi chinensis GN=NAC13 PE=2 SV=1

MVHTKNPESSLPPGFRFHPTDEELILHYLKRKLTSSPFPGSIIADVDIYKFNPWDLPAKA

AFGEKEWYFFSPRDRKYPNGARPNRAAASGYWKATGTDKIIVASSMGAGGGGVPENIGVK

KALVFYTGKPPKGVKTSWIMHEYRLTETPNNFIVNKPMKPKDSSMRLDDWVLCRIYRKHN

SSPSSAAATSDQDQEEEDEQFVQETLLPILKSPPRNATLNPQKSSSFSNLLDAMDYSMLS

SFLSDNQMYQTGFEPIPMHTSGLLDQPCFNNGTNNGCSSSFMLQKLPQLNSPLLDNVENK

LKRPHSSVDELMFHPSKKQHISYSCTTLTNTASTHDHQSDPLQYNFLHQSLLNPQLLLSP

HLQFQG
