# Supplementary material for: Genome-wide analysis of the NAC transcription factor family and their expression during the development and ripening of the Fragaria × ananassa fruits
Source: PLoS One. 2018 May 3;13(5):e0196953. doi: 10.1371/journal.pone.0196953 (PMC5933797; doi:10.1371/journal.pone.0196953)
Supplement: S5 Table — (DOCX) [file pone.0196953.s005.docx]

| **Sequence** | **Forward primer (5’-3’)** | **Reverse primer (5’-3’)** |
| --- | --- | --- |
| FaNAC006 | GGTTGTGTGCAGGGTTTTCC | TCCCCAAAAGAGTTGAGCCT |
| FaNAC021 | GACCTCGAACGGTAGCCAAA | GCGCTGGAATTTTCGTCGTT |
| FaNAC022 | TGGGTGATGCACCAGTACC | GCACTAGCTCCCCATCTTTTT |
| FaNAC035 | TGACAAGCCGGTTTTGAGTA | TCACCCCAACTTTCCGAGTA |
| FaNAC042 | AAGCGCAAGGTCTACTCCTG | GACTTGCAGACCTCAACCTCA |
| FaNAC092 | TTCCGCCCTTCTTTACTGCC | CCTGGGCTTTCTGATCCTCC |

**S5 Table. Primers used for the qRT-PCR quantitation of the six NAC genes related to the development and ripening of *Fragaria* x *ananassa*.**
