# Supplementary material for: Genome-wide analysis of the NAC transcription factor family and their expression during the development and ripening of the Fragaria × ananassa fruits
Source: PLoS One. 2018 May 3;13(5):e0196953. doi: 10.1371/journal.pone.0196953 (PMC5933797; doi:10.1371/journal.pone.0196953)
Supplement: S6 Table — (DOCX) [file pone.0196953.s006.docx]

>FvNAC001 FvH4_1g06530.1

MNKSNIRSSDLIDAKLEEHQMCGSKQCPGCGHKLEGKPDWLGLPAGVKFDPTDQELIEHL

EAKVEAKDTKSHPLIDEFIPTIEGEDGICYTHPEKLPGVTRDGLSRHFFHRPSKAYTTGT

RKRRKIQTECDHLQAGGETRWHKTGKTRPVMVNGKQKGCKKILVLYTNFGKNRKPEKTNW

VMHQYHLGQHEEEKEGELVVSKIFYQTQPRQCNWSSERSSAASASLTGEGSSDLRRDSGG

SGSCTSSKEIIVSHNTHLRDHHDQMSAAAAAQVNVVGQPLTSYNAIDIHQLKSDHFSFVP

FRKSFDEVGIGEASTAREPSIASGTCEDLRERPQLHHVAHDQLLHHHQHQQQVHHEQQQH

QHGQTHLQQQIATAASFHISRPSHPISTIISPPSLHHSATSIMLDQDAYHVSRSIMLQNE

HFQQQQQQQQQQQQQQQQQQQHHKQMGGRSASGLEELIMACTSSSNVKEESSSMANPQEA

EWMKYSPFWPDPDNTDHHG

>FvNAC002 FvH4_1g09330.1

MANENSPAKPPPWFIFRPTDTRLIRTFLIPRIRGQDLPMNDFARRDVFADPPLDLAIAEG

YSEEKQEIYFFNQTSRRTPHWKPTHRSEDLIKSGDTVIGRKKKFYHAQNPGKTIKWYLDQ

YTAEGENSDVAVYKLHTGREPAQSLYTAEAQNQNLTVPPPQSGSSLLPVLPCNPPLLQQE

YSQWNHSLQHNGSSPLLQQAYSPWNPMDLQAFSQWNESLPQHNGSSALPLPEWNPPLPYN

GLSSLPLPQGNPPFLQQDNTQTGFPTDWSESGLLQQILYSNQPTNQSQNPLPQGNPPFLQ

QDNTQTGFPTDGSAVLSFPTQQYVFQPEDSGQHLQEASTAGNPVLQDGSALPPYPSQQDA

FQPEDSGHLGSQQILHANIQGNPQNIFWSDFYQYYLTQLHNNPNANTNNDQPSSSNPPEH

>FvNAC003 FvH4_1g12050.1

MESIESTVPPGFRFHPTDEELVGYYLRKKVASQKIDLDVIRDIDLYRIEPWDLQDRCRIG

YEEQNEWYFFSHKDKKYPTGTRTNRATMAGFWKATGRDKSVYDKSKLIGMRKTLEEGWVV

CRAFKKRTSTQNKSMEGWDSSYFYDELHGVSSIVDPTDFISRQPQRAAQSFLAAQSFLCK

QEITAEADNFNSTFMPSDHFVQLPQLESPSLPLIKKPSSSMSLISSSENNVEEEVEWINN

NRGCSSNSNNAKKAAAVTDWRDLDKFVASQLSHEDKFNELGDHGEYSSFGVHDHDDSDMA

ALLLLQRDDGNKLNGFLSSGSDCDLGICIFEK

>FvNAC004 FvH4_1g25150.1

MKAELDLPAGFRFHPTDEELVNHYLIKKCASQNISVPIIKEIDLYKFDPWQLPEMALYGE

KEWYFFSPRDRKYPNGSRPNRAAGTGYWKATGADKHIGKPKALGIKKALVFYAGKAPKGV

KTNWIMHEYRLANVDRSASKKNHNLRLDDWVLCRIYNKKGSIEKYNTATVERNRFPTYSE

IMDHEEKPELKNIQFNVAPSQVPQMPSAVAPVTQHTDVLSGDYSAPRLQHTQHTTDYSSC

SDHVFSPEVQSEFQWTSSPEFSELDNSMNMNFLESNDQFNYMDGFSLADIDDPFGNQAQA

QAQTQYQMDQQDLFMYLQNQI

>FvNAC005 FvH4_1g27330.1

MAPVSLPPGFRFHPTDEELVAYYLKRKINGRKIELEVIPEVDLYKCEPWDLPGKSLLPSK

DLEWYFFSPRDRKYPNGSRTNRATKAGYWKATGKDRKVNSQTRAVGMKKTLVYYRGRAPH

GIRTDWVMHEYRLDERECETAQGLQDAYALCRIFKKTATGPKIGEHYGSTSSTTNYQLMT

SDHHSSSVELYSDSRCDHQDFESSRYNNQMAMNNTPCSSNMVQTSSSSSLDHMGRKRDGK

WSQFLSKDAFNSFSSFPNQGAIPYPPSKVDIALECARLQHRLTLPPLEVEDFPHVGFTDF

KNLQYSDPGLEGTGTETDALQEILSVAHVSQEMINQSNNFADQTWATANYAPGANDFSFM

VDRDAHYNQIITTHHDMNPMRYVDKPWENSYIRSIEIGDLEDDDFRTDNTTENLRWIGMS

DKDLEKSFTEEHKIVPIQNIASFRREEHGHQVEGDQTEHHSDMIKEFNDGETNDFSLGFI

NDDPDENFLQDGNIETDYSSSPSFEVIEEIQVNHGMFVSTRQVADTFFHQIMPSETVKVH

LNPVLVHSYLVEKVDDAQQNSKNTRGSFFEMFKEFVMEKFVGVVISMNPWRKMASAMVCA

VTLLLMHTIYVGEHLEDEKLRDAFTATTAIVVEEKGSSNIISKKKKEVCLVNIRGGSSCS

LFLKKIGIFLTVSLALCTVWANHSIITS

>FvNAC006 FvH4_1g27900.1

MMEEEGMVVNQGHDELMALPPGFRFHPTDEEIITCYLTEKAVDSNFTATAIGEADLNKCE

PWDLPNKAKMGEREWYFFCQRDRKYPTGMRTNRATESGYWKATGKDKEIFKRKTLVGMKK

TLVFYTGRAPKGEKTNWVMHEYRLHGNLSKSTKDEWVVCRVFHKNMGLKKTSSPLSPTDQ

GAGGQLLRLNSFGDDFLDSSSLPPLMDPTPSYDKSAGSYSGYYGEHNDHDTKGNITAAPL

SIPAANKNTTPADLQYYFEVGSSTRQMNRFQLPNAAGSNQMSNPMATSTMLYPQIHHHHQ

LIPNPSFSFQPNNNLDYYLRQEIRGPRTSIPNNQGSSFNFQSSAIDHQVTLRAMAAAKNN

HHHIETSVTTAGSGGLDSRQCKLEQYSSNQSMFSVSQDTGLSTADMNTTEISSSVVTKQE

IEVGSSNTLYDDHLESPSVVPISDIEGLWDY

>FvNAC041 FvH4_4g12410.1

MCASCPSVQNPMAVDDDLPPGYKFRPDKEQLLVHYLRPKLDGEDFAQELVPFCDLYGKKE

PWQIWNDYFQELSATDKERTDLYFFTQLKTKTPKGKHIKRTVGSGTWKGEDAPKEVLAAS

QRVIGKRRRFRYGNDKDSAQNDRWLMHEFELDASLLRNKRKAKDYVLCIVRKKSRSVKKN

SEDQEGDETSCDGGDDQQDGDLPEAKKNNDHKDSETVSLEEMENLLMSDD

>FvNAC042 FvH4_4g23130.1

MEKLSFVRNGELRLPPGFRFHPTDEELVLQYLKRKVYSCPLPASIIPEVEVCKSDPWDLP

GDLEQERYFFSTREAKYPNGNRSNRATGSGYWKATGLDKQIVASRGNQVVGMKKTLVFYR

GKPPHGARTDWIMHEYRLVLAEDKNNSTTQSHVPVNNWVLCRIFLKKRGSGKNEEEQVQV

QACNVDRVAKKPRITRPVFYDFMTKDRAKLSLAPCSSSSGSSGVTDVVSSETEEREESSS

CNSLSYFRRKQ

>FvNAC043 FvH4_4g28090.1

MAGPSWLVDKKRIATKIRSASASDPGRIKWQSNPTRACPNCQYTIDNSDVSQEWPGLPKG

VKFDPTDQEIIWHLLAKTGAGDLRPHPFIDAFITDFDGDDGVLCSHPKKLPGIKQDGSVS

HFFHVAIKAYNTGTRKRRKIHDDDYGDIRWHKTGRTKPVILDGVQRGCKKIMVLYTSTAK

GGKAEKTNWVMHQYHLGTDEDEKEGEYVISKIYYQQQQVKQADKTDQNSPESSDPVIQAD

PVTPKSVIPDPVIQANPVTPKSVIPEALNPDFNLVEDIPITCAEPNSVTPEPPRPERRQS

TLGFGEGTHIASTDPFLQMQMNHHDMDCFEDEVHHGYEQSGSHDQHSVEIPNDEAINNDV

NHTQEDPKWWDSESQNLLDSQQLVEGLSLCDDLLQSQSPIRGEHGNEPAQIKPCLADYAK

LGAENLKKDLEECQNITLDPANMELGTPPDFRLSQLEFGSQDSFLSWGGKLAE

>FvNAC045 FvH4_4g31070.1

MEANSSSGLPPGFRFHPTDEELIVYYLKNQAISKPCPVSIIPEVDIYKFDPWQLPEKAEF

GENEWYFFSPRDRKYPNGVRPNRATVSGYWKATGTDKAIHSASKYVGVKKALVFYKGRPP

KGIKTDWIMHEYRLSDSKRQATKQLGSMRLDDWVLCRIYKKRHVNKAYMDPKLEDSSPQM

DHFTAANDVKEEHGVNFPRNFSISSFLDMDYLGPISQLLNENPTYDFQNMLASNAGLNGQ

TFQYGDQIPYQSADSGKFQLMNQSNSTFNQPLFVNPAYNNNNMNGLNH

>FvNAC046 FvH4_4g31420.1

MDQERSSSGDMSAADQDKIDDVMLPGFRFHPTDEELVGFYLKRKIQQRPLSIELIKQLDI

YKFDPWDLPKLAASGEKEWYFYCPRDRKYRNSTRPNRVTGAGFWKATGTDRPIYSSEGNS

NKCIGLKKSLVFYKGRAAKGVKTDWMMHEFRLPSLTDSSVVLPSKRSFMDKIAIPANDSW

AICRIFKKTNTSAHQRAISHHSWVSQTLPEPNTFQDHVLGSSRLRAQTTNTTQYSGNIDT

QQISTSTTTSTLSPVDNYYANNPYKTLHPSIPSNLTPYHQYFPISNAGDYFNPNFTFSPL

ENPAAKCSVDVSSLLLNMSSSVLGDFGTKLASAESSTTFDFSSTTGSQEQQCNNGFSSSL

TTIPHHELMHGNGVVGSHDHSSIDALMKNITNHVNSNATETDDHEHWESSLQSSSIGFPF

MSSLPVIPMNIGGGDAWKSNLTWDSSPCPSEMSTTRCYS

>FvNAC044 FvH4_4g30290.1

MAFLPSRFKKKKALKGDFDLLECSKVDVGMTEQECNLPPGYRFSPVDDELVLFYLFNKIV

GRNIPLTDVVKEIDLYEHDPDQLPQDDYRHGTKRCEAYYFTNVEQVYSREGKVTKRTTKG

GYWKVTSEGKKVTYGDDEIVVGFKTSMLFNKGHVPAPRGTISSFIMHEYRVNPSIVPVDV

LNDSIRAKIERFVVCKIVNKEKEARQEGPPAYKLPPELPSHEDEESS

>FvNAC076 FvH4_6g07970.1

MVSADDLVIIFRQQPGFRFHPTQEELVLYYLKRKICHKTFKLNIITETDVYKCEPEELPG

LSLLQTGDRQWFFFSPINCKYPKKSTRMNRVTRLGYWKVTGKDQTVIHNSRPVGVKKTLI

FYKGRAPRGNRTDWVMHEYTLHDEELKRCGNVPDPYALCKVYKKSGLGPKTGEHYWAPFQ

EEDCSDMDEVVQETPVKKIEDQIRVTPGVNSYSESYEIQHSSFDEDMEEFMKRIAGEAEQ

DMYYEASMFLQDLRPRFDEHLCMDPELDAGNYIDHDQLNAEESSQFHNQDE

>FvNAC073 FvH4_6g05730.1

MNMAAPVMQIKGMRFHPTDVELIEYFLHDKADADQAGCYSSFIHECNDLYGEKAMEPWDI

WDKYGGSTYEEGEPLYFYTKRIKLNSDGKRFNRKVGSGTWSGEYSKDVVVSSQNGITKIG

TMRNLRYEMIKGCDATQNGAWLMHEYESAFDIEHDYVICALKRNPRRAQANQEDAEITKK

NSVKKRKSASFVDHQSRKRKKKLKEEEIYEIIDLEPSNMVKEERKEETAYDDQGRSSCTQ

LPEQQPYHHDQDNVTDYFSGRLGNNYDDGICLDDLIGPINSPYYKSFQYENQLYHGDDSK

FNFMDYTNGGTSQLPENQLYHRSDDSKFNVMDYMNIGGMSQLPDDQATVDYLENF

>FvNAC074 FvH4_6g05740.1

MERFPVGFRFHPTDNDLVSHYLRNKAMLGDGFRSPYVSDCPEFYSSEPWTVWEKYGGNSV

EDGEPLFFFIKRKKLNPKGSRFDRKVGTGTWSGQYFAKVMEEGKVIGIKRNFRYEGGSSS

DQNNAWLMDEYELNKNDVTVVCVLRKNPTKPPPKTNETKRKLKDGVDGIEQDLDQRRKLI

SNLDQRRKLRGNYMPEKEGTSYREEQGGSSSSIDHHQASSSWTPKQEDYRPELAPDLAAA

DDFVFYTHDELLASTSKSNDFLDRFYEDNNFDQLVHKQVF

>FvNAC075 FvH4_6g06070.1

MELGFSPNQKPYQTELMINLEDSQLPPGFRFHPTDEELIGYYLLRKVMDSNFTCRAITQV

DLNKCEPWQLPEKAKMGEKEWYLFSLRDKKYPTGIRTNRATEAGYWKATGKDREIFSSKT

GSLLGMRKTLVFYHGRAPNGMKSNWVMHEYRLECRFAYRHLLRNSQEWVISRVFQKFKKS

NGPECKKIRMIAEPSSPASSSVSLPLLFDSSPYFSNGNANSLTDRDSCSHDNRTPRDHVS

CFSTIANTATFNHLMTSSDLAPPPQPPTPSLHGFGGLSDFPSLRSLQENLQLPLFFPQGM

QPLQPYSSTSGSFDLGDWSSLGNWR

>FvNAC077 FvH4_6g15670.1

MAEMEQTAIENHNLQLPAGFRFHPSDEELIVHYLKNKVTSSPLPATIITELDLYKYNPWE

LPPKALFGKEEWYFFTPRDRKYPNGTRPNRAAGLGYWKATGTDKHIFSSCGTKSIGVKKA

LVFYTGHPPKGVKTEWIMNEYRLLDTTIWSTTSKQKGSMRLDHWVLCRVRQKCNNMRNIW

EDQNITPSYKLSGNFRQVVDEPCSKEASPSIEMVRSYLYKDYPMLPYMFASQKVLPTIKT

TSSISFQDTTNGNEKSCSPTPVYHEANSNKINNGGQLLVSSLDSLINPLKRKTEGNDHPQ

RRCSVAPSKKICNRDYQMEEVSNGSMMNPWGLDNQLGADNLNVDQWSSIIQYQELSQLIG

FPCN

>FvNAC079 FvH4_6g19430.1

MEDFPPGFRFYPTEEELVSFYLPHKLDGTRKDVNRVMDRIIPVLDIYEFNPWDLPRNSGE

ICHGDQEQWFFFIPRQESEARGGRPKRLTTTGYWKATGSPSYVYSSSSSSTNHGAIGLKR

TMVFYTGRAPKGSKTEWKMNEYKAIEEDDQLHAPSSSSNATPAPALRQEFSLCRVYKKSK

CLRAFDRRPSGTGDIMRRPTVTVQLPALPQLGTDHHNEGSTYPTAISHAQNIIPAAPNSY

NVEMERISTLSSSGSSSSGDHGQPSQSGQSTGSLPVAVDNGEPLWELDQLDWFN

>FvNAC078 FvH4_6g17240.1

MEGNNQENNEEATTTDEYFRSLPPGYRFNPTDQELILYYLKPKILNQPLPRNRIHERNIY

QFHPRKLSEKFKKIREDGWYFFSTRKRKYQNGRRPNRKADDGYWKATGSGIDIKGENGEV

IGHKKVLDYKKGAHPLNIKTDWKMLEYKLKDEDEDNPLPKRKKGEGNMKLDLVLCKVYLN

TRANGNTNNNGAITTEISDQEMVTDSGTSHYRETVLKQEADQVQPLDSSLMSSLQRQVHS

NTTGAFNHMDHISTSSTIPETCFHSSNPMNNGVMVTDTVTSHRRHNNLVPKQEAGQVAPL

DSSLVYSPQPQVHNNTTGGFNHLNHISNSSSSTSMSNGVYDSTNCTHYGEPMHNGSSGSV

YQWGSSFTQLHHQNYYMYPEQMPSSSTSYTSEHPLQYLSTEIWTQLQPENHEMSRLDDST

FVSLPPHVREYNSMLVNIKDEAEMMPFKHEADDDDDDDGLQFLSIPDEVHEDDPKTD

>FvNAC081 FvH4_6g23470.1

MSNGQVDDYFRSFPPGYQFFPTDEELIISYLKRKILNQELPMNRIRDENIYNYHPRTLAG

NESTPKDLSLIEQGGYWKATKRDETIKTQGGEEIGYKMHLDFYDGTHAQGTKTNWKMHEY

RINEENGISSRNNNNKGEGSSRRLDEWVLYKIYINKNFKADTNNHSSKTPDNFEVVLPVT

NVTMTIKTYHQLFSMKIKINLATPHSSTTQL

>FvNAC080 FvH4_6g23460.1

MESNYQEAPTFFEDPVDVYFHFLPPGYRFAPTDAELIIYYLQKKILKQEPPLNKIREIDI

YLYHPQYLAETQSNRDGEREWYFFTPRERKYLHGSRPNRTQRSGNGFWKVTGKDEVITSA

AGERIGFKNTLDYFEGTHSENQKTKWKMHEYRLKKSERDEEIAPSNNEKAQRDMKLDDCV

LCKIYTSNRCKVGKIIKRSKSTSAKRSFNSDPNRDVQHEYQDQDCGAHPSGPVNNFSNQT

NTYDIMLSSLEEEVRAFDHWLANETATKKRIE

>FvNAC082 FvH4_6g26330.1

MSSDLVGFRFYPTDEELISYFLDNKNNVHTTAQVPSMATFNYNNIMPEVDLYGDVEPWQI

WETYGGSELYDQDMLFFTQHKMVNPDGLRIQRKVGSGGTWSEGEPGKLIFDSKLMQKPIG

QKRKFRYEKKGSEHNGCWYLEEYSLLSMNSASYVLCRLRQNIRKGRKRKEISLRQESPMN

YAPVGFRFHPTDQQLISYFLHKKVTEEASLYSYKNIVCEFDLFGSTEPWEIWNMYGGHEL

RNQDLFFFTKLKRASLKSSRILRKIGSSGTWSETDASKLIKDEAKQKHIGRRRKLRYESK

CSKQHGHWYLDEYSLLDSESDYVICRLRKNDKSSPPSSEWNGNKRTYSALCQESKETLSS

VTNKKLLRKQSNETMNVNNKKNNTLSDQTIAQTKRMQPGTKIKLFGVEIDSDLKYV

>FvNAC083 FvH4_6g26730.1

MTHCNDLGQEDPRLIAKDGLTTRTCPSCGYQIKYQQQAGIQNLPGLPAGVKFDPTDQEVL

QHLEGKVLSSTDIAAGNKLHHHPLIDQFIPTIEGENGICYTHPHKLPGVSKDGLVRHFFH

RPSKAYTTGSRKRRKVHTDRDSSSEGGETRWHKTGKTRPVFAGGKVKGYKKILVLYTNYG

KQRKPEKTNWVMHQYHLGNNEEEKDGELVVSKVFYQTQPRQCGNSGMKDFNNNSAAKVLM

NGQSEHSEALVQYCNNVNSFISFDQNGQDRSSSNPQIIPPHFPLHDGSFVSLK

>FvNAC084 FvH4_6g33050.1

MVLSYVLPTEIKEEELEWKKGLPPGVRFVPSQEEFIYYLLKKVYTGKIDFGGVIRDIDLY

KYEPDDLKALAFEGESRLYFFTPLQKKHTKGTRKKRDTVHGLWKGTQTPKTIKDSDNKPV

ITKTPFCYYHPRGKNVKKTQWLMDEYRLINHDNHHIMLENWALCVVHYKKRGGNGNNNEK

EDNKSRSTNKSSSTGSRSEAMGSTFQTSLIGSASECASTMSSQNGNSCQTPTPSKNNTIP

FGYSNSSMGSSSSQPRPNWSYNVNPKKQQQSSTSDQPLLTRGTYNIASHDDHDSSGHALF

PKRTPSGIGSPNCDQTPNCSNTNDLSLNNNVSGDQMLSDFDFMNDSTFSDLYTMLHDESL

QSSLPQQRDDDTQNINPSINPPVHFLLVNVPDEMYQSSK

>FvNAC085 FvH4_6g38750.1

MEEAAKVKLKDGDEELEGGILPGFRFHPTDEELVGFYLPVKVEKKKISTRTSTRLDQLIR

QIDIYKYDPWDLPISTVGDRECYFFCRRGRKYRNSVRPNRVTGSGFWKATGIDKPIYSAE

EPLECIGLKKSLVYYRGSAGKGTKTDWMMNEFRLPPNGKSATNFLNAKDNTEEAEVWTLC

RIFKRIPSYKKYIPNWKQEEATSTKHNPNPTDLNSKTCSFESENCSTHDPYSSLEDVSVV

DIQKINERKPVVVEQVHHGETNNHWFHGSGQFGSLADGHPQEANSFTTSYNGSSFMRNEL

SLRSSETDDFFTDGSCWDELRPVVQLANGCGYIHQVYDDCV

>FvNAC086 FvH4_6g39190.1

MQQASAMEEIRNNQLPGMRFCPMEDELLLSYLKPMLSGQNVPGRNKVVFDCDLYGQQEPW

EIWEAYKTKRPNDLRLHKDLYFFTQHKKMKSTDKRIRRTVGSGTWKGNDSGNPVKSSGRE

IGLKKRFTYENKKSKHHGCWILHEFYLDRSLRDKKEKVKDYVLCLLRKNGDSKTKIEKKR

KQREEEDVLDACDDGEQSNTEQEVLETQAKRQRTVCVPPVPTPSEVDAFIAKIEGFMECV

QDDNSPPSEAEAIGFQGVEDVGLQQLACEVQSSGPSFVPLPEEDFVMEQCFEELRNYEET

EEQDPAGSNNVCGGLIDHNGDDWINSISFSPEDNDFVSCEMLRNFLADTAGLP

>FvNAC087 FvH4_6g39200.1

MQQASAMEEIRNNQLPGMRFCPMEDELLLSYLKPMLSGQNVPGRNKVVFDCDLYGQQEPW

EIWEAYKTKRPNDLRLHKDLYFFTQHKKMKSTDKRIRRTVGSGTWKGNDSGNPVKSSGRE

IGLKKRFTYENKKSKHHGCWILHEFYLDRSLRDKKEKVKDYVLCLLRKNGDSKTKIEKKR

KQREEEDVLDACDDGEQSNTEQEELLETQAKRQRTVCAPPVPTLSEVDAFVAELEGSMEC

VQDDNSPPSEADAIGFQGVEDGGLQQLALEVQSGPSFVPLPEEDFLMEQCFEELSNYEER

EEQDPAGSNNVCGPPVPTPSEVDAIFIAELEGSMECVQNDFSRPSKAEAIGFQGVEDGGL

QQLGCEVQSGPSFVPLPEEDFLMEQCFEELRNYEETEEQDPAGSNNICGGLIDHNSDDWI

NSISFSPEDNDFVSCEMLSSFFADTAGLP

>FvNAC089 FvH4_6g39600.1

MQQASAMEEIRNNQLPGVRFCPMEDELLLFYLKPMLSGQNVLGRNKVVFDCDLYGQQEPW

EIWEAYKTKRPNDLRLHKDLYFFTQHKKMKSTDKRIRRTVGSGTWKGNDSGKPVKSSGRK

IGLKKRFTYGNEKSKHHGCWILHEFYLDPSLRDKKEKVKDYVLCVLRKNDESKSKIEKKR

KQREEEDFLDENDACDDGEQSNMEQEGLLETQAKRQRTVCPVPTPSEVDAFIASLEGSME

CVQDDNSPPSEAEAIGFQGVEDGGLQQLALEVQSGPSFVPLPEEDFLMEQCFEELRNYEE

REEQDPAGSNNVCGPPVPTPSEVDAIFIAELEGSMECVQNDFSRPSKAEAIGFQGVEDGG

IQQLACELQSGPSFVPLPEEAFLMEQCFEELRNYEETEEQDPAGSNNICGGLIDHNSDDW

INSISFSPEDNDFVSCEMLSSFFADTAGLP

>FvNAC088 FvH4_6g39580.1

MEEIRNNQLPGERFCPLEDELLLFYLKPMLSGQNVLGRNSVVFDCDLYGPQEPWEIWEAY

KTRRPNDLRLNKDLYFFTQHKKMSSTDKCIRRTVGSGTWKGNESEKPVKSFETGRVIGSE

KKFTYENEESDHHGCWILFEFYLDRSLRDKKEKVKDYILCLLRKNGEPKTKIEKKRKQRE

EEEVLENNDECDDGEQSNTEKEELLEPQAKRQRNVSAPPVPMPSEDDAFVAELEESLECV

QDDNSPPLEAEAIGFQGMEDGGLQQLACEVQSGPPFVPLPEDDFVLDEDLFEEIYNYVET

EEQDPAGSNNLCEGLMDFEDGDGLVWINSISFSPMSSEEDDAFVAELEESLEYVQDDNSP

SSEAELIGYQGAENGGLQQLACEVQSDPSFVPLPEDDFVLDEKFFEEIYNYVETEEQDPA

GSNNLCEG

>FvNAC090 FvH4_6g46670.1

MAAPPFPGLRFYQPTDDEIIRNYLVPKILGTPKPYDTITTEDVYSTSPEHLPLEENVDHF

NDNEWYFYTTRTNKNHYLTRNGYYLTTSMDEQIFRNNKVVGFKRILRFYLGSRPETGIKT

EWSVHEFQANPGVFPAANLLGNVVQEKLSSLVVCKILRTQEPSVNESEERGRDEGDSSGV

DMEELPSTSKNLKGTKLPQEE

>FvNAC093 FvH4_6g48740.1

MGEKNDQMDKVDDVMLPGFRFHPTDEELVGFYLKRKIQQRLLPIELIKQVDIYKYDPWDL

PKLASTGEKEWYFYCPRDRKYRNSARPNRVTGAGFWKATGTDRPIYSSEGSKCIGLKKSL

VFYRGRAAKGIKTDWMMHEFRLPSIADSSPPKKLLDKSLPPNDAWAICRIFKKTNSMAQR

ALSQSWLSPLPEPTSSGFLTQCPQFSSENNISCTTDLGSAIQFWSNNNDLQHQQASTYSA

FALSNKPNFNPALVGENIPNGFMFTPIDMSSPPNFNHAFVGDLSNNNTSGSIDFDGSQQQ

QFSGFSTSLAQDQNQENTITSLANNTNSHWGTIRSIGFPFSLGSDDPSDMSTSYSTNKCY

T

>FvNAC092 FvH4_6g48120.1

MENVSVFINEDEQMELPPGFRFHPTDEELISHYLSPKVLDSVFTARAIGEVDLNKCEPWD

LPWRAKMGEKEWYFFCVRDRKYPTGLRTNRATEAGYWKATGKDKEIYKAKVLVGMKKTLV

FYKGRAPKGEKTNWVMHEYRLEGKYSAYNLPKTAKNEWVICRIFQKCSGGKKTHISGLVR

ESPYGNDQFRPSLLPPLMDSPSYNNSDTRTTTVGCETSHVSCFSDPMEDQKAQDDIIDSF

NNNNNMSSTSNNHLLASSSSFPFKSSVQNSHFSNQIPPNTGNMQYPDSGFMQDQSIMRLL

TEPQAPSLKRNSSNYLGGQDVPSYSAAPMDFDSIWNY

>FvNAC091 FvH4_6g47070.1

MAAPPVPGLRFQPSDEEIVRVYLIPKILGRLNPYDVVTTEDVYSTSPEQLPLGNDAGHFI

DNEWYFYTTRPISSHYRTRNGFYFATSMNEPILHNNNVVGFRRFLRFYLGSRPETGIETE

WTVHEFRANPAVFPAANLLGSVVQEKLSSMVMCKIVRIQELSDNSSEEGVKDESGSSRIN

LLQNTRLNLQSSL

>FvNAC094 FvH4_6g51660.1

MARAWLFNSRGFASKVKNCSSQVQDCGANRECPRCHYLISNSDVSTKWPGFPTGVKFDPS

DSELIDHLAAKVGVGNSKPHMFIDEFIPTIDWDVGICYTHPEKLPGAKKDGSSFHFFHKT

LNAYSTGQRKRRKIDKEQGLDAQAVRWHKTGKTKAIKGGVHPGWKKIMVLYKSSNKGSKA

DKADWVMHQYHLGSTEDEKEGEYVVSKIFYQQPKQTDKNEDNLVKGEPESSILQISPITP

ITCPPTTPAPQESAWRDDDPDEYIHKSPAKVTEYVPEVSQYAVLRKFEGSMSHVPQLDSQ

FEDNIGQPTWLAGESQADEYYGLNCVDDTLLCKETFNSSTSLNGPVLNPISITDYANNTN

GVTGNGSTSFGVPDLENLEFDSPPDFQLHGLQFCSQDSILDWLDWIGNMQASDRFNINSQ

LEHLQAKYVGTGHADLHRYEWAVNIQRDSYASYVGHHPILSYFALAENESIGRERYNFMQ

RMIKPCGPPPEREDD

>FvNAC095 FvH4_6g54000.1

MNGGGGGGAKMLPVGYRFDPTDEELVVYYLKRKVHGLPLPASIIPDYNSNYYLAHPSHLP

MPLGKTMQKRYFFYNSKDNYQSDNNRSNYKRAAAAGCGFWRSIGKEKQIVDTTCSDQCAC

SQAQLAAVLAVRKTLVFFGSHGRKKKTTHHHRYRYQHQNGSLSTSPRWFMHEYHLRGSEV

LGNNWVLCSVFQKRRKLTSKKRGRTSALDNIGSCGHLPKPSNMFD

>FvNAC007 FvH4_2g00240.1

MATSSGGVPPGFRFHPTDEELLHYYLKKKVSFQKFDMEVIREVDLNKMEPWELQGVCAFV

SVTRKDRVHANYGDCVHGLKKNEASGKEKQDMKKERIELFERCRIGSTPQNEWYFFSHKD

RKYPTGSRTNRATNAGFWKATGRDKCIRNTYKKIGMRKTLVFYRGRAPHGQKTDWIMHEY

RLEDGDDPQGNLSSEDGWVICRVFKKKNLFKVTGNNEGGSSSMNSSDHHHHQQLTMNTNQ

LQARTFMHAHSSRSDNQYLLRQQAFELNRPHDHDQDQDQLPPSLHYAHLNLQPPPQYSLF

HSQALNIPTTNSHKTLAAYNDYSALPSDSSPGHGMVKQLMTNPRDCESTGSESLRYQACE

PGLEVNDSACEPNQQTMVNAGGRSSDDQGMSEWAMLDRLVTSHLGNEDSTSKGGASARYD

QDGNGSSTVNQINQQQQQQQLSLRGEMDFWGYAK

>FvNAC008 FvH4_2g12690.1

MVSEDGLVSAFCDGHFWQPGFRFHPTDEELVLYYLKRKICKKKLKLNVIAETDVYKWDPE

ELPGLSILKTGDRQWFFFSPRDRKYPNGGGRSNRATRHGYWKATGKDRNITCYNRSVGLK

KTLVFYKGRAPTGERTDWVMHEYTLDEEELKRCQNVQEYYALYKVFKKSGPGPKNGEQYG

APFREEDWADDECPIINSSADRGISVEQIGEVVANYNAKPFGEVPLKQVEQITANGYANN

NSEAHSTIDDLEEFMKQIADDAVLDLPQIDDLVHSLSQVVSEEETQSTVVDLYSSEVKCP

QSHTTFDPSGQQGNEYASFDFTQSATSQTQLYEASEASASDVHKLESFTPILREEDFLEM

DDLLGPEPTVSNTGNSGGNLQFNELDGFDLFQDPAMFFNEMCPMDQGNVSHQFMNSMGSN

VVNQFDYQIQPAPLQINHQLNQESTQINNQLWVQNERRDFYTSTETTKGSGPPFTSGVVH

DSSNHCPQENPNQNGNEAAGVTSQFSSALWSFVESIPTTPASASENALVSRAFERMSSFS

RMRIIARSTNVPAGKDSETMRESWKRGFFFLPVLVALCAIFWVLMASLGQWGRCISS

>fvNAC010 FvH4_2g13330.1

MGKSSLPPGFRFNPTDVELVQYYLKRKIMGKKLHVKVVAEVDIYKWAPWDLPDKSCWRSG

DLKWYFFCPREKKYASGGRVNRATDCGYWKTTGKDRSVLYNSEVVGWIKTLIFHGGRAPR

GERTNWVLHEYRLEDKNLAEKGVAQDSHVLCLIFQKDGIGPKSSKQYGAPFKEEDWTDDE

VETDISHVNISETNLVHTNMLEPEPNLTHSYTPEPDIVQANMHDQVLVNTNITEPHLVYG

NMPDPYVVYPNMPQSDFSYANMSEPYLALPSNHNSSITRTNSHESCVSDILPPSGNVLQS

VCSNYETTDHVSPDEDILSMLNCFTEDGTFSMDHIDKNEKLETLIYSGDANPIAGVGKHD

DTNKDLGGLSKDGYIFPNGAISGASSENEPYLELFDLDIEW

>FvNAC009 FvH4_2g13320.1

MGKAAFPPGFRFSPTDVELVKYYLKRKVMGKRVRVQVIADVDIYKYAPWDLPDKSCWRYG

DLKWYFFCPREMKYATGARVNRATGAGFWKMSGKDRSVNYNGQVVGWIKTLVFHIGRPPR

GERTDWVLHEYRLEDKDLADMGMPQESYVLCVIFQKEGAGPKLSAQYGAPFKEEDWTDDE

VEICLDTVPHANRPELDLVLPRGYNSPGPYSTDSPEGMCIGPSSESCISDAVPHSCNVLH

SVSSNSATTEKLCVSANGDILSMLDCFAEESIFPINGNDKNKELDNLIRSGNTSTTQKLD

ILNSRNVDATEELDNILHSGNANATADFHRSDIYRDLADLGNLGRVEVGFNLSNEHDSLY

TMDEIFELGDLDRPLNSDTSMYNLPSLHTELEEMPL

>FvNAC011 FvH4_2g16180.1

MASEMTPELELPGFRFHPTEEELLEFYLKSVVFGKRLRFDIIEFLNIYRHDPWDLPGLSK

IGEREWYFFVPRDRKHGSGGRPNRTTETGFWKATGSDRKIVSLSDPKRIIGLRKTLVFYK

GRAPRGTKTDWVMNEYRLPDHCKLLKDIVLCKIYRKATSLKVLEQRAAMEEDMKMGNSPN

ISSSPPTSMDTTISFSSFQEQELAQHLQHHHHDLTPQTPIMPANLKKELADDAAVAVAEE

QLKEENEQAVEMKESPTSLQTPFRKNKLPELIVPKTSMDWTQDQFWAPMNSPWLLSTPAY

ASLLNLELL

>FvNAC012 FvH4_2g18690.1

MASSSCVHGFNLPSGFKFLPTDQELLGYYLLNKVLGKPFKYDSRVMIELNLYKAEPWDLW

NRFGGPRLNQGEDLYIFTELKKVSNNGSNVARTTGSGTWKGDTSGSEVYVSEDNKKILGS

WKRFHYKPNEPNENGSWIMVEYKLDDSLIPKSCKGSDLVLCRIRKDRKRKLIEDQMNDSS

QSSRRKVPRVDNQDGMSTHGKHILNDDTNLLASFTQFLGSEEEQKPQQQQVDSNMRFEEH

ELQQQDPQVYVSPSPSYFHEQQQQLDTNPVMVSSDHYILQSVKTYEPMDVQPSYFHVQQQ

HLPGDDLMGFSHHEKFRRVENYESMSIVNAHHLENPPLQDGVPFDNLFTVDELYNEASAA

TPVASEPQPTNFYCAANGNVLSYNELDDNVFREAYHPEQNNDAIAAAVDESQFDDGSWLA

DLMDDNNSSEYPAAK

>FvNAC013 FvH4_2g18880.1

MIELNLYKEEPWDLWNRFGARLNHGEDLYIFTELKKKSNNGSNVSRTTGSGTWKGDTAEA

KVSEDKKILGSWKRFRYVPNEPNETGSWIMVEYKLDDSLNPKSCRGRDLVLSRIRKDRKR

KLIEDQMNDTIQCPCTKVARVEETNDVKSTYGIRPILNDDTNLPASFTQCLASEEEQQLQ

QQEELDSNKMLEKQQDPQLLPSPSHFHKQQ

>FvNAC014 FvH4_2g21280.1

MGLRDIGATLPPGFRFYPSDEELVCHYLYKKVTNEEALKGTLVEIDLHTCEPWQLPEVAK

LNANEWYFFSFRDRKYATGFRTNRATTTGYWKATGKDRMVVDPGTGEIVGMRKTLVFYRN

RAPNGIKTGWIMHEFRLETPHMPPKEDWVLCRVFHKGKGEMNSNMSLSPHDLMLESNKSC

TVTLTSPPSHHEQNMHCGYSNQITSFSTTPVPHQSHSHSHNNQSSSLLNLLQLSQEKLTD

SVTEISAKNDDDYGFLWDMNLEESSFENGTLDDMRFEMENSMVLL

>FvNAC015 FvH4_2g27430.1

MSSELQLPPGFRFHPTDEELVKHYLCRKCLSQPISVPIIAEIDLYKYDPWELPGLALYGE

KEWYFFSPRDRKYPNGSRPNRAAGSGYWKATGADKPIGLPKPVGIKKALVFYAGKAPRGE

KTNWIMHEYRLADVDRTPRKKNSLRLDDWVLCRIYNKKGTAGKVQPPMSQQAISGGSEFE

DVKPKNLMACALPPPAAVAPTRPSEYVYLENSDSVPRLHTDSSCSEHVVSPEFTSEVQSE

PKWKEWERALDFPYNYEDATMGNGFGAQFQGGNQMSPLQDIFMFLQKPY

>FvNAC016 FvH4_2g34080.1

MGDAKATSFYLPPGCRFYPSEEQLLCYYLSRKNTAASDESDPGDNGYDLIKELDLYDRDP

FDLPDYACYSYGRGGRRRHWFCYTVRVLKERRARSGYWKRKGRVRDVVGGGGGGGKAVLG

RRSSFVFYLGNSPKTAVRTDWVLYQYAQVDHLQASFVLCRVFVRSQGGTNISENGLSSCA

EETVSTVRHIGIQHDGFYTPHIVPEIDGDKSVPRNTDKSKDQKRPETEVDKQVETRLVSS

IQTNGQVPLLSVSNPVLLDGLSSEHLLSLIEGDFIELDDLID

>FvNAC017 FvH4_2g36350.1

MSTGDHANKEDNLPPGFRFHPTDEELITYYLMNKISDATFTGRAIADVDLNKCEPWELPA

KAKMGEKEWYFFSLRDRKYPTGVRTNRATNTGYWKTTGKDKEIFNSATSELVGMKKTLVF

YRGRAPRGEKSNWVMHEYRIHSKSSFRPSKDEWVVCRVFQKSAGVKKYPPSNQSSSSSRG

ANPYNMELMGPSVNVVQSPIMQLGHEHQFPNYGRSYMTNAEMAELSRVLRGGGVATGSTS

SPMNLPLLQPQFSYPNLGGGGFTISGLNLNLGGPSTQSNLRPMSGIHQAHQAMNNHQDVA

NSSMMTSAGGAVGQDQAGYGNIDMNNGNVPAGNRYMNMVDQCVDLDHYWPSY

>FvNAC018 FvH4_2g40240.1

MRPQAVLPADIGVHCTDKELCMFLQKLTSGSPLPRNVINDVNPYNYMPSNLPPGSFWYLI

RSNESKDTSLGYWSPTEEACKIFTDSVITGWRNTLEFYEGQAPHEYKTNWRMQEYRITYH

KVSENSKTKEANLLCRVQSVGAECKNPIANCFSQPVVSLAETCTGHASTSKPQVIKDNET

GTLTVTERLPDHQVEIMPEIEIDFPFGDDYIELRDLDRPASFSSSSNSSCLTMSSDECFD

SLALLEELEPKTSQDQANKNAGCKYSISVSQKPDELVMYAAASSSGSLNKSPNEEKVKSH

SSKIPSSAVCVKNKEKTTKKLSRNEMPDQGNEAISNSHTATPPGGKKATVVKTKKLKKRY

LCFMPC

>FvNAC020 FvH4_3g03580.1

NKCEPWQLPEKAKMGEKEWDMFSLRDQKYPTGLRANRATKAGYWKDTGKDREIYSSKSCS

LIGMKKTLVFYHGRAPKGVRSNWVMHEYRLEDLFASQLCMQEWNQWILSRVFQKAMKINE

PECKETRMIGSGGYSAEPSSSSSSSISLPPLFDSSPYYPTNASLTDRDSCSHDNRTPMEH

VSCFSTNTGTSDDLMTSLDLTPLPQHQTSSLHVHGFGGLSDFPSSLGYLEENLQLPFLFS

QGMQPHQPYYSSTTGSFEFDLGDWSSLGNWCAATAEEPDRGLGSRRG

>FvNAC021 FvH4_3g04630.1

MESTDSSSGSGHPQLPPGFRFHPTDEELVVHYLKKKAASVPLPVTIIAEIDLYKFDPWEL

PSKATFGEQEWYFFSPRDRKYPNGARPNRAATSGYWKATGTDKPILTSNGSQKVGVKKAL

VFYGGKPPKGIKTNWIMHEYRLINDENSSAALKPPDPTNKKASLRLDDWVLCRIYKKNNA

QRPMMECDHHLHKEDSTTNSMEGIFVQSMTKPSPPKAATNYTALLENDEEDNFFDGILSS

EGVQNSSHSNLSHQFMSSSKSDVVVMPVNNNNSKHQLPSPFWNEAGGSSSMGTNNSLSSK

RFHTDLNSGGGSGGGVDENNASFVSMLNQNAAFHHNSLLGSLGDGVLRPHFHLPSMNWNS

>FvNAC022 FvH4_3g08490.1

MTWHSDEEEEEERAAVQSITPSSATYPQQSKNNKNNEISSCPSCGHPIDFQDQAGIHDLP

GLPAGVKFDPTDQEILQHLEAKVLTDTRKLHPLIDEFIPTLDGENGICSTHPEKLPGVNK

EGQIRHFFHRPSKAYTTGTRKRRKVHTEEDGSETRWHKTGKTRPVLANGAVKGFKKILVL

YTNYGRQRKPEKTNWVMHQYHLGNNEEEKDGELVLSKVFYQTQPRQCGTGTPSIRDGGPP

LLNPFDSHHYKGQVNIRSGQDGIPLPLPPKKAGVMEYYNHNHPGPFMNYEHPHLIQGGQN

REIPAQLIPNMVLQGDGSSLFRYNAADTSKGN

>FvNAC023 FvH4_3g11860.1

MEVQKMMMMRDLSNNSTKDDADYLPGYRFHPTDEELVRFYLRRKVENKPIRLELIKLLDI

YKYDPWDLPKASGLVGEKEWYFFCRRGKKYRNSIRPNRVTKSGFWKATGIDKPVYSVGGE

FQTCIGLKKSLVYYRGSAGKGTKTDWMMHEFRLPATNPSTGAHSNIKDITEEAEVWTLCR

ILKRNNSSRKYETNWHKTTSKQSAADSSSKTCSTESEESFGASAAQKSDPLERSSTPRFN

NDHLCDGNNQLSGGQLMTLKDEVVDPMVNSCINFWNPNEYEMLRPDNPNWDELSPMIDCV

LNPSPLYRSV

>FvNAC024 FvH4_3g13550.1

MMSAGNGQLTVPPGFRFHPTDEELLYYYLRKKVSYEAIDLDVIREVDLNKLEPWDLKEKC

RIGSGPQNEWYFFSHKDKKYPTGTRTNRATTAGFWKATGRDKAIHLSDSKRIGMRKTLVF

YTGRAPHGQKTDWIMHEYRLEDHHDNVNVHDVQEDGWVVCRVFKKKNHINRGFQPEFGHH

QEVEHDLITHMKASGSHGQVILDHHHHQLKQNHQHHLETSLYDYPNTFDGSMHLPQLFSP

ESAAAAAAANNSSSFLSAPNTNMPALNPLDLECSQNLLKLTSTTAGSCGLNMQQQQQQQE

MRSFNGDWSFLDKLLQSHHPQPQDHHHVAAAAATTSTTQRLFPFQYLGCSGSTTHVDILK

FSK

>FvNAC025 FvH4_3g13980.1

MAPMTLPPGFRFHPTDEELVAYYLDRKINGRTIELEIIPEVDLYKCEPWDLPDKSFLPSK

DMEWYFYSPRDRKYPNGSRTNRATRAGYWKATGKDRAVNSNRRAVGMKKTLVYYKGRAPH

GIRTNWVMHEYRLTDSVCGSPSSSLKDSYALCRIFKKTIQIPKNNKEEKTIGNTLLNRNI

LGKDNSTGINKGEASRGIEADQDYENYSNPDYPKFLSDTSSSDLTQGTPTETGIADDLQA

PFASDEANSSANLYSLGAHGSSDNLFQDTYMPNDTSFQNYQFPYPPLELEDFPMINLAVE

MNTLKPQIMDDYISCDKLKDYMNGTLEEIFSLCSSQENNHVALPMQD

>FvNAC026 FvH4_3g15610.1

MDGKITTCKDDHHDYHQDHDDEDVTLPGFRFHPTDEELVGFYLRRKIQKKPISLELIKSI

DIYKHDPWDLPKATTTAGDKEWYFFCRRGRKYKNSIRPNRVTGSGFWKATGIDKPIHDSN

INCIGLKKTLVYYRGSAGKGTKTDWMMHEFRFPSANDVNNNVHTIRTSCTAISQSANLQE

AEIWTLCRIFKRNVSHKKYTPDWRELSAKQSSSKKCNMTTDQDLNQESRNINREAYIDFG

ASNICYEEKKPNVLNHTKYNNASNINNQTLQNAGHQFISGTTTTTATHPYNMDSFTSSYS

YADIENHDFFNENWEEFRSVIQLALDPSFM

>FvNAC028 FvH4_3g16910.1

MAVPMQGVYHVPVGFRFKPTEEELLCYNLRRKIKGLRLPQGVVHHCNVFGTKEPWDIWEA

YRDPSDPETKDLYFFTDKPKMVFSCSTIRRVDTGKWRGKKTRTKVHASGSDRVIGWKRRF

VYKNCDSVQHDCWVINELELHESLLHSKDKQNTYALCILTKMDKKGLTSY

>FvNAC027 FvH4_3g16040.1

MFYMAENACVGGKEWYFYSQRDRKYATGLRTNRATATGYWKATGKDRPIFRKGTTLVGMR

KTLVFYQGRAPKGRKSDWVMHEFRLEGPFGPPKISPGKEDWVLCRVFYKNRELIAAKPSM

GSSISCYEDDTGSSSHHLPALMDSYISFDGPTPQLHHDNEYLRHHQQVPCFSIDTLFSQN

QMTTNPNFTTTSYGNILEQNAGCGGELSNAGTSTFLDNANFSSDNKKVLKAVLSQLTKME

TSCYPNTPSSVFKGSSPSSFGGEFEAAGSSSDNYLSDHQVGMPSVWSHY

>FvNAC031 FvH4_3g18930.1

MEDELLIFYLEPKVNGKKVPGNEDVICELDLYGDEDPWKIWERFGREKANDLRRNKDLYF

FTQKKKKTARSSRASRTVGNGGTWKGQNRGRKVFLLDQNQKPTSTVLGSKKTYTYKNEGS

VHHGRWIMYEYVLDESQIQNKKVNKNEYVLCLLRKNDVLPEKKRKRQAEEEDEMIEDSVK

DDDGDNNYSEPVNEAPQEKRQRLLPSVEDEQERFLVGDQEFLQPEPSLEYGQEAVPPSLE

GESLCFQMDENMGLQTPPMLDEAELQQLFNEEFLPGGQIPLEEEPLDAMQEFESCGQQQL

VAEPEPQPREEDLGQQCQQVPTFSANEIAGGIGFSQDENMVQQMGAEPLGGNWGDAIDGE

SWSKSFMEDLVNYEPPPTMEVGDSNAFYSDFGY

>FvNAC032 FvH4_3g18940.1

MESGDLPGQRFCPMEDELLMFYLQPKVNGLEVPGNEDLIGEVDLYGDEDPWKIWKRFEAT

KANDLRMNKDLYFFTQKKKKTARSSRVSRTVGSGGTWKGQNRAEVYLVDENQKPTSTLLG

FKKTYTYKNEGSVHHGRWIMYEYELDESQILHEKVNKNEYVLCLLRKNDVLPEKKRKRQE

EDEMVEDYVVDDDGDNNNSELMMMQQEKRQRRLPPSVQDEQEQFLVSDELFLQPAEPSSE

YGQVAGAVPTPLEGEPLSLDENMGLLTPPMLEEAELQQLFNGEFLQGGQIPLEEEPVDAM

QVFENCGQQQLEAEPQGPQPREENLGQQCQVPTFSANEIVGDVGFDQAENMVRQMGAEAL

GEVDLYGGNGGDAIVGESWSMSFMESLMNDELPTTVEVGDSNAAYSDFGYNLEF

>FvNAC030 FvH4_3g18460.1

MEGGCERHTLLPGQRFCPMEDELLMHYLNPKVNGKEVPGQETLISELDLYGDEEPWNTWK

RFEKDRANDLRRNKDLYFFTRLKKVSAKGSRICRKVGKGTWKGQDKAKKIYLVDQKQQQT

KTLLGSRKTYTYKNTGSEHHGRWIMYEYELDESQFLHKKQVNKNEYVLCLIRKNDILPEK

KRKRQEEEDDQVLEDYAEDDDDGDNMKPEPVIEEPQEKRQRDLPCTDNVPAPSLDPVAEV

DQWFNSEYYQPAPPLEAYNLAEPQPSEGYLGKQCQMTMYSDNNIMTGHNNFHQDKNQVQQ

RGAEEGFMTPGEVGLYGEDLNYVMDDANWPSYDIEELKQLLHNDQQQLSAMDVGDQSNDN

SYLDFNNLTI

>FvNAC029 FvH4_3g18170.1

MEIHEDGSDSHLLPGYRFCPMEEELILSYLYPKVKGEEVPGEEYLIFNSDLYGKNDPWEI

WNMYKERRKNDLRFSQDLYFFTQRKKRTVNGSRKGRTVGSGTWKAVSSDDVSAGGTVVGH

RSRFSYENKESVEHGRWIMLEYELERSQVSMLNQQDVHKYVLCKLRKNKGSKEKGKRVDE

DDQEKLATGDDMPDDFPALEEYKNVKQEESEIKQGVEDQGMHADGDDVCDAGPSLSSELQ

EAENCWKQYLNPIIFVDDDNLVPPYADALQWEESNITRFGE

>FvNAC019 FvH4_3g03540.1

MGEKEWYMFSLRDQKYPTGLRANRATKAGYWKATGKDREIYSSKSCSLIGMKKTLVFYHG

RAPKGVRSNWVMHEYRLEGLFASQEWNQWILSRVFQKAMKINEPECKETRMIGSGGYSAE

PSSSSSSSVSLPPLFDSSPYYPTNASLTDRDSCSHDNRTPMEHVSCFSTNSGTSDHLMTS

LDLAPLPQHQTSSLHGFGGLSDFPSSLGSLEENLQLPFLFSQGMQPHQPYYSSTTGSFEF

DLGDWSSLGNWRAATAEEPEALAPAEADYMWTY

>FvNAC033 FvH4_3g19410.1

MNLGRHFDQLGGGHVHDNQLPPGFRFHPSDEELISYYLLKKVMDSNFTCRAIAEVDLNKC

EPWQLPEKAKLGEKEWYFFSLRDRKYPTGLRTNRATEAGYWKATGKDREIYSSRTCSLLG

MKKTLVFYRGRAPKGVKSNWVMHEYRLEGRFSYNYLPRNSQEEWVISRVFEKAKKSNGPD

CKKTRMIEPSSPSSSSVSQLPPLFDSSPYYPTAPFSNDASLTDRDSCSHDNRTPREHVSC

FSTGSDPANTGTFNHLMTSLDLAPPPQPQTTSLHLSGGFGGLSDFPSLRSLQENLQLPFF

FQQGMQPDQPYSNTAGSFDLGDWSSLGNNWRAAAAEEPKTLAPAEADCMWTY

>FvNAC035 FvH4_3g20700.1

MESTDSSSGSQPPPQPNLPPGFRFHPTDEELVVHYLKKKASSAPLPVAIIAEVDLYKFDP

WQLPEKATFGEQEWYFFSPRDRKYPNGARPNRAATSGYWKATGTDKPVLSTTDEGGGGTR

KVGVKKALVFYRGKPPKGIKTNWIMHEYRIADNNTSNKPPPGCHDLGNKKNSLRLDDWVL

CRIYKKNNTHRPMDLEDSMDGTMGSSFPLSKLHHLPPKSTTSTYGQFMDNDHNFYDGMVS

SEGINTSASFLPNSALANSSLPLKRELPNLYWNHDPEDEAGPSRRLHMDSSDQSTGNGSI

AILLSQLPQTPPPLHQQPMLGTSQLGGDALFRSTQYQLPGNWYS

>FvNAC034 FvH4_3g20690.1

MGVPETDPLSQLSLPPGFRFYPTDEELLVQYLCRKVAGYQFNLQIIAEIDLYKFDPWVLP

SKAIFGEKEWYFFSPRDRKYPNGSRPNRVAGSGYWKATGTDKVITTEGRKVGIKKALVFY

VGKAPKGTKTNWIMHEYRLIEPSRKNGSSKLDEWVLCRIYKKSSSSSAIQKPMMVSNVPS

KEQSCNGSSSECSSQLDDVLEWLPEIEDRFFTLPRINSLKTLPQQQEDTKLSLQNLGNLG

SGNFDWASLAGLNTVPEVPPNTQAQAQAQINYNNDAYVPSIPPLCHVESPPERMRRQAEE

EVQSGLRTQRGENSGLFQQNSSMFTQSMCTPIDTYGLGSRFGMTGPGFSFGSGK

>FvNAC037 FvH4_3g25470.1

MDHQEPTTGFRFYPTEEELVSFYLLNKLESKRQGSIRRVIPDLDIYSTEPWDLPRYAAEL

CRGDTEQWFFFTPRQQREAQGGRPNRTTASGYWKATGSPGYVYSSDNRVIGVKKTMVFYK

GKAPTGRKTKWKMNEYRAIEGAAAAPSAATTRNSPATTTTTNPKLRNEFRLCRVYIVSGS

SRAFDRRPLEASGDTSHRQSDNLLGASTSSSHQATMVAEKTSSSSDTHGSNSGDLADLQA

SETTDWEMNDDLEQPLWEWEQLNLL

>FvNAC038 FvH4_3g25560.1

MEGDRMPLPGQRFCPMEDELLMFYLEPKVNGMPVPGDGDVIYELDLYGDQDPWKIWQRYE

EERANDLRRNKDLYFFTQKKKTTAKSSRVKRTVGTGGTWRGQTAGKDVFLLDENQQPTTT

VLGLKKTYTYRNKGSVHDGRWIMYEFELHKSQLKKQVNKNDYVLCLLRKNDLLQEKKRKR

QQEEESLEDYVEDDEAVNTDPESVSEEEPQEKRQRLLPSLEDEQEQFLQPAPSLEAEQEA

LPTPLEDDPVFLQEGEFMEWQESDFASLEELLQDESEARPTALEAEQVSLLVDENSGLQQ

LEAEPQLGEDNLEQQLDAVPLFAAQKISGGIQFHPDENTVQQVGGEANMYTENLGDAMHG

ENWHMSFAEELMNGEQPNTMEEGERNAVYPGFDEQVSLRVDENSGLKQLQAEPQLGEKNL

EQQFDAVPLFAAQENCFDSMSTIRELERRSKLPLICIGKSQGIIVFSMELEELCEEWKKL

VRNLELPVV

>FvNAC036 FvH4_3g25200.1

MVTLRPRQGSNNKKQGSSVSETTGTPRSRQGSQNKKQDSKKRSSSTNESGLPVGLRFNPT

DHELVDYYLQSRIALGDSFHSDFVSECPNFYGENEPWVVWQMYGGGESKKEENKTLYFFT

HRKRLSPTSTRFDRKVGSGTWSGQYPRDVFDEEDGSVVIGIKREFRYEEGCDDRHNKAWL

MQEYQIVDDDSDIVLCALRKNHRTPQPAQSSSVVGKTRVDVKKRKTIVDKPKAKRHKKKE

IFDEEEDGSSVDDLSMEPQGTSVDIDQPKMEQINFELDQDHPMLQVVSPFGQLHDDQDCY

YPELLVDDNPDTSTIDDLLAQTPTRYDNNNFNLVSGGSVQDESDKFLDLLLNS

>FvNAC039 FvH4_3g28570.1

MEPPVQQSHPHPKASNSVTEATADSGSPSPKPLQKNPHEAAARTHHHVAHNNGASSVDHH

RFSDEEEELNNFNNNQKKQQDAFLSSFPAGYRFNPRDEELVRYYLQKKIMDEPLPPNKIM

EVNLYRYNPETLAGKYDTYGEKEWYFFTPRDRKYRNGQRPNRAAGDGYWKATGADKSIKS

GNIEVGFRKALVFYRGKPPKGKKTNWIMHEYRVPDSRTAKRTGNDMRLDNWVLCRIYKKA

NNKNKIQTRNHAVDPGEEHISMSITDDTEMEMNGQVDDTQTTESEEEYTHTIAHPPLEFD

GAYPTFAYHYTSYPGVAALDNNMLQPVVMSYKDGYANIFPSLQTSGSSSALQCLDQNGKV

PISHHKLHPVDITSKSIVCKEEASDQMYGVQFPNQKFMQSIEGITSNSTVCTDEACSDQQ

YGMQFPSQEYIVHSLDGRAQTKTHSCGKSVRPGDAVDFGPPSVPYLDQTSIQQKIGKPLL

LPSSDGLQYIIEESNDEHDNLFFDCPHFTYSEPFPWP

>FvNAC040 FvH4_3g32720.1

MEMEPVFSASPNFQLPPGFRFHPSDEELIVHYLQRKVTSRPLPAHLITEIDLYKYNPWEL

PKMALFGEDEWYFFSPRERKYPNGERPNRAAASGYWKAAGSDKPILTTCGISKRIGVKKA

LVFYTGRAPTGSKTEWIMIEYRLLDSSIKPSKSKGSMKLDDWVLCRVQQKGNTSKNKCNS

QGSSPEYWSGLQKVEQTQHTYANPNTEMITDFLYKDCTLLASILAGQAPPISSVSFQRTN

NGYEAGPNYKVNSTISISSPGLISETLKRKSTEEITKKNPFRIDNNLASKSNNEDLLPSK

RMEENYISSYNHSHSQNDIVKANPIDTTDYEELNEMAILGRYFSEI

>FvNAC096 FvH4_7g06500.1

MGHELVPSTTTPPLAKPPAAPTALAPGFRFHPTDEELVIYYLKRKVCRKPFKFNAISDVD

IYKSEPWDLACKSSLKSRDQEYYFFSALDKKYGNGARMNRATNMGYWKATGNDRPVKHNN

KTVGMKKTLVFHSGRAPDGKRTNWVMHEYRLVDTEMERPGNGVPQDAYVLCRVFHKSNIG

PPNGHRYAPFVEEEWDDEKLTFVPGEVARDETSAAHDSVGNHRDVVMLGNGHDFVGGNGH

DSVGGNHYNVVMVGNGHDSAVGGNHRDFIMVGNGHHSVVGGNHRDVVMAGNGHDSVVGGN

CRDVVVIGNGRDSVVGGPIPDAVVGANGCHALVFGNGRDTVAGNARDAAVGGNGHAAAFI

EQDVHDAVDIDQKDHVSMVVEQKDNAFASIEQKDHNVCIGGNGHGPSVDGHVQNNKMEVD

GHGTTKEGNVHGSIGGNGLKGATAGNYIVQDTQTISNALLSATELPKEDPTVLVPCKSEK

LDDDPVTCVINREERLDDYPSPLTDDPQPLLSLFNQQPGILRQYKRRRHNESNSNRSNAS

EISSGMTHDPCSSTTTTASTEASVTASTKRNFLSALVEFQLLESLEPKDKTPVAPPEFNA

ALHNSSVPSSCLKFIESLQNEIHKISIEKETLKFEMLSSQAVINILQSRIDLLNKENEEL

KRNAREADYPAVIHNKNHPQDYKLLHGELVWSHTPTYTMSRQHLSSSSSFVSTSI

>FvNAC097 FvH4_7g06570.1

MAYPPLGVGFRFRPTDRELVAYYLHNRAVMGDQFQSSFVFNCPDIYGQTEPWIIWDLFGG

NLIEEGEALYFFTQRKKLNPKAKRFHRKVGSGTWSAQSKDDVVSADHHVIGTKREFRYEK

GSDPLQNGVWLMQEYELTSLNDMVLCTLRKSSRPPAETASSSTAAPPLNQSVDENFGNKM

KRKMNFCEEEEEEDSCKRKKLEPPKPSYLYEQHFDVDKLFDFDHQPKTPDHMVSVAQPFV

QVEKSNDDLIMPSLISDDESLEAVQVMASVGQSICAATINSLMDSPISFEHFDAEVDEFL

GLTADDYNNNNNEDGISQFSSSDLSCLD

>FvNAC098 FvH4_7g11470.1

MDNGNHDDNLLPGQRFVPMDDELLLCYLKPLVHGKEVPGRDFVVFDCDLYGDQEPWEIWK

KYESKRKNDLRKNKDLFFFAQQKKKTPKDSRVGRTVGNGGTWKEESGKKIVSPDTNRVIG

HKKTFGYKNEGSPHEGCWIMHEFELDPSQLIHRKQETNNVLCVLRKKKDPDRETNKRKQQ

TSGRNSVPDDGTNNLISNKQQRLDCEPSTSSARPPPAPLGFGALKDEHEQFWQPPCNSNP

NAQALPLPYAFAGEENRGLQQFAAGDEQYWGQQQFVVPSGGEENRNASEMGENLWQHMAG

HQLGTTPHPHTSSSTIWSGLGVDAPILSLSP

>FvNAC099 FvH4_7g12150.1

MASSLPLGTQITFNPTDSQLMQFLLQDLAPDFIITDCDLFGHEEPWAIWDKYLGFRNQFN

PDELYFFMPALKKLSPNGGSRINRTIGGGSWTQKEPFKIVCADEDEGRAIGVKRKLKYLN

QGSDQNAQWCLDEYTSTVEGHKSCAIFRLRRNERQHKSLSDSTIRYEDFFPPYGITIDQE

EPWEIWERYSAAFNKPDLLYFYASSMKRLSSKGRGCNFDRRVGSGTWNQKEAAKFVYEDV

GEADQERKPKRIGKKRKLRYVNEGSVHHGEWYLEEFTSLVDEGAVLLLRRNERYKAPCLS

ISSDIPKEKKRKRQQIDQLEDKKPKRKKVVRQLDFSDSLNRQGESGTTPTVHIQQQKPDN

VDYVLEFFKDVDTGSWEFPSFDDFVMACPPALEDCDLEALASLEFPSLDEFFTACPPPLE

DCDQEASTSLELPSLVDLTSPSPSAGDQQAIAAVDIDDNFILTGSCNDDDLAKWDFPIEE

NCSLTSLKLPSLDDLTSPSSAGDQQATAAVDIDDNFNLTGSCNDDDLAALDFPIEEYCSL

TSLLQGNCDVDFKKWSMETCFVDFDEQA

>FvNAC100 FvH4_7g17540.1

MTENVSISVNGQSQVPPGFRFHPTEEELLQYYLRKKVSNQRIDLDVIRDVDLNKLEPWDI

QEKCKIGTTPQNDWYFFSHKDKKYPTGTRTNRATAAGFWKATGRDKVISSNCRRIGMRKT

LVFYKGRAPHGQKSDWIMHEYRLDDNNSCITSVSITVVGEAAEEGWVVCRIFKKKNLHKS

LSTPILSSTISSSITTELPRSSSQMFDDDDHEGAFEEMLQYMGTKTCKEEITDHHLHPIH

TGISNHINGYHDHHNDRFLKLPSLESPKSTSSQNYYQPIINNHEDMVIDQNEGTVMNQHR

HYQHVDDSASLTNWAALDRLVASQLNGGQETGSSRQFACLIEPTLNAFCTSTTDNELQLP

STLRSSSSSNSSSKSYHATQQDYYTNEIDLWSTFAAAKLQSSSPLSSSDELYHVSNGHI

>FvNAC101 FvH4_7g17550.1

MTENMSISVNGQSQVPPGFRFHPTEEELLQYYLRKKVSGQRIDLDVIREVDLNKLEPWDV

QEKCNIGTTPQNDWYFFSHKDKKYPTGTRTNRATAAGFWKATGRDKVIRSNCLRIGMRKT

LVFYKGRAPRGQKSDWIMHEYRLDDNTSRITNVSVVVGEADQEGGWVVCRIFKKKNLHRP

MLSATTSSITTDTTRSTHQLLYNSCDADEGALEHELKVMESRTCNGCKEETNEANNSLFM

NHIHGFHDDVDRFFNLPSLQSPDSSAENDDQVSHQRVDWVGLTNWAALDRFVASELDGRE

MMTYPESGNYFITDHHDFHDHELQLPTTL

>fvNAC106 FvH4_7g18040.1

MSCGGFTVPKGYRFHPSDEELLSDLEQKNHCKDSQITTIIPEIDVCKYEPRQLPELAFRG

AENLVGKFFSFFFFFWIIKEESWHSQRKWYFFTLRDYKYTNSSRANRTTGEGFWKITGKD

RAIKAPDSKAVIGRKRTLTFYKRGVSKAQKTSWVMHEYFLIQESCDPPKQIGKYVVCCLK

YKSDNSDHDKDAPFCNRGSSSCSMSFNVENQAEENWVSEELVNVPIPNGNESETVGGMIT

EEEECLAFKELEDALQVPIGDPHEFEGMQTDPRRIQQNDHSTSVESRPDRSDSSDTSDMI

DELLDDPENLDSLFDPPSPPQLDQLHQSPMYTTDVHKNECRKRPYPFEHDDPSLPKKNNI

STDDGNKLVSNSASNSESQAANVIPEGYSQPGANLGSDFDLFLPTDYVCTQPSINGGPRD

FSNDNSFIELDDLPSFYEGIDFSQFSDRVIDGGRGVTSDRNTSVVYGSENDYSALASGAS

LHHS

>FvNAC102 FvH4_7g17950.1

MSYGGFSLPKGFRFHPSDEELLSHYLQMKNDQREDPEITAIIPEIDVSKHEPRDLPALVF

ARAEFLDREWFTMADSPDMEWYFFSPRVFKHSNSKSTKINRTTDEGSWKKQGNDRRITGA

FSDKQIGGKRILTFYLPNKQKTDWVIHEYYLTKADSDEQIGDFVLCRLKDNRLKKKSGKS

EHDPQDAPLCDGREPSSGSCSMAFNVEDQASSELGNVVVIPNGNVDEAEHGGGSCSMTLY

VESPASKQLENVLLNPYGNNDDTEHGHIVSGMLTEAEEYQGFKELQDALFESDGNDQGLR

GSETAPCYIDKNDLSVSVEGQPDSYNSPDSEMVEELLHEPGDLALLHPPPSIQLQSPIYT

KLGTHNIFNDEYRKRKYPFGDSEPFLTKKNHTDEVDSYTSSNSENQPADAIPEGYSQPGG

NLGSDLFRSQTLPSINREPRVLSHDNIWDHLPSIIEGFEFSLANFSDTVIDRGISDRNIE

GAYGSANDYNALAIGETL

>FvNAC105 FvH4_7g18020.1

MSSGGFTAPKGYRYNPSDEELFSDLEQKNHCKDSQITTIIPDIDDVCKYEPRQLPELAFT

RAENLVGKFFFFFFFWIIREESWHSQRKWYFFTRLDYKYTNSSRVNRTTGEGYWKITGKD

RAIKAPDSKAVIGRKRTLTFYKRGVLKAQKNSQKTGWVIHEYFLIQESCDPPKQIGEYVV

CCLKYKSDNSDHDKDAPFCNRGSSSCSMAFNVENQAEENWLSEDLLNVVPIPNGNESETV

GGMITEEEEYLALKELEDALQVPIGNPHEFEGMQTDPCCIEQNDHSTSVEGRPDSSNSSD

MIDELLDDPENLDSLFDPPAPRQLDQLHQSPMYTTDVHKHECRKRPYSFEHDDPSLPKKN

NISTDDGNNVSNNASNSESQATNVIPKEYSQPGANVGSDFDLFLPKDYVCTQPSINGGPR

DFSNDNNYIELDDLLSFYQGIDSSLAEFSDIVIDGGRGVTSDRNTSVVHGSENDYSALAS

GASLHHS

>FvNAC104 FvH4_7g18010.1

MTTNLGTDETMPLSLPVGYRFRPTEEELVNHFLKKKIRGENESEINQIIPFIDLYEHEPA

ELPGLLGSETEDHDMEWFFFTRNAYKYNKSRRSNRSTKKGFWKITGKERGIKARRSKAVI

GKKRTLTFYQGRGKAKKKTDWVFHEYYLPQNQVVSCSKKEKGDFVLCRLKNKSDKKDSSV

RNEGEPGRGDDEMNQEGDGEFLFHQPQTIDDCCSSALWSPASQELEAVLQTNGDCHELQS

PFGESESCLLHRNQVLTCDEDESVYDVYPQLRDPPDENLDSLLRAFQPQDYCPPILQSPI

YTKLGNVTHPLQPQNYQPSVIFQSPIYTKQGNGPDANLHYGIPVVDIPVRHVMSNIENQA

SDYRSLEVPRPQTEVNLESAVYRFQPQDSTLQPLMYTDFGDALHNIECNELQSFFGDDHS

SFGNIAYQDYYSSDQTAQTPFKDSCRNHWEGSTMGIVE

>FvNAC103 FvH4_7g18000.1

MSYGGFSLPNGFRFQPSDEELLNHYLQKKNQREDPEITAIIPEIDVSKHEPRDLPEPFNT

TITGVHRGGVSGQGLREWFTMADSPDMEWYFFSPRVFKHSNSKSTKMNRTRGEGFWKKQG

SDRRITGAYSDKQIGGKRILTFFLPKKQKIDWVIHEYYLTKADSDEQIGDFVLCRLKDNH

LKKKSGKSEHDRQDAPLCDGREPSSGSCIMAFNVEGQASSELGNVVVIPNGNVDEAEHGG

GSCIMTLNVESPASKQLENVSRMVLELILEGGEKCH

>FvNAC111 FvH4_7g18260.1

MSYGGFSLPKGFRFQPSDDELLSHYLQKKNDREDPEITAIIPEIDVSKHEPRDLPALVFT

RADFLDREWFTMPDSPDMEWYFFSPRVFKHSNSKSKSTKINRTTDEGYWKKQGNDRRITG

ACSDKQIGARRILTFYLSNKQKTDWVIHEFYLTKADSDEQIGDFVLCRLKDNRLKKKSGK

PDHDRQDAPLCDEDQAFSELGNAVVIPKGNDDEAKAEHGGSCSMASNVESPCGNDDNAEL

GGGSRHIVPVMLTEAEEYQGFNALQDAVFGSNSNEQEFGGSQTGPCYVDKNDHSVSIEGQ

PDSEMVKELLHKPVDLALPHPPPSTQLQSLIYTKSGTNNSLNDEYHKRKNPFGDSDPFLT

KKNHTDEVDSNTSSNSENQPADPIPKGCSQPGGNLESDLFRSQKPPSIIREPRDLPHDND

FIEWVDLPSITELIEGF

>FvNAC109 FvH4_7g18210.1

MSYGPVPKGLRFMPSDEVLLSHYLQKKNQREDPEITAIIPEIDMTKHEPRDLPALVFARA

ENLNKEWFADSPDMEWYFFSPIVLKHSNSKSKSTKINRTTKEGSWKKQGNDRRITGACSD

KQIGGRRILTFYLSNKQKTDWVIHEFYLTKADSDEQIGDFVLCRLKDNRLKKSGKSDQAF

SCKLGNVVVIPKGNDDEAKAEHGGGSRSMVSNVESPASKQLEDVLPIPYGNDDDAEHGGG

SCLNVPGMLTKAEEYQGFNALQDAVFESNGNEQEFGGSQTGPCYIDKNDRSVSIEGQPDS

EMVKELLHKAVDLALPHPPPSTQLQSPIYTKLGTNNFLDDECHKRKNPFGDSEPFLTKKN

HTDEVDSNTSSNSENQATAAIPEGCSQPGGNLGSDLFMSQTLPSINREPRDLPHDNNFIE

WNDLPSIIEGFELSLANFSHTFIDQGISDDINIEGASAYGWSAI

>FvNAC110 FvH4_7g18250.1

MTTSRDQTMPLSLPVGFKFHPTEEELVNYYLKKKIHGGNESEINQIIPFIDLCEHEPAEL

PGLLGSETEDHDMEWFFFTRNAYKYNKSCRSNRSTKKGFWKITGKERGIKARRSKAVIGK

KRTLTFYQGRGEAKKKKTGWVIHEYYLPRNEVVSCSKQTKGDFVICRLKNKSDKKESSVS

NEGEPGGGDDGMNQEGNGEFLIYQSQPLDDCCSSALWSPASQELEAVLQTNGTSGDCHEM

QSPFGDSESCHRDRNEFSTCDEDETVSDVYPQVRMNMLSVKKFGYNSTGLPPSHTAVTNI

HKAGKCYTSASATELPALGNIAVTNIHKATRLPPSHTAVTNIHKAGKCYTSTSATELPAL

GNIAVTNIHKAGKCSKCQSGECHNWQSESTKTISTDKVDIPVRHVMSNFENQAPDYRSLE

VPHPRTEVNLGSTVYPFQPQDSTVQPLMHTKMGDALHNIECNGLQSSFGDNHFSFTKFLN

INSAYQDYYLSDQTAQTPFKDSCQNHWEGSTMG

>FvNAC108 FvH4_7g18200.1

MTTNRSRDETTPLSLPVGFKFHPTEEELVNYYLKKKIHGGNVSEINQIIPFIELCEHKPA

ELPGLLGTSATEDHDMEWFFFTRNAYKYNKSCRSNRSTKKGFWKITGKERGIKARHSKAV

IGKKRTLTFYQGRGEAKKKKTDWVIHEYYLPRNEVVSCSKQTKGDFVICRLKNKADKKDS

SVSNEGEPGRGDDGMNQEGNGELLIHQPQPLDDCCSSALSSPASQELEAVLQTNGTSGDC

HELQSQLGDSESCLLDRNEVSTCDEDETVSDVYPQLRDPPEENLDSLLCALQPQDYHPPI

LQSPIYTKPGNVAHPLQPQNDQPSVILQSPIYTKPHHPPILQSPIYTKPGNITHPLQPQN

YQPSVILQSPIYTKQGNVSDASLYYGECNNWQSESTKTISTDKVDIPVRHVMSNFENQAP

DYRSPEVPHPQTEVNLGSAVHPFQPQDSTVQPLMYIKFGDALHNIDCNELQSSFGDNNFS

FTKIITRLTKQHRLLSKTPAKITGKGLPWDSGVSTDTNTEVAQGWVQYSSALAIGTTLQC

SSGTTRFLILSCNEIRQSHNSKFLAGILCTEISNCLLMAQIAKTTKSLQDWVWGFGYVPW

LLMQNKVS

>FvNAC107 FvH4_7g18070.1

MEAKASQTVTREPHQEAATVRGTRQNIGGNIYSKHKAKDFGRSLVTVSTVVTEQPQEHSL

FDESWPGMATSASLPVGFRFHPTEEELIDHYLKKKIKGHDFNDVIPEIDICSYEPWDLPG

FSSIRSDDEEWFYFSRPDYKTKGNSRNRATERGFWKITGVEREIKARRSKALIGKKRTLT

FYTGKVRSGQKTGWVIHEYYIPENLLPNAVRQRDFVLCRLKKKDESADNEACGQGEVCQT

STDNLSDFQNLVANLGRPSSPEDLQITLQENTRPEEIFATLFSSHEPPDYFASILGGSFL

HENAGSDDHLDLQSAFRHEDSDEGDFSLEDIPRTNLFNHSSEPLSTLRKVYDEGIHRQKK

ASSVNVATTLPQNIGRVRSVNMEESEYEPKYVLQAGGSSSDTLSSALVTYKSYLNEKGGR

KGEMITGGAELQSGPQLPLRPDDYITQAKVRNMTAKSYQGQIKQGFKPQEVKYPSMVPEK

ALDPDKPWRNDEQKGMKTKQIGTTIIDWKSSFIVWKESPLSLGSCPPVVYIRNMIGGLVL

FIFFVGELISYGKW

>FvNAC112 FvH4_7g27750.1

MNTFSHVPPGFRFHPTDEELVDYYLRKKVTARRIDLDVIKDVDLYKIEPWDLQELCRIGT

DEQSDWYFFSHKDKKYPTGTRTNRATAAGFWKATGRDKAIYAKHDLIGMRKTLVFYKGRA

PNGQKSDWIMHEYRLETDENGAPQEEGWVVCRVFKKKLPSMRRMSEHESPCWYDEHVSFM

PDLDSPNHNNSNSSMNNQMAYHGHFPNYPCKKELDHLPFQHHVPNEHFFQLPLLDSPKLL

LQSSSSAAVSSNSMTAAAAYSIDINQNFHAVYGNNSNELQAVDDDQLTDWRVLDKFVASQ

LSQDDVSKGNSYS

>FvNAC047 FvH4_5g00350.1

MSEAFAAIKPAEKIKERETNCNFLKHKVHRLRRRDQLKSRGLKNMNTFSHVPPGFRFHPT

DEELVDYYLRKKIASKKIDLDVIRDVDLYKIEPWDLQELCKIGSEDQNEWYFFSHKDKKY

PTGTRTNRATKAGFWKATGRDKAIYIRHNLVGMRKTLVFYKGRAPNGQKSDWIMHEYRLE

TNENGPPQARWVVCRVFKKRLATVRRMGDYESPCWYDQSSSFMPELDSPRTNMFPNPAYT

TTSSSSSYHQQLYSNCKQELDHHLQYNAIPVPPHPDSFLQLPQLESPKVAQYGTYDHHLR

NNGCHVMPSSTFRPEEQVQQMNQHNLMINSSPLLYNDNSLDDQLTDWRVLDKFVASQLSH

DQQEASKETTANYSNEAVFDPVGDEVHTSMGANESKRPENIIAQDYAASTSTSSCQIDLW

K

>FvNAC048 FvH4_5g05980.1

MAVPMQGVNHVPVGFRSKPTEEELLCYYLRRKIRGLRLPQGVVHHNCNVYGKKEPWDIWE

AYRDPSDPETKELYFFRDEPRRVFSCSTIRRVDTGNWRGENLRKKVHAYGSDRVIGWRRT

FVYTNRDSVQDGCWLINELELHESLFPSKDKRNSYVLCILTKIDKKGKH

>FvNAC049 FvH4_5g09000.1

MDKFNFVRNGMTRLPPGFRFQPTDEELVFQYLRCKVFSCPLPASIIPEINVCVYDPWDLP

GDLEQERYFFSNKESKYPNGNRTNRVTSSGYWKATGSDKKIVSARRNNIVGKKKSLVFYR

GKAPNGSKTDWVMHEYSLVNNLGTRALSTENSINQQGNWVLCRIFLKKRSSHKDEDNLLN

YDGFKVNNAEKGQLQITRTTPVSSCSSCSSCSGITEVSSSHEAGDEEISGCAH

>FvNAC051 FvH4_5g10690.1

MSAEDQMNLSINGQSQVPPGFRFHPTEEELLHYYLRKKVAYERIDLDVIREVDLNKLEPW

DIQEKCKIGSTPQNDWYFFSHKDKKYPTGTRTNRATAAGFWKATGRDKIIYSGIRRIGLR

KTLVFYKGRAPHGQKSDWIMHEYRLDDSSSSLDTTVSNSIGESMTEDGWVVCRVFKKKNY

QKAMESPKASFSMDSQNNQMLPGSRNDGVLDQILLYMGRNTCKIENHDSLHHERFMHLPR

LESQTSLPSLPVQFDHNRSFKACYHDQTIDDMLIETDQQQQQQQPSPTNQSNDLVHDHHD

HDDPKTRIVNDWATLDRLVASQLNGHEDETSKHLSCFGDPNMAFYSSSPSNDGDLQLSYP

YLRQGRISHNQPEVFNSENDLWSFTKSSPSPSSSDPLCHLSV

>FvNAC050 FvH4_5g10350.1

MAVLSMESLPLGFRFRPTDEELINHYLRLKINGRHSEVQVIPDIDVCKWEPWDLPKLSMI

KSDDQEWFFFCPRDRKYPNGHRSNRATDAGYWKATGKDRTIKSRRFKSAANSTGLVGMKK

TLVFYKGRAPKGERTSWIMHEYRPTQKELDGTAPGQCAYVLCRLFHKPEEKAEVLKYDEV

DQPGLSPTTSPDETSSDVVQESPTPDMKGGKESEGISQWNDSFDHMTPDTVTMPPIDSYM

ASDVEVSGPEETIFSGEMSFFEPTFDCKIFSPSHTPFDAELDYVGSPLEFGNHNNGLYFQ

DGTCEQDVYLPEVFDEVGNKNYESSLEESQKNLVVGSEAYLSDSFMLQDIPPENPWLNGA

WGYPDAKAAQHNLDTGARGLSNEQFDIEDLLRKTSFGAYQAEAPASLYDHKPAMENIGYS

GYSYPDSAVSNILDGSTRLNDVDNHSSDQVGGLKIKIRSRLPQERPNSNFVDQGSANRRL

RLAVRNHTRVSNHNKEEDEGHSTITEAREAGQSTSDEWEKVHAIDDEEEVTSRDYSVNKD

YDLGGKNEISCETNQPPEPSNSGKPVTRGMATRIRLMVNGSPGSVANSNVSDTKYGQEDK

VKSSITEASETREESPRLNEQEVKCHPMKLDANQKIAEEPSTSLVDENKKTIEEAATKTR

SRTGGDDNSLHSGHIGMSVPSKNRHLNTVFIITVGIPLVLTMFVAFSGIWISH

>FvNAC052 FvH4_5g12090.1

MQENLPPGFRFHPTDEELITYYLGRKVSDVSFTSQAVAVVDLNKCEPWDLPGKASMGEKE

WYFFNLRDRKYPTGLRTNRATEAGYWKTTGKDKEILRQGVLVGMKKTLVFYKGRAPRGEK

TNWVMHEYRLENKHHPFKSSASKEEWVVCRVFQKSIALKKPQQMTSSSPQSVDSPGETNS

MVNEFGDVELPNFMNNVANSSSPGFISNMSIPQGNNYNIIDSKMINNDVNMMNTTLNMNN

WLGAREAAASGAILPAAALGSWPSSLLSPSNLSSVNSLLLRALQLRSNSASTNSTIYQQP

IRSDDAAAGLEDHYSSFMEAQQQQQRQPGLMSHFGTDHNMNSSNVQASSSSKDQIMDSMP

QSQQHLEQPFNLDSIW

>FvNAC054 FvH4_5g14170.1

MEAQKGEGEVVMSSDLLLPVGFRFMPTDEELVRYYLMNKACFRAVPVADAIQEIDATRFY

SNHPKNLVTFSNGEREWFFFIHEDDENYSGSCRSHAQRRRNVREVGNGLGFWKPSGSENP

IYSKDGNVFATKIFLTYFSGSCHSKKPKRTHWKMVEYHLQIRREWVVLGKLQRVMDYTGF

>FvNAC053 FvH4_5g14080.1

MEAQKGEGEVVMSSDLLLPVGFRFMPTDEELVRYYLMNKACFRAVPVADAIQEIDATRFY

SNHPKNLVTFSNGEREWFFFIHEDDENYSGSCCSHAQRRRNVREVGNGIGFWKPSGSENP

IYSKDGNVFATKIFLTYFSGSCHSKKPKRTHWKIVEYHLQIRREWVVLGKLQRGMDYTGF

>FvNAC055 FvH4_5g14670.1

MMQEMEKFVLPAGNFKEDNQIDLPPGFRFHPTDEELISHYLHKKVMDSNFGCKAIGDVDL

NKSEPWDLPYKAKMGEKEWYFFCVRDRKYPTGLRTNRATEAGYWKATGKDKEIYKGKSLV

GMKKTLVFYRGRAPKGEKSNWVMHEYRLEGKFSVHNLPKSAKNEWVICRVFEKSAGGKKV

HISGLPNSGSRGTETGSSGLPPLLDSTPYNGAKTRPASESTYVPCFSNQESIDSQRNQGI

VDYINNNNPLFSVSSNPSNNNNNNNNSFPRVTYQYSAPVSSANFQFPSSVVMQDHLRALL

ENNVSFMRQNFKTERDMISVSQETGLTTDVNPEISSVMANHEIGRRPFDDQDSFWM

>FvNAC056 FvH4_5g18130.1

MAPVGLPPGFRFHPTDEELVNYYLKRKIIGQEIELDIIPEVDLYKCEPWELAEKSFLPSR

DPEWYFFGPRDRKYPNGFRTNRATRAGYWKSTGKDRRVSSQNRAIGMKKTLVYYRGRAPQ

GIRTDWVMHEYRLDDKADCLDDTSAGIQDSFALCRVFKKNGICTEIEEQGGQSSSSDVSL

LIHERSSQLLAAGGLHYNDHCSTETLSPENIIPRASSSSCLQDLEEEEKDDSWMQFITDD

PWSSSNIPPVNGTTGEVDLSQSHVGVAFTT

>FvNAC057 FvH4_5g21670.1

MGGASLPPGFRFHPTDEELVGYYLSRKVEGLEFELEVIPVIDLYKFDPWELPEKSFLPRR

DMEWFFFCPRDRKYPNGSRTNRATKAGYWKATGKDRKVVCQSSVTGYRKTLVFYRGRAPL

GDRTDWIMHEYRLNDDFAQGSPGHKGVFALCRVVKKNEHTQKANDCSAEPKAKTVGSTSS

NGDLTSTINSNETLSISAGMSSQVNYQHDESRHSSHTTSPYEVTPMAEFEPAPRDTNPTA

FWVSPDLILDSSKDYPQLQGAVPNYIPQSEFPSTMSPWQSYEHTEISPSSSYSNFGDFQM

ADDLNQMACMSPFSGHTDYMSYCGNEGLQFEVSETWDGQAPICRQVSADGNLGELGGLWL

QEDNMIIVM

>FvNAC058 FvH4_5g25360.1

MEESRGTQLPGQRFCPMDDELVLFYLKPMLSGENVPGRNRLVFDCDLYGKQEPWEIWEDF

KTRRPQDLRLNKDIYFFTQHKTISSTDKRIRRNVGSGTWRSDDFGKPVVSVETGRVVGLK

KRLTYKNVDSVQNGCWILYEFFLDRSLRDKKRKLKDYVLCLLRKNGEPKTKIAKKRKQRE

EEDEVLENNDACDDTEISRGEQEELLEAQAKKQRTAPSIDSAPPTMPSQDDDAFAAELQE

TLECVEDNYAPLLEAEASSFRGDEENFGQQPLAANHGIFNQLQEKDRFEEFLDRLSTEEM

EEMAEILFYGMSSNIDVAEQNVVNSSTLHDAPVSSMSAAYDYGTAIPCLSSENGCIDTSG

CNDVGGGVMGYEDGVCMPEPLVEVAADEQQKTPDDDLSDLLGSIDFSPEANNFFCSEPQL

CS

>FvNAC059 FvH4_5g25510.1

MEERRGNQLPGQRFCPMDDELVLFYLKPMLSGENVPGGNRVVFDCELYGKQEPWEIWEDF

KTRRPHDLRLNKDIYFFTQHKTMSSTDKRIRRNVGSGTWKSDDSGKEVVSVENGRVVGLK

KRLTYKNEDSVQNGCWILYEFFLDRSLRDKKQKMKDYVLCLLRKNGEPKTKIAKKRKQHE

EEEVLEDNDACDDTEMSSGEQEELLEPQAKKQRTAPSTDNAPPTVPSQDDFYSHIIRSEL

>FvNAC060 FvH4_5g25960.1

MLGLMEEMSELLGREEVNEQGLPPGFRFHPTDEELITFYLASKVYNGAFCGVDIAEVDLN

RCEPWELPDVAKMGDKEWYFFSLRDRKYPTGLRTNRATGAGYWKATGKDKEVYSASSGAL

LGMKKTLVFYKGRAPRGQKTKWVMHEYRLDGDFSSYRHTCKDEWVICRTHNKSGDKKNPM

QAQGQGEGQQSNFLQQVVEAASASSRTGCSLPPLLEFQSQTTHQNPSFVQSPSLLRMQEN

DLMKSRNQLFQPPSSSSSSSSSSAAALQSMLFKSFLFPHQDQAGTTGTGTGAPKRCKAEA

TNLPHFQTAPLNVANFNYLMEKNHSHVQPNGQDSLLQYHHHPRSSLSFDDFHFYNGVLGL

STSAATASVDAETASSSASNGGFNRAGFQTMLDPLIKLPAAESSWNQDIA

>FvNAC061 FvH4_5g27330.1

MAIAATRMSHEEDESNKQHHEVEVDHDEHEHDMVMPGFRFHPTEEELIEFYLRRKVEGKR

FNVELITFLDLYRYDPWELPAMAAIGEKEWFFYVPRDRKYRNGDRPNRVTTSGYWKATGA

DRMIRSENFRSIGLKKTLVFYSGKAPKGIRTSWIMNEYRLPHHETERYQKGEISLCRVYK

RPGVEDHPSLPRSLPSSRAAAANANGASTSRSLLQAQSSSSSSLNKKQHSINSSLMEKLQ

ANFNFNGGSSSLGQSVQQLHHHHHQDQVAADPKINETEGSSGNSDVTSTLGVGLRSSATN

IPIHHPSSPLGLQPHHHHHGGVLHLDDHHAEQEGISTFLQQHHHHSKPQTLLFAGSSVSA

TSNVIDDLQRLLNYQQQQAVSNIANQQQALHVQQYYDAHSHNHNHNQQLPSNVVNHFSSP

FPLAPPPPSQSQLSPNGLPVTAFSDRLWDWNPISEPNRAPDQYNTNPFK

>FvNAC063 FvH4_5g27760.1

MGLLPGQRFCPMDDELLLHYLKPMVNGKIVPGKDSLFCDSDLYGNLEPWQIWQEFESRHV

NDLRKNKDMYFFTKNKKLGSRTCRKVGTGSWKGQGAGNPVYLLDENADPTTTLLGLKKAY

TYVNRNSVHHGHWVMYEFQLHSSQITQLTNDSVLCMLRNNDELPKRKRHQEEEGLEEQSV

EVSAGDSHLSSDALVVEEPQEQCLQAPAVTAPFFNGADLEQWYLNQQVMEPIPPLIEAEQ

EAGPTPLEDEPAVSLQGSESLAVQQPATELQQREHSQITTEPQQREEKLTQQCQDMPTFV

ANETMTQGEVDVYDASLGDVMVGEHWRESLLNDEQLNSLYLGDWNDDALDFSDFQW

>FvNAc062 FvH4_5g27620.1

MGLLPGQRFCPMDDELLLHYLKPMVNGKIVPGKDSLFCDSDLYGNLEPWQIWQEFESRHV

NDLRKNKDMYFFTKNKKLGSRTCRKVGTGSWKGQGAGNPVYLLDENADPTTTLLGLKKAY

TYVNRNSVHHGHWVMYEFQLHSSQITQLTNDSVLCMLRNNDELPKRKRHQEEEGLEEQSV

EVSAGDSHLSSDALVVEEPQEQCLQAPAVTAPFFNGADLEQWYLNQQVMEPIPPLIEAEQ

EAGPTPLEDEPAVSLQGSESLAVQQPATELQQREHSQITTEPQQREENLTQQCQDMPTFV

ANETMTQGEIDVYDASLGDVMVGEHWRESLLNDEQLNSLYLGDWNDDALDFSDFQW

>FvNAC064 FvH4_5g29430.1

MEPPPVQQSHPHPKASNSVTEATATCGSPSHKPLQQLPHEAAARTRIHHHVANNGASSSA

AQEADHRFSDEEEELNNFNNNQKKQQDAFLSSFPPGYRFNPRDEELVRYYLQKKIMDEPL

PPNKIMEVNLYRHNPETLAGKYDTYGEKEWYFFTPRDRKYRNGKRPNRKASDGYWKATGA

DKSIKSGNVEVGFRKALVFYNGKPPKGKKTNWIMHEYRVPDSRTVKRTENDMRLDNWVLC

RIYKKADNKNKIQEADLAEENISMSILDDTEMEMNGQVDNTQATKSKEEYPHTIAHPPLE

FDRAYSNFAYHYTSYPDQPMIDALSNNMRHHLIQPVAFDSKDSYGNMYHSLPTRGSSSAM

QSLDENCIVPFSEHKHGCSVHLIDMTSESVVCKEEGSDQKYGVQFPGQKYVVQSPEQEKT

HCYGKSVQPSDAVDSRHNPSTPCLDQKSIQQEIGKSLLSPPSDDLPYCIQEPDDEHDNIL

FSDSYFRYSEVFPWV

>FvNAC065 FvH4_5g29460.1

MGLLPGQRFCPMDDELLFPYLKPMVNGQMVPGKDSLFCDSDLYRNLEPWQIWQEFESRHV

NDLRKNKDMYFFIQNKKLGSRTSRKVGTGSWKGQGAGKEVYLLDDNAQPPTILLGLKKTY

TYVNRNSVHHGHWVMYEFQLHSSQITQLTNDCVPCMLRNNDELSKRKRHQEEEGLEEPSV

EASAGDSHLSSDAFVVEEEPQEKRQRLQAPGVTAPFFNGADLEQWYLNQQVMQPIPPLIE

AEQEAGPTPLEDEPAVALQGSENLAVQQPATELQQREPSQITTEPQQREEN

>FvNAC067 FvH4_5g30580.1

MADNPFQLPPGFRFFPTDEELVVHFLQRKAALLPLHPDVIPDLDLYPYDPWELNGKALCE

GKQWYFYSRRTQNRVTSNGYWKPLGTEEPIISSTSNNRVGIKRYFGFYVGEASSGIKTNW

TMHEYRLSSVSDTCASSSTTRSSKRRVHRKAVNEFSGFVLCRVYERNDDEEDDDGTELSC

LDEVFLSLDDLDEISLPN

>FvNAc068 FvH4_5g30600.1

MADNPFQLPPGFRFFPTDEELVVHFLQRKAALLPLHPDVIPDLDLYPYDPWELNGKALCE

GKQWYFYSRRTQNRVTSNGYWKPLGTEEPIISSTSNNRVGIKRYFGFYVGEASSGIKTNW

TMHEYRLSSVSDTCASSSTTRSSKRRVHRKAVNEFSGFVLCRVYERNDDEEDDDGTELSC

LDEVFLSLDDLDEISLPN

>FvNAC066 FvH4_5g30540.1

MGRGMPVGFRFHPTDQELIGHYLSTKTNPNSDPWVSNIDLYGEAEPWTIWEDHGGPLLDD

EDLIFFTKLKKNKQGHRICRRIGSGTWSQGELPKPIFAKNNQETPIGKKTKLSKRPSPSQ

KCQDGRNPKSLRLSDDSITTNEAALSLNQRRRGPNPDRQFDTHDQEHLLLQGNTNAGHVD

YALEQLQRDAVSEHQQQHTCSSSFAQPKTTVVDVFVSENAHSEGTDDCLGVPLEVAQGDT

LFPALSEQQTSFMSQMQAIHSEENESGHARLEDTDWHGIELEAIPERQQPAADQNALVIS

KLQVSTNDEEFSIACDDFNKLPMDDAYINSFEFSNYIFGDEFLSGGDDIGTADILQGSSS

FSDLLEDQHHQV

>FvNAC069 FvH4_5g31040.1

MMDRCTRVVGLRFHPSDQELIGHFLFIKNNPNSVPEMRSSFLREFDMYGEVEPWTIWEAN

GGPLLDDQDLFFFTRLKKHKNRINRRIGSGTWSQGENYKPVYAKNNQETPIGKKTKLRYE

NKEVPEQSGCWFMDEYSAVDDGDYVICRLRENPSKRPSPPSSKPSDKKRKCQHDPEPSKK

PKSTTNEARKTVSLNQRPNPETQFDTMEMFHHDQEYETQQYLLLEGTGLEEVQRDVLAPA

VSEQQHTCSSSFALLNTTNDVFGSENDSGNAQLEDTDWLGDALEDVEGDSLFPALSEQQT

SFMSQMQDIPSQENIIESHIQSPHLPPIGSESQQPADQTGLVISDPQVSTNEEFSFAYDN

FSVPMDPAYNINSDEFSNYNFGDEFLSGGDDIGTADILQGSISFSELLEDQNHEV

>FvNAC070 FvH4_5g34450.1

MSMYYGLRVGMRFHPTDKELVGHYLLNRAVMGDQFQTKFVSGCPGFYGQNEPWVIWDQYG

GNKSDDVEALYFYTHRKKFNPTAKCFDLKVGSGTWREQRSEDDVDEEGSEDVVDKDGSVI

GIRRDYQYEDGCDPHQNGAWLMREYQMKTNTHNNDATDQELVLCVLRKNPEKVQPTTTTA

TAIAKKNGNDAEDWCSGSYTYI

>FvNAC072 FvH4_5g35290.1

MDPTGFRFHPTDQELIGHYLFIKNNSDPVPETGSYLREFDMYGEVEPWTIWEAYGGPLLD

DQDLFFFTRLKKHGHRINRRIGSGTWSQGEPFKSVYAKNNQETPIGKKTKLRYENKNVTE

QSGCWFMDEYSTVDYDGDYVICRLRENPGKRPSPPPSSKPSDNKRKCQAESRPSKKTKSL

RVSDPQPTIITNEAGKTVSLNQRPNPEKQFDQSLRLPVPDSQAMFLEGTTAAGHIDCAFD

TVEMFHHDQEYETQHFLLEGNTAGHSDYALVEVQRDALSPAAVSEQQHTCASSFAEPNLT

DDVFVSENERGNEQLEDTDCLGVALGVVEGDTLFSALSEQQTSLMSQMQDIPSQENIIES

HIQSPYVPHLGSEYQQQADTNEEEFGLAYDDDFSNLPMDPAYINSDEFNNYIFGDEFLSG

GDDIGTADIVQGYSSFSALLEDQNHEV

>FvNAC071 FvH4_5g34910.1

MDPTGFRFHPTDQELIGHYLFIKNNSDPVLETGSYLREFDMYGEIESWTIWEAYGGPLLD

DQDLFFFTRLKKHGHRINRRIGSGTWSQGEPFKSVYAKNNQETPIGKKTKLRYENKNVTE

QSGCWFMDEYSTVDYDGDYVICRLRENPDKRPSPPPLSKPSDKKRKCQADPKPSKKTKSL

RVSDPQPATITNEARKTVSLNQRPNPEKRFDQSLRLPDSHVMLLEGTTAAGHIDCAFDTV

EMFHHDQEYETQHFLLEGNTAGHSDYALVEVQRDAFSPAAVSEQQHTCASSFALPNLTDD

VFVSENESGNQQLEDTDCLGVALGNINKQIPMIKEFAFAYDDDFSNLPMDPAYINSDEFS

SYIFGDEFLAGGDDIGTTNILQGSSSFSALLEDQNHEV
