# Supplementary material for: Genome-wide analysis of the NAC transcription factor family and their expression during the development and ripening of the Fragaria × ananassa fruits
Source: PLoS One. 2018 May 3;13(5):e0196953. doi: 10.1371/journal.pone.0196953 (PMC5933797; doi:10.1371/journal.pone.0196953)
Supplement: S8 Table — (DOCX) [file pone.0196953.s008.docx]

| **Motifs** |
| --- |
| **Motif 1:** 50 sites, 43aa, E-value 5.1E-1320  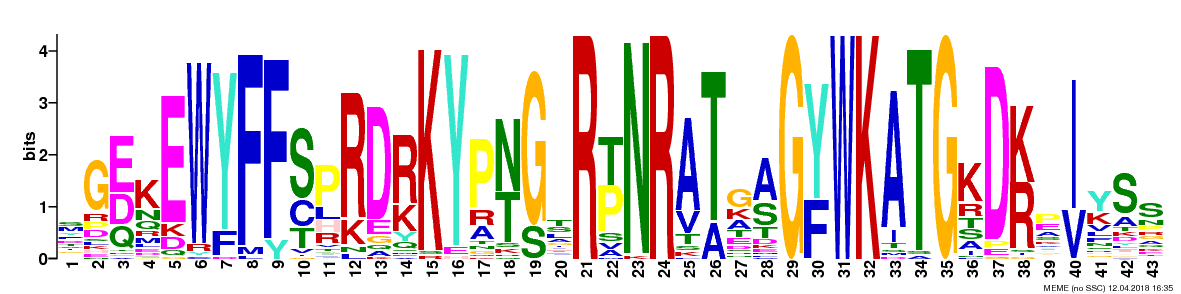 |
| **Motif 2:** 50 sites, 31aa, E-value 3.6E-1067  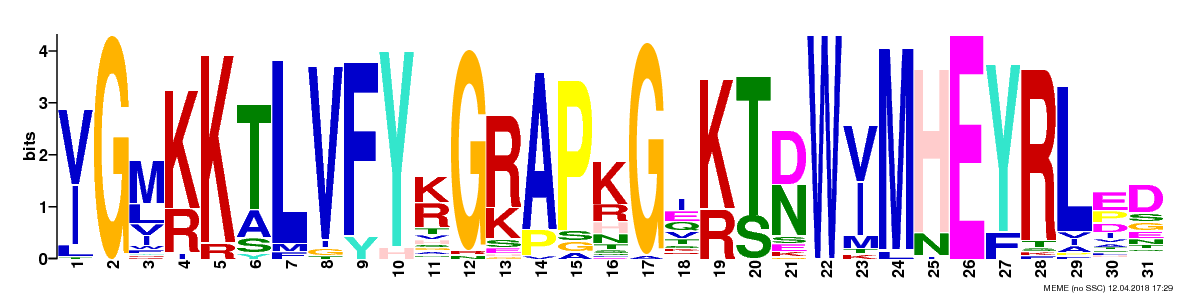 |
| **Motif 3**: 50 sites, 22aa, E-value 6E-712  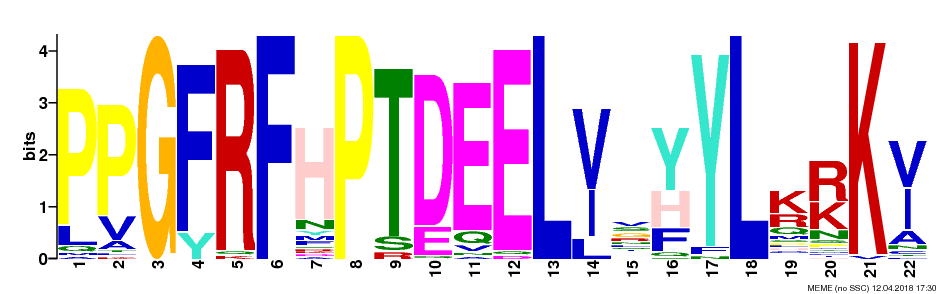 |
| **Motif 4:** 14 sites, 84aa, E-value 2.7E-683  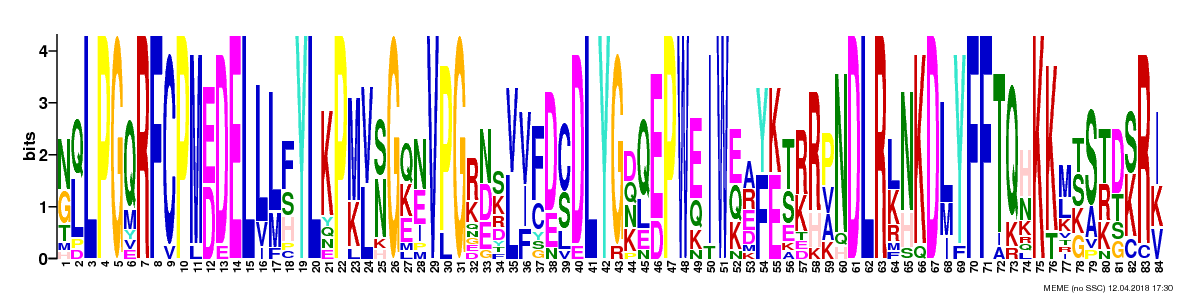 |
| **Motif 5**: 50 sites, 22aa, E-value 3.3E-477  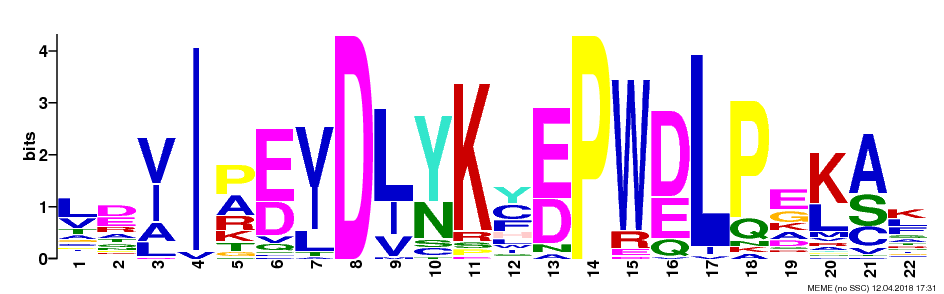 |
| **Motif 6:** 23 sites, 43aa, E-value 1.1E-347  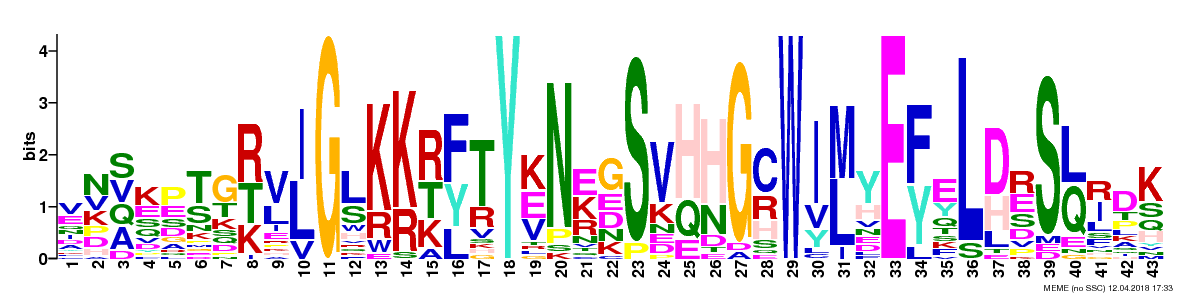 |
| **Motif 7:** 41 sites, 16 aa, E-value 2.2E-291  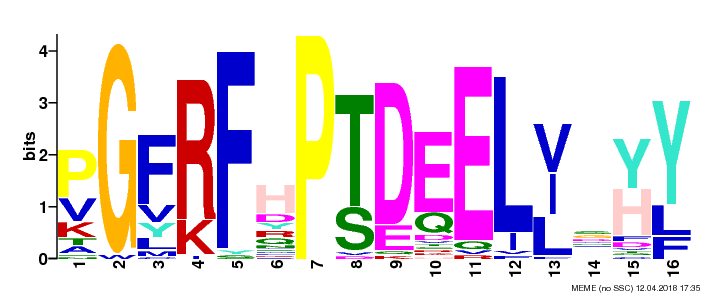 |
| **Motif 8:** 50 sites, 22aa, E-value 1E-273  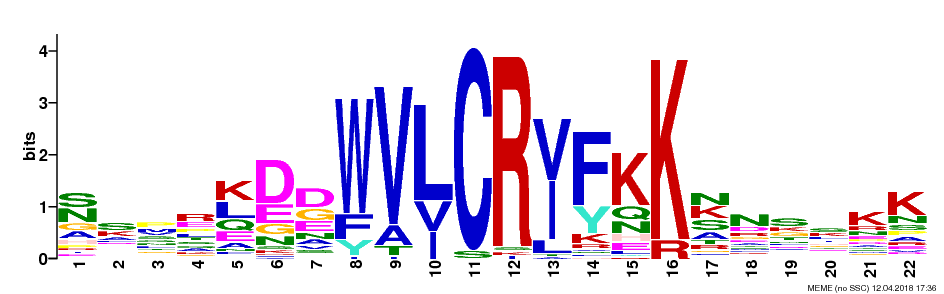 |
| **Motif 9:** 7 sites, 49 aa, E-value 2.7E-174  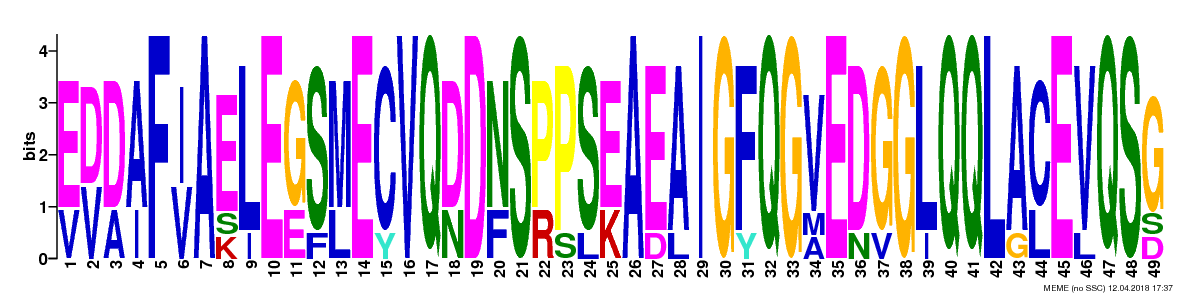 |
| **Motif 10:** 4 sites, 84aa, E-value 4.5E-159  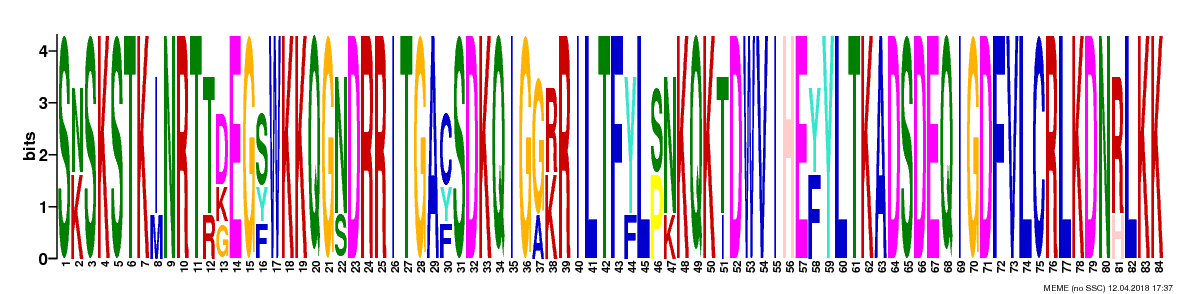 |
| **Motif 11:** 34 sites, 14aa, E-value 9.4E-125  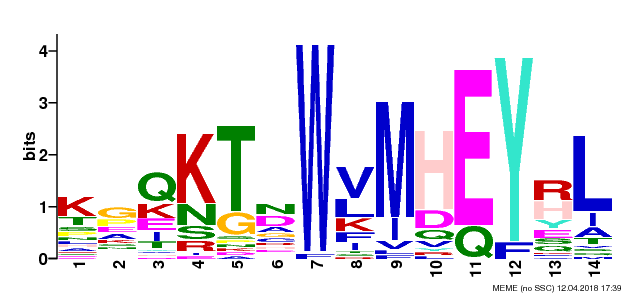 |
| Motif 12: 25 sites, 22 aa, E-value 6.5E-124  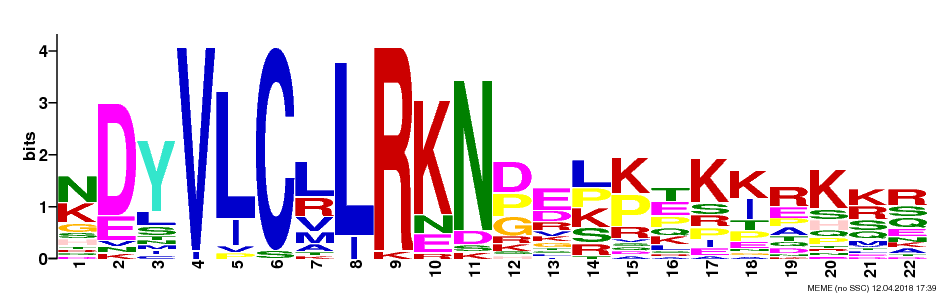 |
| **Motif 13:** 5 sites, 66aa, E-value 2.4E-110  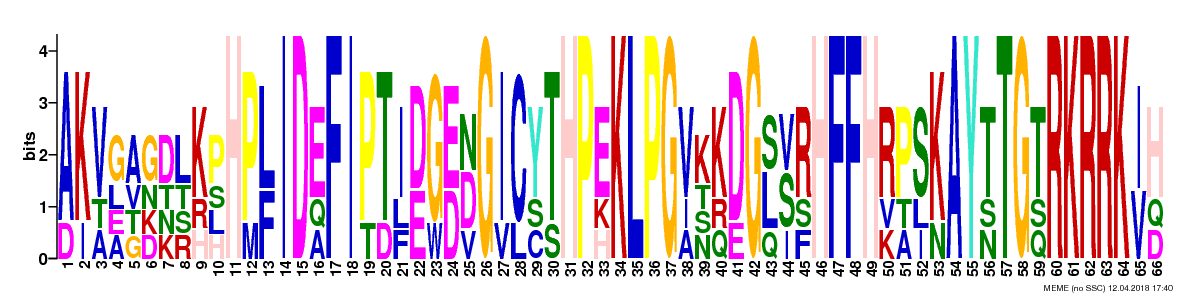 |
| **Motif 14:** 7 sites, 31aa, E-value 2.4E-106  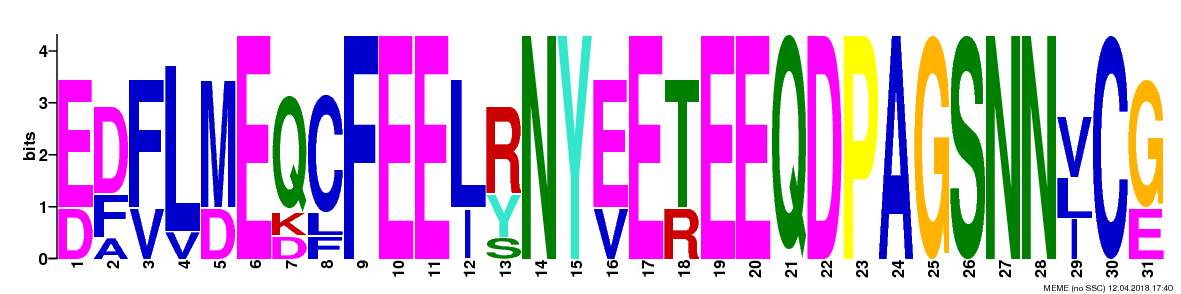 |
| **Motif 15:** 86 sites, 22aa, E-value 4.8E-97  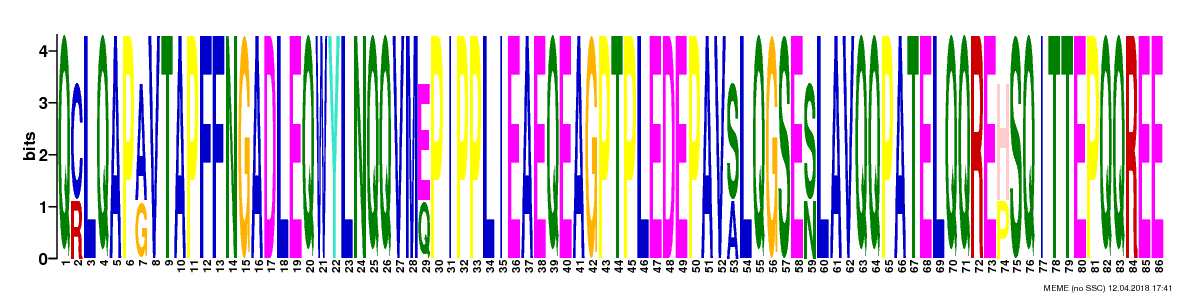 |

**S8 Table**. **Amino acid consensus sequences found in the motifs discovered by the MEME program**.
