# Supplementary figures and images for: Genome-wide analysis of the NAC transcription factor family and their expression during the development and ripening of the Fragaria × ananassa fruits
Source: PLoS One. 2018 May 3;13(5):e0196953. doi: 10.1371/journal.pone.0196953 (PMC5933797; doi:10.1371/journal.pone.0196953)

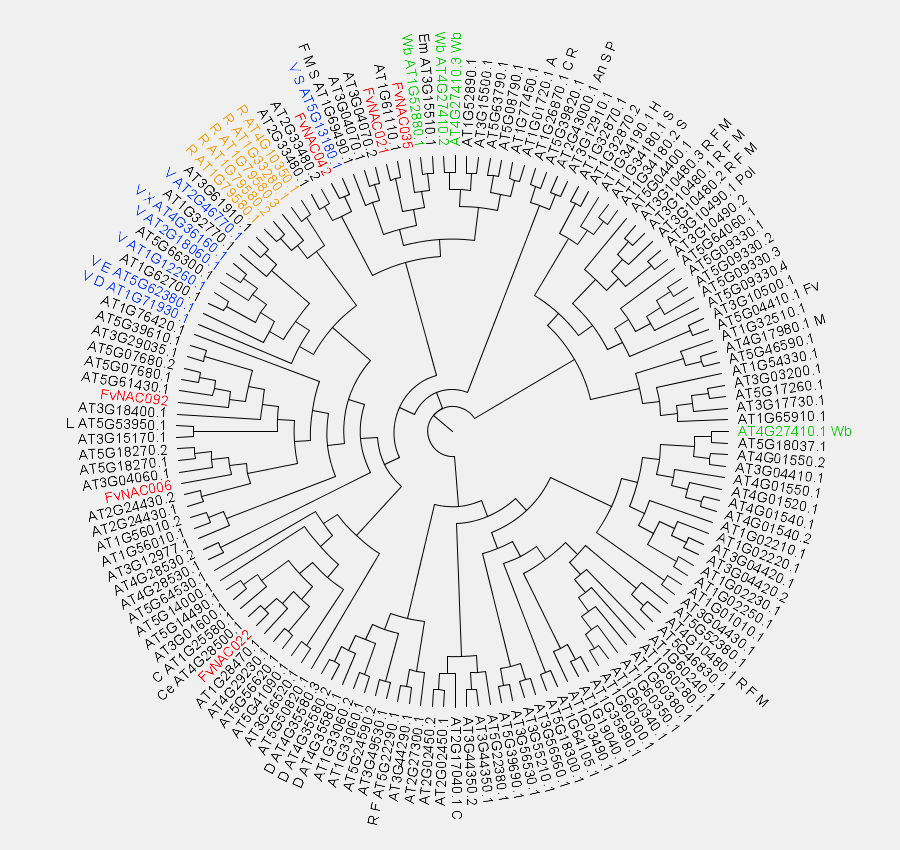

Supplement: S1 Fig — Red: Fragaria vesca sequences; Blue: Arabidopsis genes related to the biosynthesis and/or development of vascular tissue and cell-walls; Green: Arabidopsis genes related to water balance and/or stress. Biological functions are defined at the beginning or the end of the protein name: S: senescence; D: defense; M: Meristem formation; Fv: Flavonoid biosynthesis; H: Response to hydrogen peroxide; R: Root cap; C: Cell division; Ce: Cell size; Pol: Pollen development; A: ABA response; F: Flowering; E: Epistasis; P: Proline; RF: Flowering repression; V: biosynthesis and/or development of vascular tissue and cell-walls; Dh: Fruit Dehiscence; An: Anthocyanin related; Wb: water balance and/or stress; L: Leaf formation; Em: Embryo development; G: DNA damage. In S4 and S7 Tables, we have included live URL links that provides the correspondence between Arabidopsis protein names and their corresponding UniProt accession. (TIF) [file pone.0196953.s013.TIF]

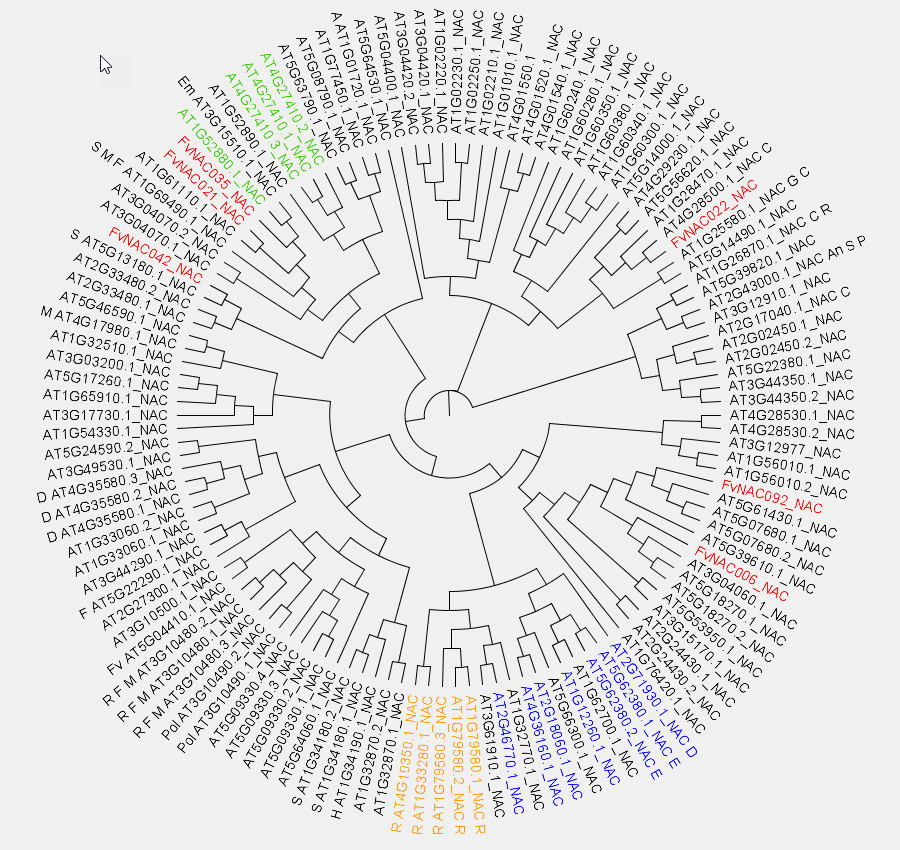

Supplement: S2 Fig — Red: Fragaria vesca sequences; Blue: Arabidopsis genes related to the biosynthesis and/or development of vascular tissue and cell-walls; Green: Arabidopsis genes related to water balance and/or stress. Biological functions are defined at the beginning or the end of the protein name: S: senescence; D: defense; M: Meristem formation; Fv: Flavonoid biosynthesis; H: Response to hydrogen peroxide; R: Root cap; C: Cell division; Ce: Cell size; Pol: Pollen development; A: ABA response; F: Flowering; E: Epistasis; P: Proline; RF: Flowering repression; V: biosynthesis and/or development of vascular tissue and cell-walls; Dh: Fruit Dehiscence; An: Anthocyanin related; W: water balance and/or stress; L: Leaf formation; Em: Embryo development; G: DNA damage. In S4 and S7 Tables, we have included live URL links that provides the correspondence between Arabidopsis protein names and their corresponding UniProt accession. (TIF) [file pone.0196953.s014.TIF]

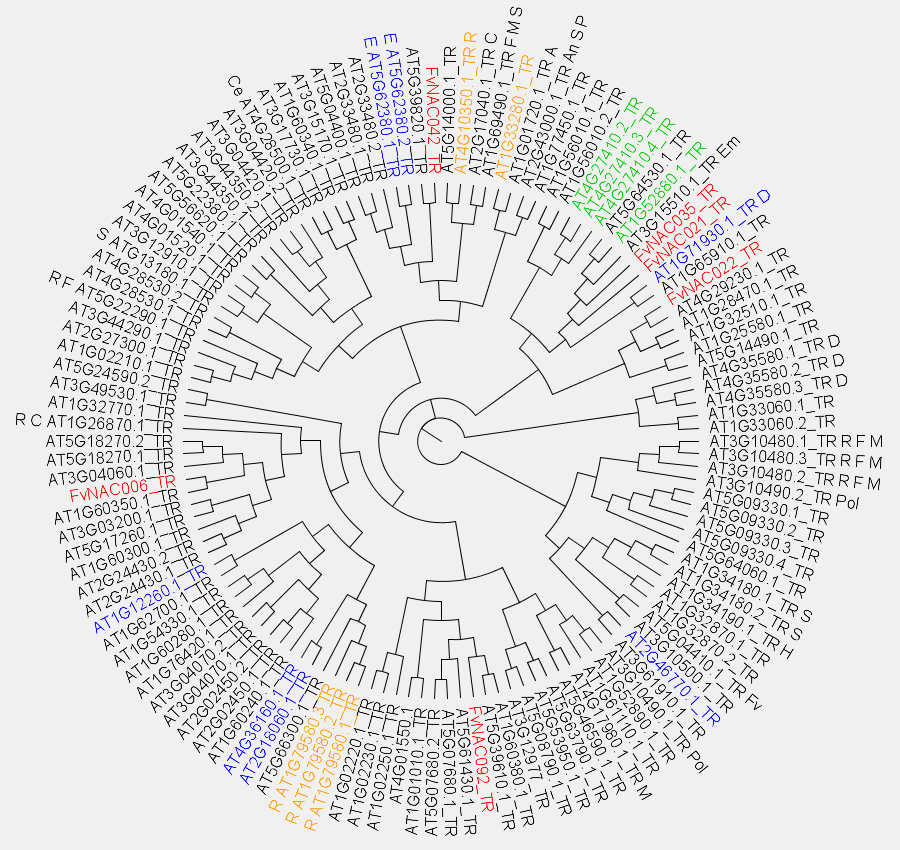

Supplement: S3 Fig — Alignment of only the C-terminal portion of the NAC proteins containing the TR (transcriptional regulatory). Red: Fragaria vesca sequences; Blue: Arabidopsis genes related to the biosynthesis and/or development of vascular tissue and cell-walls; Green: Arabidopsis genes related to water balance and/or stress. Biological functions are defined at the beginning or the end of the protein name: S: senescence; D: defense; M: Meristem formation; Fv: Flavonoid biosynthesis; H: Response to hydrogen peroxide; R: Root cap; C: Cell division; Ce: Cell size; Pol: Pollen development; A: ABA response; F: Flowering; E: Epistasis; P: Proline; RF: Flowering repression; V: biosynthesis and/or development of vascular tissue and cell-walls; Dh: Fruit Dehiscence; An: Anthocyanin related; W: water balance and/or stress; L: Leaf formation; Em: Embryo development; G: DNA damage. In S4 and S7 Tables, we have included live URL links that provides the correspondence between Arabidopsis protein names and their corresponding UniProt accession. (TIF) [file pone.0196953.s015.TIF]
